# High efficacy of BPaL among patients infected with *Mycobacterium tuberculosis* lineage 1 in the Philippines

**DOI:** 10.5588/ijtldopen.25.0677

**Published:** 2026-04-13

**Authors:** I. Flores, M.A.A. Tujan, R. Basilio, T.J.R. Dizon, D.R Lim, L.T. Reyes, R.V.A.G. Reyes, S. Rojas, A.K. Valencia, J.B. Capin, V. Mirtskhulava, A.G. Palparan, C. Cabalitan, P.J. Carpin, C. Malbacias, J.S. Lee, A. Gebhard, M. Quelapio, J. Timm

**Affiliations:** 1 Tropical Disease Foundation, Makati City, Philippines;; 2 Jose B. Lingad Memorial General Hospital, San Fernando City, Philippines;; 3 Department of Health, Research Institute for Tropical Medicine, Muntinlupa, Philippines;; 4 KNCV Tuberculosis Foundation, The Hague, the Netherlands;; 5 Faculty of Natural Sciences and Medicine, Ilia State University, Tbilisi, Georgia;; 6 Philippine Business for Social Progress, Mandaluyong City, Philippines;; 7 Department of Health, National TB Program, Disease Prevention and Control Bureau, City of Manila, Philippines;; 8 International Tuberculosis Research Center, Changwon, Republic of Korea;; 9 TB Alliance, New York, NY, USA.

**Keywords:** tuberculosis, MDR/RR-TB treatment, anti-TB drug resistance, Southeast Asia

## Abstract

**BACKGROUND:**

WHO recommends the bedaquiline–pretomanid–linezolid regimen with/without moxifloxacin (M) (BPaL/M) for the treatment of multidrug- or rifampicin-resistant TB. However, *Mycobacterium tuberculosis* (MTB) lineage 1 (L1) is less susceptible to pretomanid than other lineages, and there are limited BPaL/M efficacy data from regions where L1 is prevalent.

**METHODS:**

We performed whole genome sequencing (WGS) on baseline MTB isolates from a subgroup (34/103) of patients from the highly successful Philippine BPaL Operational Research Study to characterise their lineage and genotypic drug susceptibility testing (DST) profile. Phenotypic DST for BPaL drugs was also conducted.

**RESULTS:**

WGS analysis showed that L1 (68%) predominated, followed by L4 (26%) and L2 (6%). Out of the 22 L1 isolates tested, 20 exhibited higher pretomanid minimum inhibitory concentrations than isolates from other lineages, including one with borderline resistance. Two patients had confirmed bedaquiline resistance; no linezolid resistance was detected. All 34 patients with characterised isolates had culture converted by end of treatment. At month 12 follow-up, 30/31 patients who provided sputum remained culture negative; the single culture-positive participant harboured MTB L4.

**CONCLUSION:**

In this study, patients infected with MTB L1 responded to BPaL as well as those infected with other lineages. Baseline bedaquiline resistance was linked to the unique recurrence in the study.

Drug resistance is a major barrier to TB control. In 2023, an estimated 400,000 people developed multidrug- or rifampicin-resistant TB (MDR/RR-TB) globally. Treatment regimens for drug-resistant (DR) TB have historically achieved low success rates due to their long duration and inclusion of poorly tolerated, relatively ineffective drugs.^1^ In 2019, the WHO issued a Rapid Communication recommending the use of a 6-month, all oral regimen consisting of bedaquiline plus pretomanid plus linezolid (BPaL) under operational research (OR) conditions for selected DR-TB patients without prior exposure to bedaquiline or linezolid.^2^ In 2022, WHO released the consolidated DR-TB treatment guidelines recommending BPaL with or without moxifloxacin (BPaL/M) as the regimen of choice for eligible MDR/RR-TB patients under programmatic conditions.^3^ The Philippines accounts for the fourth largest TB burden in the world and a large contingent of DR-TB, with an estimated 29,000 new MDR/RR-TB in 2023.^4^ A BPaL OR Study was initiated in the Philippines in 2021, with the support of the ‘Leveraging Innovation for Faster Treatment of Tuberculosis (LIFT-TB)’ initiative.^5^ From June 2021 to December 2022, 103 patients with pre-extensively drug-resistant TB (pre-XDR-TB), MDR/RR-TB intolerant, or non-responsive to a previous MDR-TB regimen were enrolled in the study, and ultimately 96 were retained for the efficacy and safety analyses. The treatment success rate (TSR) was 98%, with sputum culture conversion at 80% and 94%, at treatment months 1 and 4, respectively. Sustained success at 6 and 12 months post-treatment was 92% and 90%, respectively.^6^ The Philippine BPaL OR Study provided the opportunity to directly test if the reduced susceptibility of *Mycobacterium tuberculosis* lineage 1 (MTB L1) to pretomanid^7^ could lead to lower BPaL efficacy in countries where MTB L1 is prevalent (‘high L1 countries’). Indeed, most TB cases in the Philippines are caused by this clade (73% in one study).^8^

Here, we performed analysis of whole genome sequencing (WGS) from baseline isolates in a representative subset of Philippine BPaL OR Study patients to determine their lineage and relate this information to treatment outcomes, as well as to infer their relatedness to MTB strains circulating in the country. Finally, we conducted phenotypic and genotypic drug susceptibility testing (DST) to BPaL drugs on the same isolates to investigate if baseline resistance had an effect on treatment outcomes.

## METHODS

### DNA extraction and sequencing

All 58 baseline isolates from the Philippine OR Study were grown on Ogawa media and subjected to DNA extraction by the phenol/chloroform method at the National TB Reference Laboratory. DNA samples were transferred to the Research Institute for Tropical Medicine for WGS. DNA quality and concentration were determined using a Qubit fluorometer (Life Technologies Holdings Pte Ltd, Singapore) and a NanoDrop spectrophotometer (Thermo Fisher Scientific, Waltham, USA), following the manufacturer’s instructions. Twenty-four DNA samples were deemed of poor quality and not analysed further. Those that passed quality control (QC) were then subjected to DNA library preparation and sequencing using both Illumina MiSeq (Illumina, San Diego, USA) and Oxford Nanopore Technologies MinION Mk1B (Oxford, UK) platforms, following manufacturer’s instructions, and hybrid genomes were assembled. All raw sequencing data are publicly available at the European Nucleotide Archive (see Supplementary Data Table S1 for accession numbers).

### Bioinformatic analysis

Reads were analysed using a bioinformatic pipeline for QC, genotypic resistance prediction, sample relatedness characterisation, and phylogenetic reconstruction. In short, QC included read trimming and removal of any non-MTB reads; all 34 sequences had ≥52× median read depth (Supplementary Data Table S1). TB-Profiler v6 was then used to characterise genotypic resistance using the WHO Catalogue of Drug Resistance Mutations, second edition^9^ – relevant genetic polymorphisms are listed in Supplementary Data Table S2 – and to characterise sample relatedness via single nucleotide polymorphism (SNP)-distance calculation. Finally, a concatenated SNP-alignment was constructed and used to perform phylogenetic reconstruction.

### Phenotypic susceptibility testing

Pretomanid minimum inhibitory concentrations (MICs) and testing for bedaquiline and linezolid at their critical concentration (CC; 1 mg/L for both drugs) were conducted in a Mycobacterial Growth Indicator Tube™ (MGIT) system (Becton & Dickinson, Franklin Lakes, USA) as described previously.^10^ Phenotypic DST was also performed for select first-line drugs (FLDs; rifampicin, isoniazid, and ethambutol) and second-line drugs (SLDs; levofloxacin, moxifloxacin, and amikacin) using the Löwenstein–Jensen proportion or MGIT methods.

### Ethical approval

Informed consent for the BPaL OR Study was obtained from all subjects and/or their legal guardian(s) and approved by the Single Joint Research Ethics Board of the Philippines, and the approval included bio-banking of MTB isolates for possible future research studies.

## RESULTS

### Patient subgroup with characterised baseline MTB isolates

**Figure 1.**
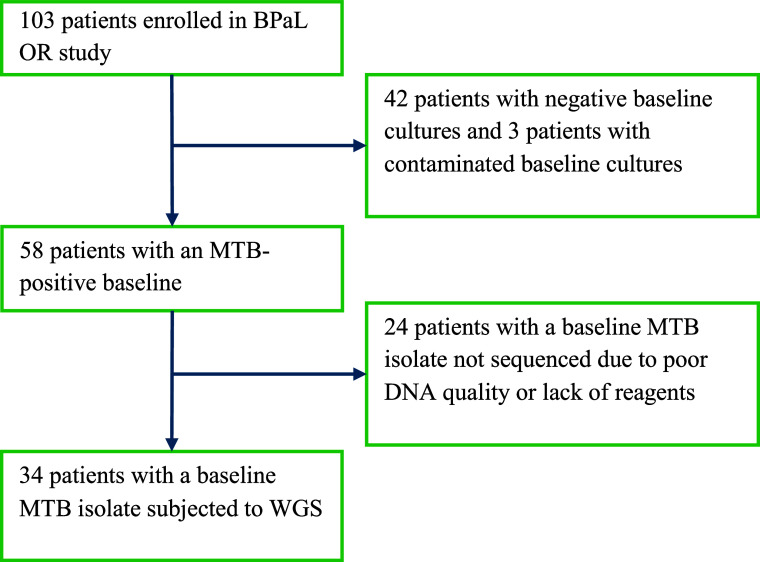
Disposition of baseline MTB isolates.

**Figure 2.**
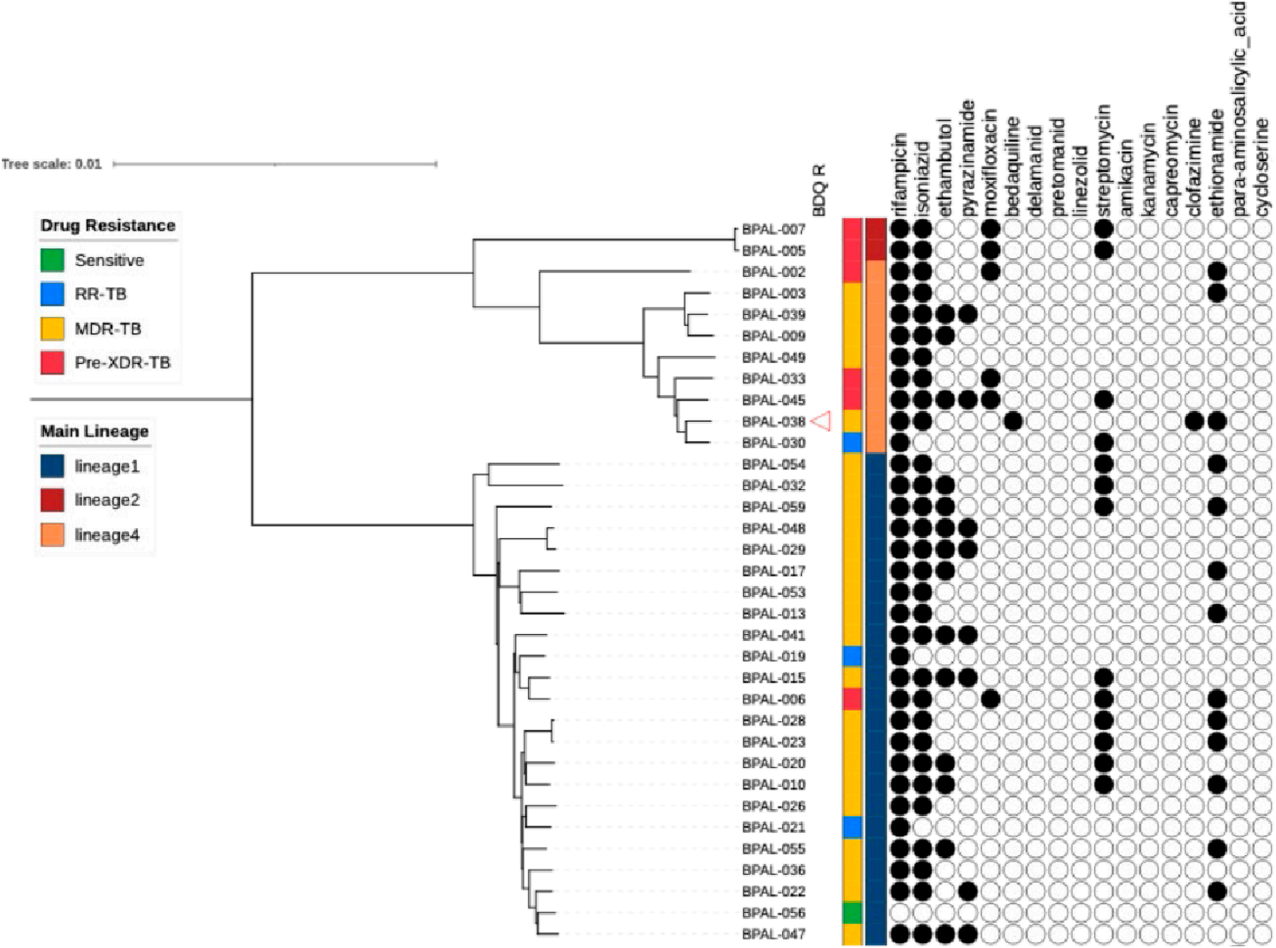
Phylogenetic reconstruction and drug susceptibility profile based on WGS. The phylogeny was generated using a maximum likelihood approach implemented in IQ-TREE with the GTR+G+ASC substitution model. Drug-resistance type and lineage are indicated with colour strips. Individual drug resistance as determined genotypically with TB-profiler is visualised with filled circles representing resistance and circle outlines representing susceptibility. Resistance to bedaquiline (BDQ) is further highlighted.

Of the 103 patients enrolled in the Philippine OR Study, 34 had their baseline MTB isolates characterised by WGS (Figure 1). This group included seven with suspected bedaquiline resistance, based on preliminary phenotypic DST results (discussed below), who were hence excluded from the published efficacy analysis (n = 96).^6^ Lineage imputation revealed that the majority of the 34 patients harboured an L1 isolate (23, 68%) followed by L4 (9, 26%) and L2 (2, 6%) (Figure 2).

When compared to the overall study patient population – with or without a corresponding sequenced MTB isolate – the subgroup of those harbouring an L1 isolate was not significantly different with respect to their baseline characteristics, except perhaps for HIV status and fluoroquinolone resistance (see Table). HIV+ status has been linked to worse TB treatment outcomes in rural Africa, but this seems related to barriers to antiretroviral access,^10,11^ which was not the case in the Philippine OR Study. And the mechanism of resistance to fluoroquinolones (mutations in *gyrB-gyrA* genes) does not lead to cross-resistance to BPaL drugs,^12^ even though baseline fluoroquinolone resistance has been shown to be a risk factor for bedaquiline resistance acquisition.^13^

**Table.** Baseline characteristics and treatment success rates of the patients.

	Total analysed^A^	Subgroup with MTB L1
A. Characteristic		
Number of patients	96	23
Median age (IQR) – years	42 (22.2)	46 (15.5)
Male sex – n (%)	63 (65%)	15 (65%)
Median body mass index (IQR)	20.05 (5.5)	20 (4.4)
Previous treatment – n (%)	93 (97%)	15 (65%)
Fluoroquinolone resistance – n (%)	40 (42%)	5 (22%)
Cavitary TB – n (%)	30 (31%)	6 (26%)
Unilateral	14 (14.5%)	3 (13%)
Bilateral	16 (16.5%)	3 (13%)
Diabetes – n (%)	40 (42%)	11 (48%)
HIV positive – n (%)	8 (8%)	0
Karnofsky score 80–100 – n (%)	90 (94%)	23 (100%)
B. Success rate		
End of treatment	94/96 (98%)	23/23 (100%)
6-month follow-up	86/94 (92%)	20/20 (100%)
12-month follow-up	84/93 (90%)	21/21 (100%)

IQR = interquartile range; MTB L1 = *Mycobacterium tuberculosis* lineage 1.

A As reported by Flores et al.^6^

Comparing TSR among the L1 subgroup (100%) was as high as that of the total patient population (98%) (see Table). And the only participant with a sequenced isolate who had a recurrence at 6-month follow-up harboured MTB L4 (Supplementary Data Table S1). Therefore, in the subgroup of nine patients with MTB L4, TSR was 100% (9/9) but sustained success at 12-month follow-up was 89% (8/9); while in the two patients with L2, both rates were equal to 100%.

### Phenotypic DST

Before WGS data became available, phenotypic DST for bedaquiline, linezolid, and pretomanid was performed on 33/34 baseline isolates using the MGIT system (Supplementary Data Table S1). In the case of bedaquiline and linezolid, at the time, there were already WHO-recommended CCs (1 mg/L for both drugs)^14^; thus, the mycobacteria were only challenged at that concentration. In contrast, for pretomanid, WHO had not yet published a CC recommendation, and a range of concentrations were tested, leading to actual MIC determination. This initial analysis identified seven participants with bedaquiline resistance who remained on treatment but were excluded from the efficacy assessment, as pre-specified in the Study Protocol. All seven participants had cultures negative for MTB at the end of treatment (‘cured’) and only one (BPAL-038) had recurrent TB at month 6 follow-up – herein, isolate and patient identifiers are identical. More recently, repeat MGIT DST confirmed baseline bedaquiline resistance for BPAL-036 and BPAL-038 (Figure 2) (Supplementary Data Table S1). Two out of seven could not be retested due to lost viability. Notably, had the seven patients with unconfirmed baseline bedaquiline resistance been included in the original outcome analysis, success rates would have not changed.

All baseline isolates tested were susceptible to linezolid. As expected, 20/22 L1 isolates tested for pretomanid had MICs ≥1 mg/L, whereas isolates from other lineages exhibited MICs between 0.125 and 0.5 mg/L. One L1 isolate, BPAL-059, had a MIC two-fold higher than the now-recommended pretomanid CC (2 mg/L) for this lineage.^14^ BPAL-059 sputum culture converted during treatment and remained negative in follow-up (Supplementary Data Table S1).

Phenotypic DST for select FLDs and SLDs revealed a few discrepancies with DST data obtained prior to screening for this study, as well as with genotypic DST (discussed below). Most notably, participant BPAL-056 was found to be sensitive to all drugs tested (Figure 2) (Supplementary Data Table S1).

### Genotypic DST and isolate relatedness

**Figure 3.**
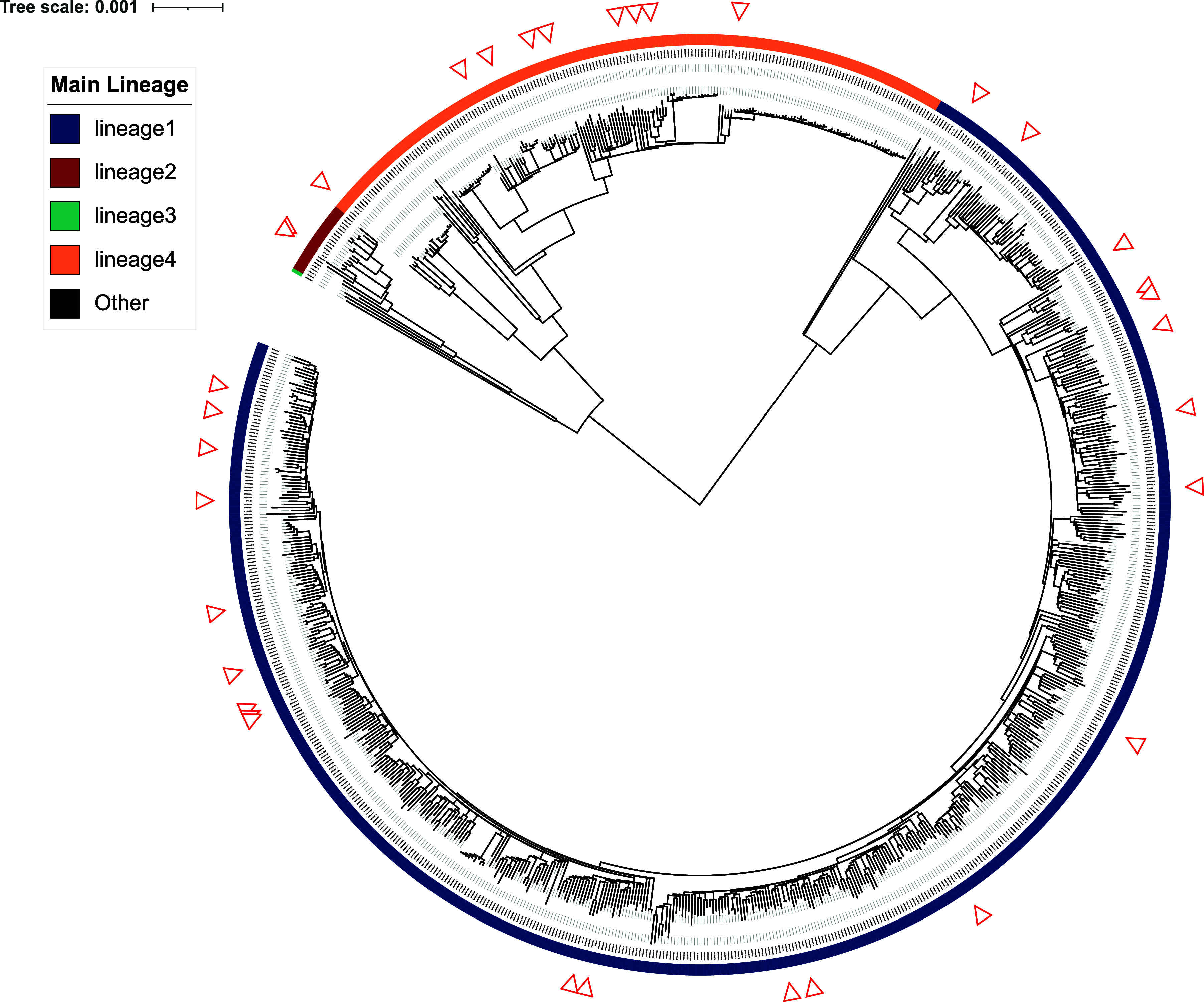
Genetic relatedness of MTB isolates characterised in this study to strains circulating in the Philippines. The phylogeny was generated using isolates from this study plus all publicly available genome sequences from the Philippines (as of August 2025). Reconstruction was performed using a maximum likelihood approach implemented in IQ-TREE with the GTR+G+ASC substitution model. Lineage is annotated with the coloured strip around the outside of the tree. The location of isolates from this study in the wider phylogeny is indicated with the red triangles.

TB-profiler was used to predict drug resistance and calculate sample-relatedness (Figure 1). In addition, polymorphisms associated with resistance (or hyper-susceptibility) to bedaquiline and pretomanid were manually screened in sequences corresponding to isolates with phenotypic resistance as indicated by initial DST. Mutations in a canonical bedaquiline resistance gene were found in BPAL-038 (*mmpR5* c.144dupC, c.321dupC, and c.382dupG) but not in BPAL-036. No mutation in any of the canonical pretomanid resistance genes (*ddn*, *fbiA*-*D*, *fgd1*) was found in BPAL-059. SNP-distance analysis of isolates from this study together with all publicly available genome sequences from the Philippines, including a set of 784 previously published,^15^ confirmed that our study’s group is representative of strains circulating in the country (Figure 3).

## DISCUSSION

*M. tuberculosis* has been classified into 10 lineages mainly on the basis of large-scale genomic variations.^16,17^ Lineages 1 to 4 (L1–L4) have been isolated all around the world, but L1 (or the India-Oceanic lineage) is more prevalent in high-burden countries around the Indian Ocean. It has been estimated that, globally, 28% of TB cases in 2012 and 2018 were due to L1.^18^ In 2022, Bateson and collaborators showed that MTB L1 is intrinsically four-fold less susceptible to pretomanid than other lineages.^7^ Their findings have now been replicated by other groups, using other DST methods.^19^ Yet, the molecular mechanism(s) behind this reduced susceptibility to pretomanid, but not to the other approved nitroimidazole drug delamanid, remains unknown.

Since the successful clinical trials that constituted the scientific basis for the approval of pretomanid by the regulatory agencies from several countries, as well as the WHO recommendation of BPaL/M as the preferred MDR/RR-TB regimen, enrolled predominantly TB patients infected with MTB L2–L4^10^ (T. McHugh, personal communication), it has been argued that trial results may not be generalisable to high L1 countries.^14,20^ Aiming to answer this question, WHO issued a public call in April 2023 for data on pretomanid MIC distributions across MTB lineages and treatment outcomes. The WHO analysis of the submitted data was limited to very few L1-infected patients and hence inconclusive.^14^ Since then, a trial in India^21^ and OR studies in other high-L1 countries, all with success rates >82%,^5,22,23^ have been reported. These investigations, however, did not include lineage data. Here, we determined the lineage for the isolates from a representative subset of 34 patients in the Philippine OR Study and showed that 23/34 (68%) were infected with MTB L1 and yet exhibited very high success rates at end of treatment (23/23) and in the follow-up period up to 12 months (21/21).

We also investigated baseline resistance to bedaquiline, pretomanid, and linezolid among the 34 patients. MGIT DST for bedaquiline revealed resistance in seven patients, initially. However, repeat testing using the same method and genotypic DST confirmed resistance in two (6%). This highlights the low accuracy of bedaquiline phenotypic DST at the currently WHO-recommended CC and the importance of a composite standard (phenotypic plus genotypic) or, at least, repeat testing of resistant isolates.^24^ Testing for pretomanid also uncovered an isolate with a MIC two-fold (MIC = 4 mg/L) above the CC. This ‘borderline’ phenotype has rarely been seen, and its clinical relevance is unknown; typical pretomanid-resistant MTB exhibits MICs >16 mg/L. No patient with linezolid resistance was identified. Overall, our study highlights the importance of resistance surveillance for BPaL drugs in the Philippines and elsewhere; emerging bedaquiline resistance, in particular, is now recognised as a challenge for DR-TB control.^25^

A limitation of our study is sample size. Only 34 isolates were characterised, from a BPaL OR study that enrolled 103 patients.^6^ However, the lineage composition of our MTB collection is similar to the pool of strains circulating in the Philippines.^8^ Thus, we submit that the high efficacy of the BPaL regimen seen in our subgroup of patients with MTB L1 is generalisable to the Philippine population with MDR/RR-TB, if treated under the confines of an OR study which involves more rigorous patient monitoring. Programmatic data from the Philippines and other countries implementing BPaL/M are urgently needed to confirm these results.

## Supplementary Material
