# Supplementary material for: High efficacy of BPaL among patients infected with Mycobacterium tuberculosis lineage 1 in the Philippines
Source: IJTLD Open. 2026 Apr 13;3(4):226–31. doi: 10.5588/ijtldopen.25.0677 (PMC13080305; doi:10.5588/ijtldopen.25.0677)
Supplement: Supplementary file 1 [file ijtldopen25-0677_supplementarydata1.pdf]

Supplementary Table 1: Patient outcome and microbiology data

Identifiers			Treatment outcome					Phenotypic DST										WGS QC	ENA accession no.
REDCap ID	BPAL ID	WGS lab ID	EoT	6M FU	12M FU	Remarks	Lineage	BDQ	PMD MIC (mg/L)	LZD	INH	RIF	EMB	STM	MFX	LFX	AM	Hybrid genome coverage	
079	002	PN-22-7134	Cured	Culture ND	Sustained success		4.1.2.1	S	0.25	S	R	R	S	R	R	R	S	127X	ERS28168310
004	003	PN-21-7148	Cured	Sustained success	Sustained success		4.3.4.2	S	0.25	S	ND	ND	ND	ND	ND	ND	ND	74X	ERS28168311
124	005	PN-22-10974	Cured	Sustained success	Sustained success		2.2.1	S	0.5	S	R	R	S	R	R	R	S	79X	ERS28168312
100	006	PN-22-9664	Cured	Sustained success	Culture ND	Died due to CVA a month prior to 12M F	1.2.1.2.1	S	2	S	R	R	S	R	R	R	S	84X	ERS28168313
087	007	PN-22-9660	Cured	Sustained success	Sustained success		2.2.1	S	0.25	S	R	R	S	S	R	R	S	67X	ERS28168314
064	009	PN-22-7985	Cured	Sustained success	Sustained success		4.3.4.2	S	0.25	S	R	R	S	R	S	S	S	100X	ERS28168315
067	010	PN-22-6624	Cured	Sustained success	Sustained success		1.2.1.2.1	ND	ND	ND	R	R	R	S	S	S	S	94X	ERS28168316
062	013	PN-22-6625	Cured	Sustained success	Sustained success		1.2.1.2.1	S	2	S	R	R	R	R	S	ND	S	66X	ERS28168317
069	015	PN-22-8765	Cured	Sustained success	Sustained success		1.2.1.2.1	S	2	S	R	R	R	R	S	S	S	86X	ERS28168318
063	017	PN-22-6279	Cured	Culture ND	Sustained success	Failed to come for 6M FU	1.2.1.2.1	S	1	S	R	R	S	S	S	S	S	52X	ERS28168319
115	019	PN-22-11028	Cured	Sustained success	Sustained success		1.2.1.2.1	S	1	S	S	R	R	R	S	S	S	110X	ERS28168320
107	020	PN-22-9563	Cured	Sustained success	Sustained success		1.2.1.2.1	S	2	S	R	S	S	S	S	S	S	78X	ERS28168321
093	021	PN-22-8731	Cured	Culture ND	Sustained success	Unable to produce sputum for 6M FU	1.2.1.2.1	S	0.25	S	S	R	R	R	S	S	S	129X	ERS28168322
086	022	PN-22-7513	Cured	Sustained success	Sustained success		1.2.1.2.1	S	1	S	ND	ND	ND	ND	ND	ND	ND	137X	ERS28168323
057	023	PN-22-7030	Cured	Sustained success	Sustained success		1.2.1.2.1	S	1	S	R	S	S	S	S	S	S	108X	ERS28168324
044	026	PN-22-4530	Cured	Sustained success	Sustained success		1.2.1.2.1	S	1	S	R	R	S	S	S	ND	S	348X	ERS28168325
045	028	PN-22-3333	Cured	Sustained success	Sustained success		1.2.1.2.1	S	1	S	R	R	R	S	S	S	S	197X	ERS28168326
039	029	PN-22-3393	Treatment Completed	Sustained success	Sustained success		1.2.1.2.1	S	1	S	R	R	S	S	S	S	S	218X	ERS28168327
084	030	PN-22-9610	Cured	Culture ND	Culture ND	Uncontactable during 6M FU. Culture ND	4.3.4.1	S	0.125	S	R	S	S	R	S	S	S	76X	ERS28168328
051	032	PN-22-5099	Cured	Sustained success	Sustained success		1.2.1.2	S	0.125	S	R	R	S	S	S	S	S	136X	ERS28168329
040	033	PN-22-3423	Cured	Sustained success	Sustained success		4.3.4.1	S	0.25	S	R	R	S	R	R	R	S	129X	ERS28168330
015	036	PN-21-3317	Cured	Sustained success	Sustained success		1.2.1.2.1	R	1	S	R	R	R	S	S	S	S	74X	ERS28168331
075	038	PN-22-7031	Cured	Recurrence	Recurrence		4.3.4.1	R	0.25	S	R	R	S	R	S	S	S	128X	ERS28168332
123	039	PN-22-10942	Cured	Sustained success	Sustained success		4.3.4.2	S	0.125	S	R	R	R	R	S	S	S	72X	ERS28168333
034	041	PN-22-2841	Cured	Sustained success	Sustained success		1.2.1.2.1	S	1	S	R	R	ND	ND	S	S	S	263X	ERS28168334
106	045	PN-23-02401	Cured	Sustained success	Sustained success		4.3.4.1	S	0.125	S	S	S	ND	ND	R	R	S	84X	ERS28168335
134	047	PN-23-00513	Cured	Sustained success	Sustained success		1.2.1.2.1	S	2	S	R	R	S	S	S	S	S	53X	ERS28168336
091	048	PN-23-00617	Cured	NTM	Sustained success		1.2.1.2.1	S	2	S	R	R	ND	ND	R	R	S	79X	ERS28168337
130	049	PN-23-01711	Cured	Sustained success	Sustained success		4.3.4.1	S	0.125	S	R	S	S	S	S	S	S	154X	ERS28168338
111	053	PN-23-00454	Cured	Sustained success	Culture ND	Failed to come for 12M FU	1.2.1.2.1	S	2	S	ND	ND	ND	ND	ND	ND	ND	165X	ERS28168339
112	054	PN-23-00216	Cured	Sustained success	Sustained success		1.2.1.2	S	2	S	S	S	S	S	S	S	S	271X	ERS28168340
113	055	PN-23-00217	Cured	Sustained success	Sustained success		1.2.1.2.1	S	2	S	R	R	S	S	S	S	S	200X	ERS28168341
114	056	PN-23-00218	Cured	Sustained success	Sustained success		1.2.1.2.1	S	1	S	S	S	S	S	S	S	S	96X	ERS28168342
133	059	PN-23-02904	Cured	Sustained success	Sustained success		1.2.1.2.1	S	4	S	ND	ND	ND	ND	ND	ND	ND	83X	ERS28168343

Abbreviations:

ID	identifier
WGS	whole genome sequence
EoT	end of treatment
FU	follow-up period
6M	6 months
12M	12 months
DST	drug susceptibility testing
MIC	minimum inhibitory concentration
ND	not done
NTM	non-tuberculous mycoacteria
BDQ	bedaquline
PMD	pretomanid
LZD	linezolid
INH	isoniazid
RIF	rifampicin
EMB	ethambutol
SM	streptomycin
MFX	moxifloxacin
LFX	levoflxacin

AM amikacin

Supplementary Table 2: Gene variants identified by TB-profiler

sample	gene_name	change	freq	type	drugs
BPAL-002	gyrA	p.Asp94Gly	0.6	missense_variant	levofloxacin;moxifloxacin
BPAL-002	rpoB	p.Ser450Leu	1.0	missense_variant	rifampicin
BPAL-002	rpoB	p.Arg552Cys	0.388888888888888	missense_variant	rifampicin
BPAL-002	inhA	c.-777C>T	1.0	upstream_gene_var	ethionamide;isoniazid
BPAL-002	gyrA	p.Glu21Gln	1.0	missense_variant	-
BPAL-002	gyrA	p.Ser95Thr	1.0	missense_variant	-
BPAL-002	gyrA	p.Gly668Asp	1.0	missense_variant	-
BPAL-002	fgd1	p.Lys270Met	1.0	missense_variant	-
BPAL-002	mshA	p.Asn111Ser	1.0	missense_variant	-
BPAL-002	Rv0565c	p.Ser68Pro	1.0	missense_variant	-
BPAL-002	rpoB	c.309C>T	1.0	synonymous_variar	-
BPAL-002	rpoB	p.Ile488Val	0.121951219512195	missense_variant	-
BPAL-002	rpoB	p.Thr928Asn	0.162790697674418	missense_variant	-
BPAL-002	rpoC	p.Asp485Tyr	0.333333333333333	missense_variant	-
BPAL-002	rpoC	p.Gly594Glu	1.0	missense_variant	-
BPAL-002	mmpL5	p.Ile948Val	1.0	missense_variant	-
BPAL-002	rpsL	c.-165T>C	1.0	upstream_gene_var	-
BPAL-002	rrs	n.-187C>T	1.0	upstream_gene_var	-
BPAL-002	inhA	p.Ile95Leu	1.0	missense_variant	-
BPAL-002	tsnR	p.Leu232Pro	1.0	missense_variant	-
BPAL-002	tlyA	c.33A>G	1.0	synonymous_variar	-
BPAL-002	Rv1979c	c.-129A>G	1.0	upstream_gene_var	-
BPAL-002	kasA	c.-39C>T	1.0	upstream_gene_var	-
BPAL-002	Rv2680	c.-71G>A	0.2	upstream_gene_var	-
BPAL-002	Rv2680	c.-20G>A	0.244897959183673	upstream_gene_var	-
BPAL-002	ald	c.-32T>C	1.0	upstream_gene_var	-
BPAL-002	mtrB	p.Met517Leu	1.0	missense_variant	-
BPAL-002	embC	c.-565C>T	1.0	upstream_gene_var	-
BPAL-002	embC	c.2781C>T	1.0	synonymous_variar	-
BPAL-002	embC	p.Val981Leu	1.0	missense_variant	-
BPAL-002	whiB6	c.-75delG	1.0	upstream_gene_var	-
BPAL-002	whiB6	c.-211C>T	1.0	upstream_gene_var	-
BPAL-002	gid	p.Gly71Arg	1.0	missense_variant	-
BPAL-003	rpoB	p.Ser450Leu	1.0	missense_variant	rifampicin
BPAL-003	rpoC	p.Ile491Thr	1.0	missense_variant	rifampicin
BPAL-003	inhA	c.-777C>T	1.0	upstream_gene_var	ethionamide;isoniazid
BPAL-003	gyrA	p.Glu21Gln	1.0	missense_variant	-
BPAL-003	gyrA	p.Ser95Thr	1.0	missense_variant	-
BPAL-003	gyrA	p.Gly668Asp	1.0	missense_variant	-
BPAL-003	rpoC	c.1626C>G	1.0	synonymous_variar	-
BPAL-003	mmpL5	p.Ile948Val	1.0	missense_variant	-
BPAL-003	rpsL	c.-165T>C	1.0	upstream_gene_var	-
BPAL-003	embR	p.Phe376Leu	0.409090909090909	missense_variant	-
BPAL-003	embR	p.Cys372Gly	0.4	missense_variant	-
BPAL-003	rrs	n.-187C>T	1.0	upstream_gene_var	-
BPAL-003	tsnR	p.Leu232Pro	1.0	missense_variant	-
BPAL-003	tlyA	c.33A>G	1.0	synonymous_variar	-
BPAL-003	katG	p.Asp714Glu	0.2	missense_variant	-
BPAL-003	katG	p.Ala110Thr	1.0	missense_variant	-
BPAL-003	Rv1979c	c.-129A>G	1.0	upstream_gene_var	-
BPAL-003	Rv2752c	p.Val300Ala	1.0	missense_variant	-
BPAL-003	thyA	p.Thr202Ala	1.0	missense_variant	-
BPAL-003	ald	c.-32T>C	1.0	upstream_gene_var	-
BPAL-003	Rv3236c	p.Ala370Thr	1.0	missense_variant	-
BPAL-003	mtrB	p.Met517Leu	1.0	missense_variant	-

BPAL-003	clpC1	c.2418C>T	1.0	synonymous_variar	-
BPAL-003	embC	c.-100C>T	1.0	upstream_gene_var	-
BPAL-003	embC	c.2781C>T	1.0	synonymous_variar	-
BPAL-003	embA	p.Ile821Thr	1.0	missense_variant	-
BPAL-003	whiB6	c.-75delG	1.0	upstream_gene_var	-
BPAL-003	whiB6	c.-211C>T	1.0	upstream_gene_var	-
BPAL-003	gid	p.Asn51Tyr	1.0	missense_variant	-
BPAL-003	gid	p.Leu16Arg	1.0	missense_variant	-
BPAL-005	gyrA	p.Asp94Ala	1.0	missense_variant	levofloxacin;moxifloxacin
BPAL-005	rpoB	p.Ser450Leu	1.0	missense_variant	rifampicin
BPAL-005	katG	p.Ser315Thr	1.0	missense_variant	isoniazid
BPAL-005	gid	c.222delG	1.0	frameshift_variant	streptomycin
BPAL-005	gyrA	p.Glu21Gln	1.0	missense_variant	-
BPAL-005	gyrA	p.Ser95Thr	1.0	missense_variant	-
BPAL-005	gyrA	p.Gly668Asp	1.0	missense_variant	-
BPAL-005	Rv0010c	p.Ile25Met	0.941176470588235	missense_variant	-
BPAL-005	fgd1	c.960T>C	1.0	synonymous_variar	-
BPAL-005	mshA	p.Ala187Val	1.0	missense_variant	-
BPAL-005	ccsA	p.Ile245Met	1.0	missense_variant	-
BPAL-005	Rv0565c	c.390G>A	1.0	synonymous_variar	-
BPAL-005	Rv0565c	p.Arg110His	1.0	missense_variant	-
BPAL-005	rpoB	c.3225T>C	1.0	synonymous_variar	-
BPAL-005	rpoC	p.Val483Gly	1.0	missense_variant	-
BPAL-005	mmpL5	p.Ile948Val	1.0	missense_variant	-
BPAL-005	mmpL5	p.Thr794Ile	1.0	missense_variant	-
BPAL-005	mmpL5	p.Asp767Asn	1.0	missense_variant	-
BPAL-005	mmpS5	c.-710C>G	1.0	upstream_gene_var	-
BPAL-005	rpsL	c.-165T>C	1.0	upstream_gene_var	-
BPAL-005	Rv1129c	c.-28T>C	1.0	upstream_gene_var	-
BPAL-005	Rv1258c	c.580_581ins	1.0	frameshift_variant	-
BPAL-005	embR	p.Phe376Leu	0.131147540983606	missense_variant	-
BPAL-005	embR	p.Cys372Gly	0.126984126984126	missense_variant	-
BPAL-005	embR	p.Val50Ala	1.0	missense_variant	-
BPAL-005	rrs	n.-187C>T	1.0	upstream_gene_var	-
BPAL-005	rpsA	c.636A>C	1.0	synonymous_variar	-
BPAL-005	tsnR	p.Leu232Pro	1.0	missense_variant	-
BPAL-005	tlyA	c.33A>G	1.0	synonymous_variar	-
BPAL-005	katG	p.Arg463Leu	1.0	missense_variant	-
BPAL-005	PPE35	p.Leu896Ser	1.0	missense_variant	-
BPAL-005	PPE35	c.1497T>G	1.0	synonymous_variar	-
BPAL-005	Rv1979c	c.-129A>G	1.0	upstream_gene_var	-
BPAL-005	ald	c.-32T>C	1.0	upstream_gene_var	-
BPAL-005	Rv3236c	p.Val293Ala	1.0	missense_variant	-
BPAL-005	Rv3236c	p.Thr102Ala	1.0	missense_variant	-
BPAL-005	mtrB	p.Met517Leu	1.0	missense_variant	-
BPAL-005	mtrB	p.Pro18Ser	1.0	missense_variant	-
BPAL-005	embC	c.2781C>T	1.0	synonymous_variar	-
BPAL-005	embA	c.228C>T	1.0	synonymous_variar	-
BPAL-005	aftB	p.Asp397Gly	1.0	missense_variant	-
BPAL-005	whiB6	c.-75delG	1.0	upstream_gene_var	-
BPAL-005	whiB6	c.-211C>T	1.0	upstream_gene_var	-
BPAL-005	gid	c.615A>G	1.0	synonymous_variar	-
BPAL-005	gid	p.Glu92Asp	1.0	missense_variant	-
BPAL-006	gyrA	p.Ala90Val	0.703703703703703	missense_variant	levofloxacin;moxifloxacin
BPAL-006	rpoB	p.Ser450Leu	1.0	missense_variant	rifampicin
BPAL-006	rrs	n.514A>C	1.0	non_coding_transcr	streptomycin
BPAL-006	inhA	c.-777C>T	1.0	upstream_gene_var	ethionamide;isoniazid
BPAL-006	dnaA	c.1302C>A	1.0	synonymous_variar	-
BPAL-006	gyrB	p.Met291Ile	1.0	missense_variant	-

BPAL-006	gyrA	p.Glu21Gln	1.0	missense_variant	-
BPAL-006	gyrA	p.Ser95Thr	1.0	missense_variant	-
BPAL-006	gyrA	p.Ala384Val	1.0	missense_variant	-
BPAL-006	gyrA	c.1842T>C	1.0	synonymous_variar	-
BPAL-006	gyrA	c.1959G>C	1.0	synonymous_variar	-
BPAL-006	gyrA	p.Gly668Asp	1.0	missense_variant	-
BPAL-006	Rv0010c	c.384T>C	1.0	synonymous_variar	-
BPAL-006	Rv0010c	p.Ile87Met	1.0	missense_variant	-
BPAL-006	Rv0010c	c.99T>C	1.0	synonymous_variar	-
BPAL-006	fgd1	c.960T>C	1.0	synonymous_variar	-
BPAL-006	mshA	c.21T>C	0.961538461538461	synonymous_variar	-
BPAL-006	Rv0565c	c.-108T>C	1.0	upstream_gene_var	-
BPAL-006	nusG	c.-138T>C	1.0	upstream_gene_var	-
BPAL-006	rpoB	c.3225T>C	1.0	synonymous_variar	-
BPAL-006	rpoC	c.162G>C	1.0	synonymous_variar	-
BPAL-006	rpoC	p.Ala172Val	1.0	missense_variant	-
BPAL-006	rpoC	c.517C>A	1.0	synonymous_variar	-
BPAL-006	rpoC	p.Leu516Pro	1.0	missense_variant	-
BPAL-006	mmpL5	p.Ile948Val	1.0	missense_variant	-
BPAL-006	mmpL5	p.Thr794Ile	1.0	missense_variant	-
BPAL-006	rpsL	c.-165T>C	1.0	upstream_gene_var	-
BPAL-006	rplC	c.-452C>A	1.0	upstream_gene_var	-
BPAL-006	Rv1129c	c.-28T>C	1.0	upstream_gene_var	-
BPAL-006	Rv1258c	c.1029T>C	1.0	synonymous_variar	-
BPAL-006	embR	p.Phe376Leu	0.268292682926829	missense_variant	-
BPAL-006	embR	p.Cys372Gly	0.238095238095238	missense_variant	-
BPAL-006	embR	p.Cys110Tyr	1.0	missense_variant	-
BPAL-006	embR	c.-207C>G	1.0	upstream_gene_var	-
BPAL-006	embR	c.-446C>T	1.0	upstream_gene_var	-
BPAL-006	atpE	c.-138T>C	1.0	upstream_gene_var	-
BPAL-006	rrs	n.-187C>T	1.0	upstream_gene_var	-
BPAL-006	rrl	n.982G>A	1.0	non_coding_transcr	-
BPAL-006	inhA	c.-40C>T	1.0	upstream_gene_var	-
BPAL-006	tsnR	c.369C>T	1.0	synonymous_variar	-
BPAL-006	tsnR	p.Leu232Pro	1.0	missense_variant	-
BPAL-006	tlyA	c.33A>G	1.0	synonymous_variar	-
BPAL-006	tlyA	p.Val32Leu	1.0	missense_variant	-
BPAL-006	bacA	p.Ile603Val	1.0	missense_variant	-
BPAL-006	ndh	p.Tyr403Cys	1.0	missense_variant	-
BPAL-006	katG	p.Arg463Leu	1.0	missense_variant	-
BPAL-006	PPE35	p.Leu896Ser	1.0	missense_variant	-
BPAL-006	Rv1979c	p.Asp286Gly	1.0	missense_variant	-
BPAL-006	Rv1979c	c.-129A>G	1.0	upstream_gene_var	-
BPAL-006	kasA	c.18C>T	1.0	synonymous_variar	-
BPAL-006	kasA	p.Gly312Ser	1.0	missense_variant	-
BPAL-006	ahpC	c.-142G>A	1.0	upstream_gene_var	-
BPAL-006	ald	c.-32T>C	1.0	upstream_gene_var	-
BPAL-006	fbiD	c.300A>G	1.0	synonymous_variar	-
BPAL-006	Rv3083	p.Asp71His	1.0	missense_variant	-
BPAL-006	whiB7	c.188delG	1.0	frameshift_variant	-
BPAL-006	lpqB	p.Asp142Gly	1.0	missense_variant	-
BPAL-006	mtrB	p.Met517Leu	1.0	missense_variant	-
BPAL-006	fbiA	c.15T>C	1.0	synonymous_variar	-
BPAL-006	clpC1	p.Val63Ala	1.0	missense_variant	-
BPAL-006	glpK	p.Val460Ala	1.0	missense_variant	-
BPAL-006	embC	p.Thr270Ile	1.0	missense_variant	-
BPAL-006	embC	p.Asn394Asp	1.0	missense_variant	-
BPAL-006	embC	c.2781C>T	1.0	synonymous_variar	-
BPAL-006	embA	c.348G>A	1.0	synonymous_variar	-

BPAL-006	embA	c.1188G>C	1.0	synonymous_variar	-
BPAL-006	embA	p.Pro913Ser	1.0	missense_variant	-
BPAL-006	embB	c.1065G>A	1.0	synonymous_variar	-
BPAL-006	embB	p.Glu378Ala	1.0	missense_variant	-
BPAL-006	ubiA	p.Glu149Asp	1.0	missense_variant	-
BPAL-006	ubiA	c.228T>C	1.0	synonymous_variar	-
BPAL-006	ubiA	c.-36delG	1.0	upstream_gene_var	-
BPAL-006	whiB6	p.Arg54Gln	1.0	missense_variant	-
BPAL-006	whiB6	c.-75delG	1.0	upstream_gene_var	-
BPAL-006	whiB6	c.-82C>T	1.0	upstream_gene_var	-
BPAL-006	whiB6	c.-211C>T	1.0	upstream_gene_var	-
BPAL-006	gid	c.615A>G	1.0	synonymous_variar	-
BPAL-006	gid	p.Arg176His	1.0	missense_variant	-
BPAL-006	gid	c.330G>T	1.0	synonymous_variar	-
BPAL-006	gid	p.Cys52Trp	1.0	missense_variant	-
BPAL-007	gyrA	p.Asp94Ala	1.0	missense_variant	levofloxacin;moxifloxacin
BPAL-007	rpoB	p.Ser450Leu	1.0	missense_variant	rifampicin
BPAL-007	katG	p.Ser315Thr	1.0	missense_variant	isoniazid
BPAL-007	gid	c.222delG	1.0	frameshift_variant	streptomycin
BPAL-007	gyrA	p.Glu21Gln	1.0	missense_variant	-
BPAL-007	gyrA	p.Ser95Thr	1.0	missense_variant	-
BPAL-007	gyrA	p.Gly668Asp	1.0	missense_variant	-
BPAL-007	Rv0010c	p.Ile25Met	1.0	missense_variant	-
BPAL-007	fgd1	c.960T>C	1.0	synonymous_variar	-
BPAL-007	mshA	p.Ala187Val	0.981132075471698	missense_variant	-
BPAL-007	ccsA	p.Ile245Met	1.0	missense_variant	-
BPAL-007	Rv0565c	c.390G>A	1.0	synonymous_variar	-
BPAL-007	Rv0565c	p.Arg110His	1.0	missense_variant	-
BPAL-007	rpoB	c.3225T>C	1.0	synonymous_variar	-
BPAL-007	mmpL5	p.Ile948Val	1.0	missense_variant	-
BPAL-007	mmpL5	p.Thr794Ile	0.9	missense_variant	-
BPAL-007	mmpL5	p.Asp767Asn	1.0	missense_variant	-
BPAL-007	mmpS5	c.-710C>G	1.0	upstream_gene_var	-
BPAL-007	rpsL	c.-165T>C	1.0	upstream_gene_var	-
BPAL-007	Rv1129c	c.-28T>C	1.0	upstream_gene_var	-
BPAL-007	Rv1258c	c.580_581ins	1.0	frameshift_variant	-
BPAL-007	embR	p.Phe376Leu	0.363636363636363	missense_variant	-
BPAL-007	embR	p.Cys372Gly	0.382978723404255	missense_variant	-
BPAL-007	rrs	n.-187C>T	1.0	upstream_gene_var	-
BPAL-007	rpsA	c.636A>C	1.0	synonymous_variar	-
BPAL-007	tsnR	p.Leu232Pro	1.0	missense_variant	-
BPAL-007	tlyA	c.33A>G	1.0	synonymous_variar	-
BPAL-007	katG	p.Arg463Leu	1.0	missense_variant	-
BPAL-007	PPE35	p.Leu896Ser	1.0	missense_variant	-
BPAL-007	PPE35	c.1497T>G	1.0	synonymous_variar	-
BPAL-007	PPE35	p.Leu189Val	0.157894736842105	missense_variant	-
BPAL-007	PPE35	p.Thr187Ser	0.153846153846153	missense_variant	-
BPAL-007	Rv1979c	c.-129A>G	1.0	upstream_gene_var	-
BPAL-007	ald	c.-32T>C	1.0	upstream_gene_var	-
BPAL-007	Rv3236c	p.Val293Ala	1.0	missense_variant	-
BPAL-007	Rv3236c	p.Thr102Ala	1.0	missense_variant	-
BPAL-007	mtrB	p.Met517Leu	1.0	missense_variant	-
BPAL-007	mtrB	p.Pro18Ser	1.0	missense_variant	-
BPAL-007	embC	c.2781C>T	1.0	synonymous_variar	-
BPAL-007	embA	c.228C>T	1.0	synonymous_variar	-
BPAL-007	aftB	p.Asp397Gly	1.0	missense_variant	-
BPAL-007	whiB6	c.-75delG	1.0	upstream_gene_var	-
BPAL-007	whiB6	c.-211C>T	1.0	upstream_gene_var	-
BPAL-007	gid	c.615A>G	1.0	synonymous_variar	-

BPAL-007	gid	p.Glu92Asp	1.0	missense_variant	-
BPAL-009	rpoB	p.Ser450Leu	1.0	missense_variant	rifampicin
BPAL-009	katG	p.Ser315Thr	1.0	missense_variant	isoniazid
BPAL-009	embB	p.Gly406Asp	1.0	missense_variant	ethambutol
BPAL-009	gyrA	p.Glu21Gln	1.0	missense_variant	-
BPAL-009	gyrA	p.Ser95Thr	1.0	missense_variant	-
BPAL-009	gyrA	p.Gly668Asp	1.0	missense_variant	-
BPAL-009	Rv0010c	p.Thr40Pro	1.0	missense_variant	-
BPAL-009	rpoC	p.Glu96Asp	0.956521739130434	missense_variant	-
BPAL-009	rpoC	p.Val431Met	1.0	missense_variant	-
BPAL-009	rpoC	c.1626C>G	1.0	synonymous_variar	-
BPAL-009	mmpL5	p.Ile948Val	1.0	missense_variant	-
BPAL-009	rpsL	c.-165T>C	1.0	upstream_gene_var	-
BPAL-009	rrs	n.-187C>T	1.0	upstream_gene_var	-
BPAL-009	tsnR	p.Leu232Pro	1.0	missense_variant	-
BPAL-009	tlyA	c.33A>G	1.0	synonymous_variar	-
BPAL-009	Rv1979c	p.Pro390Arg	1.0	missense_variant	-
BPAL-009	Rv1979c	c.-129A>G	1.0	upstream_gene_var	-
BPAL-009	pncA	p.His51Leu	1.0	missense_variant	-
BPAL-009	Rv2752c	p.Val300Ala	1.0	missense_variant	-
BPAL-009	thyA	p.Thr202Ala	1.0	missense_variant	-
BPAL-009	ald	c.-32T>C	1.0	upstream_gene_var	-
BPAL-009	Rv3236c	p.Ala370Thr	1.0	missense_variant	-
BPAL-009	mtrB	p.Met517Leu	1.0	missense_variant	-
BPAL-009	fbiB	c.396G>A	1.0	synonymous_variar	-
BPAL-009	clpC1	c.2418C>T	1.0	synonymous_variar	-
BPAL-009	embC	c.-100C>T	1.0	upstream_gene_var	-
BPAL-009	embC	c.2781C>T	1.0	synonymous_variar	-
BPAL-009	whiB6	c.-75delG	1.0	upstream_gene_var	-
BPAL-009	whiB6	c.-211C>T	1.0	upstream_gene_var	-
BPAL-009	gid	p.Leu16Arg	1.0	missense_variant	-
BPAL-010	rpoB	p.Gln432Leu	1.0	missense_variant	rifampicin
BPAL-010	inhA	c.-154G>A	1.0	upstream_gene_var	ethionamide;isoniazid
BPAL-010	embB	p.Met306Leu	0.454545454545454	missense_variant	ethambutol
BPAL-010	gid	p.Gln125*	1.0	stop_gained	streptomycin
BPAL-010	dnaA	c.1302C>A	1.0	synonymous_variar	-
BPAL-010	gyrB	p.Met291Ile	1.0	missense_variant	-
BPAL-010	gyrA	p.Glu21Gln	1.0	missense_variant	-
BPAL-010	gyrA	p.Ser95Thr	1.0	missense_variant	-
BPAL-010	gyrA	p.Ala384Val	1.0	missense_variant	-
BPAL-010	gyrA	c.1842T>C	1.0	synonymous_variar	-
BPAL-010	gyrA	c.1959G>C	1.0	synonymous_variar	-
BPAL-010	gyrA	p.Gly668Asp	1.0	missense_variant	-
BPAL-010	Rv0010c	c.384T>C	1.0	synonymous_variar	-
BPAL-010	Rv0010c	p.Ile87Met	1.0	missense_variant	-
BPAL-010	Rv0010c	c.99T>C	1.0	synonymous_variar	-
BPAL-010	fgd1	c.960T>C	1.0	synonymous_variar	-
BPAL-010	mshA	c.21T>C	1.0	synonymous_variar	-
BPAL-010	Rv0565c	c.-108T>C	1.0	upstream_gene_var	-
BPAL-010	nusG	c.-138T>C	1.0	upstream_gene_var	-
BPAL-010	rpoB	c.3225T>C	1.0	synonymous_variar	-
BPAL-010	rpoC	c.162G>C	1.0	synonymous_variar	-
BPAL-010	rpoC	p.Ala172Val	1.0	missense_variant	-
BPAL-010	rpoC	c.517C>A	1.0	synonymous_variar	-
BPAL-010	mmpL5	p.Ile948Val	1.0	missense_variant	-
BPAL-010	mmpL5	p.Thr794Ile	1.0	missense_variant	-
BPAL-010	rpsL	c.-165T>C	1.0	upstream_gene_var	-
BPAL-010	rplC	c.-452C>A	1.0	upstream_gene_var	-
BPAL-010	Rv1129c	c.-28T>C	1.0	upstream_gene_var	-

BPAL-010	Rv1258c	c.1029T>C	1.0	synonymous_variar	-
BPAL-010	embR	p.Phe376Leu	0.163636363636363	missense_variant	-
BPAL-010	embR	p.Cys372Gly	0.157894736842105	missense_variant	-
BPAL-010	embR	p.Cys110Tyr	1.0	missense_variant	-
BPAL-010	embR	c.-207C>G	1.0	upstream_gene_var	-
BPAL-010	embR	c.-446C>T	1.0	upstream_gene_var	-
BPAL-010	atpE	c.-138T>C	1.0	upstream_gene_var	-
BPAL-010	rrs	n.-187C>T	1.0	upstream_gene_var	-
BPAL-010	rrl	n.982G>A	1.0	non_coding_transcı	-
BPAL-010	inhA	c.-40C>T	1.0	upstream_gene_var	-
BPAL-010	inhA	p.Ala190Ser	1.0	missense_variant	-
BPAL-010	tsnR	c.369C>T	1.0	synonymous_variar	-
BPAL-010	tsnR	p.Leu232Pro	1.0	missense_variant	-
BPAL-010	tlyA	c.33A>G	1.0	synonymous_variar	-
BPAL-010	bacA	p.Ile603Val	1.0	missense_variant	-
BPAL-010	katG	p.Arg463Leu	1.0	missense_variant	-
BPAL-010	PPE35	p.Leu896Ser	1.0	missense_variant	-
BPAL-010	Rv1979c	p.Asp286Gly	1.0	missense_variant	-
BPAL-010	Rv1979c	c.-129A>G	1.0	upstream_gene_var	-
BPAL-010	kasA	c.18C>T	1.0	synonymous_variar	-
BPAL-010	kasA	p.Gly312Ser	1.0	missense_variant	-
BPAL-010	ahpC	c.-142G>A	1.0	upstream_gene_var	-
BPAL-010	Rv2752c	c.804C>T	1.0	synonymous_variar	-
BPAL-010	ald	c.-32T>C	1.0	upstream_gene_var	-
BPAL-010	fbiD	c.300A>G	1.0	synonymous_variar	-
BPAL-010	Rv3083	p.Asp71His	1.0	missense_variant	-
BPAL-010	whiB7	c.188delG	1.0	frameshift_variant	-
BPAL-010	lpqB	p.Asp142Gly	1.0	missense_variant	-
BPAL-010	mtrB	p.Met517Leu	1.0	missense_variant	-
BPAL-010	fbiA	c.15T>C	1.0	synonymous_variar	-
BPAL-010	clpC1	p.Val63Ala	1.0	missense_variant	-
BPAL-010	glpK	p.Val460Ala	1.0	missense_variant	-
BPAL-010	glpK	p.Arg386Gln	1.0	missense_variant	-
BPAL-010	embC	p.Thr270Ile	1.0	missense_variant	-
BPAL-010	embC	p.Asn394Asp	1.0	missense_variant	-
BPAL-010	embC	c.2781C>T	1.0	synonymous_variar	-
BPAL-010	embA	c.348G>A	1.0	synonymous_variar	-
BPAL-010	embA	c.1188G>C	1.0	synonymous_variar	-
BPAL-010	embA	p.Pro913Ser	1.0	missense_variant	-
BPAL-010	embB	c.1065G>A	1.0	synonymous_variar	-
BPAL-010	embB	p.Glu378Ala	1.0	missense_variant	-
BPAL-010	ubiA	p.Glu149Asp	1.0	missense_variant	-
BPAL-010	ubiA	c.228T>C	1.0	synonymous_variar	-
BPAL-010	ubiA	c.-36delG	1.0	upstream_gene_var	-
BPAL-010	whiB6	p.Arg54Gln	1.0	missense_variant	-
BPAL-010	whiB6	c.-75delG	1.0	upstream_gene_var	-
BPAL-010	whiB6	c.-82C>T	1.0	upstream_gene_var	-
BPAL-010	whiB6	c.-211C>T	1.0	upstream_gene_var	-
BPAL-010	gid	c.615A>G	1.0	synonymous_variar	-
BPAL-010	gid	c.330G>T	1.0	synonymous_variar	-
BPAL-013	rpoB	p.Ser450Trp	1.0	missense_variant	rifampicin
BPAL-013	inhA	c.-777C>T	1.0	upstream_gene_var	ethionamide;isoniazid
BPAL-013	inhA	p.Ile21Val	1.0	missense_variant	isoniazid
BPAL-013	dnaA	c.1302C>A	1.0	synonymous_variar	-
BPAL-013	gyrB	p.Met291Ile	1.0	missense_variant	-
BPAL-013	gyrA	p.Glu21Gln	1.0	missense_variant	-
BPAL-013	gyrA	p.Ser95Thr	1.0	missense_variant	-
BPAL-013	gyrA	p.Ala384Val	1.0	missense_variant	-
BPAL-013	gyrA	p.Arg607Cys	1.0	missense_variant	-

BPAL-013	gyrA	c.1842T>C	1.0	synonymous_variar	-
BPAL-013	gyrA	c.1959G>C	1.0	synonymous_variar	-
BPAL-013	gyrA	p.Gly668Asp	1.0	missense_variant	-
BPAL-013	Rv0010c	c.384T>C	1.0	synonymous_variar	-
BPAL-013	Rv0010c	p.Ile87Met	1.0	missense_variant	-
BPAL-013	Rv0010c	c.99T>C	1.0	synonymous_variar	-
BPAL-013	fgd1	c.960T>C	1.0	synonymous_variar	-
BPAL-013	mshA	c.21T>C	1.0	synonymous_variar	-
BPAL-013	Rv0565c	c.-108T>C	1.0	upstream_gene_var	-
BPAL-013	nusG	c.-138T>C	1.0	upstream_gene_var	-
BPAL-013	rpoB	c.3225T>C	1.0	synonymous_variar	-
BPAL-013	rpoC	c.162G>C	1.0	synonymous_variar	-
BPAL-013	rpoC	p.Ala172Val	1.0	missense_variant	-
BPAL-013	rpoC	c.517C>A	1.0	synonymous_variar	-
BPAL-013	rpoC	c.1440C>T	1.0	synonymous_variar	-
BPAL-013	rpoC	p.Asn698Ser	1.0	missense_variant	-
BPAL-013	mmpL5	p.Ile948Val	1.0	missense_variant	-
BPAL-013	mmpL5	p.Thr794Ile	1.0	missense_variant	-
BPAL-013	rpsL	c.-165T>C	1.0	upstream_gene_var	-
BPAL-013	rplC	c.-452C>A	1.0	upstream_gene_var	-
BPAL-013	Rv1129c	c.-28T>C	1.0	upstream_gene_var	-
BPAL-013	fbiC	c.-110G>A	1.0	upstream_gene_var	-
BPAL-013	Rv1258c	c.1029T>C	1.0	synonymous_variar	-
BPAL-013	embR	p.Cys110Tyr	1.0	missense_variant	-
BPAL-013	embR	c.-207C>G	1.0	upstream_gene_var	-
BPAL-013	embR	c.-446C>T	1.0	upstream_gene_var	-
BPAL-013	atpE	c.-138T>C	1.0	upstream_gene_var	-
BPAL-013	rrs	n.-187C>T	1.0	upstream_gene_var	-
BPAL-013	inhA	c.-40C>T	1.0	upstream_gene_var	-
BPAL-013	tsnR	c.369C>T	1.0	synonymous_variar	-
BPAL-013	tsnR	p.Leu232Pro	1.0	missense_variant	-
BPAL-013	tlyA	c.33A>G	1.0	synonymous_variar	-
BPAL-013	bacA	p.Ile603Val	1.0	missense_variant	-
BPAL-013	bacA	p.Ser212Ile	0.130434782608695	missense_variant	-
BPAL-013	katG	p.Arg463Leu	1.0	missense_variant	-
BPAL-013	PPE35	p.Leu896Ser	1.0	missense_variant	-
BPAL-013	Rv1979c	p.Asp286Gly	1.0	missense_variant	-
BPAL-013	Rv1979c	c.-129A>G	1.0	upstream_gene_var	-
BPAL-013	kasA	c.18C>T	1.0	synonymous_variar	-
BPAL-013	kasA	p.Gly312Ser	1.0	missense_variant	-
BPAL-013	ahpC	c.-142G>A	1.0	upstream_gene_var	-
BPAL-013	ald	c.-32T>C	1.0	upstream_gene_var	-
BPAL-013	fbiD	c.300A>G	1.0	synonymous_variar	-
BPAL-013	Rv3083	p.Asp71His	1.0	missense_variant	-
BPAL-013	whiB7	c.188delG	1.0	frameshift_variant	-
BPAL-013	lpqB	p.Asp142Gly	1.0	missense_variant	-
BPAL-013	mtrB	p.Met517Leu	1.0	missense_variant	-
BPAL-013	mtrA	p.Thr3Ala	1.0	missense_variant	-
BPAL-013	fbiA	c.-290T>C	1.0	upstream_gene_var	-
BPAL-013	fbiA	c.15T>C	1.0	synonymous_variar	-
BPAL-013	clpC1	p.Val63Ala	1.0	missense_variant	-
BPAL-013	glpK	p.Val460Ala	1.0	missense_variant	-
BPAL-013	embC	p.Thr270Ile	1.0	missense_variant	-
BPAL-013	embC	p.Asn394Asp	1.0	missense_variant	-
BPAL-013	embC	c.2781C>T	1.0	synonymous_variar	-
BPAL-013	embA	c.348G>A	1.0	synonymous_variar	-
BPAL-013	embA	c.1188G>C	1.0	synonymous_variar	-
BPAL-013	embA	p.Pro913Ser	1.0	missense_variant	-
BPAL-013	embB	c.54_55insT	0.206896551724137	frameshift_variant	-

BPAL-013	embB	c.1065G>A	1.0	synonymous_variar	-
BPAL-013	embB	p.Glu378Ala	1.0	missense_variant	-
BPAL-013	ubiA	p.Glu149Asp	1.0	missense_variant	-
BPAL-013	ubiA	c.228T>C	1.0	synonymous_variar	-
BPAL-013	ubiA	c.-36delG	1.0	upstream_gene_var	-
BPAL-013	whiB6	p.Arg54Gln	1.0	missense_variant	-
BPAL-013	whiB6	c.-75delG	1.0	upstream_gene_var	-
BPAL-013	whiB6	c.-82C>T	1.0	upstream_gene_var	-
BPAL-013	whiB6	c.-211C>T	1.0	upstream_gene_var	-
BPAL-013	gid	c.615A>G	1.0	synonymous_variar	-
BPAL-013	gid	c.330G>T	1.0	synonymous_variar	-
BPAL-013	gid	p.Val66Glu	1.0	missense_variant	-
BPAL-015	rpoB	p.Ser450Leu	1.0	missense_variant	rifampicin
BPAL-015	katG	p.Ser315Thr	1.0	missense_variant	isoniazid
BPAL-015	pncA	c.185_186ins	1.0	frameshift_variant	pyrazinamide
BPAL-015	embB	p.Gln497Arg	1.0	missense_variant	ethambutol
BPAL-015	gid	c.98delG	1.0	frameshift_variant	streptomycin
BPAL-015	dnaA	c.1302C>A	1.0	synonymous_variar	-
BPAL-015	gyrB	p.Met291Ile	1.0	missense_variant	-
BPAL-015	gyrA	p.Glu21Gln	1.0	missense_variant	-
BPAL-015	gyrA	p.Ser95Thr	1.0	missense_variant	-
BPAL-015	gyrA	p.Ala384Val	1.0	missense_variant	-
BPAL-015	gyrA	c.1842T>C	1.0	synonymous_variar	-
BPAL-015	gyrA	c.1959G>C	1.0	synonymous_variar	-
BPAL-015	gyrA	p.Gly668Asp	1.0	missense_variant	-
BPAL-015	Rv0010c	c.384T>C	1.0	synonymous_variar	-
BPAL-015	Rv0010c	p.Ile87Met	1.0	missense_variant	-
BPAL-015	Rv0010c	c.99T>C	1.0	synonymous_variar	-
BPAL-015	fgd1	c.960T>C	1.0	synonymous_variar	-
BPAL-015	mshA	c.21T>C	1.0	synonymous_variar	-
BPAL-015	Rv0565c	c.-108T>C	1.0	upstream_gene_var	-
BPAL-015	nusG	c.-138T>C	1.0	upstream_gene_var	-
BPAL-015	rpoB	p.Ile873Phe	1.0	missense_variant	-
BPAL-015	rpoB	c.3225T>C	1.0	synonymous_variar	-
BPAL-015	rpoC	c.162G>C	1.0	synonymous_variar	-
BPAL-015	rpoC	p.Ala172Val	1.0	missense_variant	-
BPAL-015	rpoC	c.517C>A	1.0	synonymous_variar	-
BPAL-015	mmpL5	p.Ile948Val	1.0	missense_variant	-
BPAL-015	mmpL5	p.Thr794Ile	1.0	missense_variant	-
BPAL-015	rpsL	c.-165T>C	1.0	upstream_gene_var	-
BPAL-015	rplC	c.-452C>A	1.0	upstream_gene_var	-
BPAL-015	Rv1129c	c.-28T>C	1.0	upstream_gene_var	-
BPAL-015	Rv1258c	c.1029T>C	1.0	synonymous_variar	-
BPAL-015	embR	p.Cys110Tyr	1.0	missense_variant	-
BPAL-015	embR	c.-207C>G	1.0	upstream_gene_var	-
BPAL-015	embR	c.-446C>T	1.0	upstream_gene_var	-
BPAL-015	atpE	c.-138T>C	1.0	upstream_gene_var	-
BPAL-015	rrs	n.-187C>T	1.0	upstream_gene_var	-
BPAL-015	rrl	n.982G>A	1.0	non_coding_transcı	-
BPAL-015	inhA	c.-40C>T	1.0	upstream_gene_var	-
BPAL-015	tsnR	c.369C>T	1.0	synonymous_variar	-
BPAL-015	tsnR	p.Leu232Pro	1.0	missense_variant	-
BPAL-015	tlyA	c.33A>G	1.0	synonymous_variar	-
BPAL-015	tlyA	p.Val32Leu	1.0	missense_variant	-
BPAL-015	bacA	p.Ile603Val	1.0	missense_variant	-
BPAL-015	katG	p.Arg463Leu	1.0	missense_variant	-
BPAL-015	PPE35	p.Leu896Ser	1.0	missense_variant	-
BPAL-015	Rv1979c	p.Asp286Gly	1.0	missense_variant	-
BPAL-015	Rv1979c	c.-129A>G	1.0	upstream_gene_var	-

BPAL-015	kasA	c.18C>T	0.964285714285714	synonymous_variar	-
BPAL-015	kasA	p.Gly312Ser	1.0	missense_variant	-
BPAL-015	ahpC	c.-142G>A	1.0	upstream_gene_var	-
BPAL-015	ald	c.-32T>C	1.0	upstream_gene_var	-
BPAL-015	fbiD	c.300A>G	0.966666666666666	synonymous_variar	-
BPAL-015	Rv3083	p.Asp71His	1.0	missense_variant	-
BPAL-015	lpqB	p.Asp142Gly	1.0	missense_variant	-
BPAL-015	mtrB	p.Met517Leu	1.0	missense_variant	-
BPAL-015	fbiA	c.15T>C	1.0	synonymous_variar	-
BPAL-015	clpC1	p.Val63Ala	1.0	missense_variant	-
BPAL-015	glpK	p.Val460Ala	1.0	missense_variant	-
BPAL-015	embC	p.Thr270Ile	1.0	missense_variant	-
BPAL-015	embC	p.Asn394Asp	1.0	missense_variant	-
BPAL-015	embC	c.2781C>T	1.0	synonymous_variar	-
BPAL-015	embA	c.348G>A	1.0	synonymous_variar	-
BPAL-015	embA	c.1188G>C	1.0	synonymous_variar	-
BPAL-015	embA	p.Pro913Ser	1.0	missense_variant	-
BPAL-015	embB	c.1065G>A	1.0	synonymous_variar	-
BPAL-015	embB	p.Glu378Ala	1.0	missense_variant	-
BPAL-015	ubiA	p.Glu149Asp	1.0	missense_variant	-
BPAL-015	ubiA	c.228T>C	1.0	synonymous_variar	-
BPAL-015	ubiA	c.-36delG	1.0	upstream_gene_var	-
BPAL-015	whiB6	p.Arg54Gln	1.0	missense_variant	-
BPAL-015	whiB6	c.-75delG	1.0	upstream_gene_var	-
BPAL-015	whiB6	c.-82C>T	1.0	upstream_gene_var	-
BPAL-015	whiB6	c.-211C>T	1.0	upstream_gene_var	-
BPAL-015	gid	c.615A>G	1.0	synonymous_variar	-
BPAL-015	gid	c.330G>T	1.0	synonymous_variar	-
BPAL-017	rpoB	p.Ser450Leu	1.0	missense_variant	rifampicin
BPAL-017	inhA	c.-777C>T	1.0	upstream_gene_var	ethionamide;isoniazid
BPAL-017	inhA	p.Ile194Thr	1.0	missense_variant	ethionamide;isoniazid
BPAL-017	embA	c.-12C>T	1.0	upstream_gene_var	ethambutol
BPAL-017	dnaA	c.1302C>A	1.0	synonymous_variar	-
BPAL-017	gyrB	p.Met291Ile	1.0	missense_variant	-
BPAL-017	gyrA	p.Glu21Gln	1.0	missense_variant	-
BPAL-017	gyrA	p.Ser95Thr	1.0	missense_variant	-
BPAL-017	gyrA	p.Ala384Val	1.0	missense_variant	-
BPAL-017	gyrA	c.1842T>C	1.0	synonymous_variar	-
BPAL-017	gyrA	c.1959G>C	1.0	synonymous_variar	-
BPAL-017	gyrA	p.Gly668Asp	1.0	missense_variant	-
BPAL-017	Rv0010c	c.384T>C	1.0	synonymous_variar	-
BPAL-017	Rv0010c	p.Ile87Met	1.0	missense_variant	-
BPAL-017	Rv0010c	c.99T>C	1.0	synonymous_variar	-
BPAL-017	fgd1	c.960T>C	1.0	synonymous_variar	-
BPAL-017	mshA	c.21T>C	1.0	synonymous_variar	-
BPAL-017	Rv0565c	c.1317G>A	1.0	synonymous_variar	-
BPAL-017	Rv0565c	c.-108T>C	1.0	upstream_gene_var	-
BPAL-017	nusG	c.-138T>C	1.0	upstream_gene_var	-
BPAL-017	rpoB	p.Pro45Ser	1.0	missense_variant	-
BPAL-017	rpoB	c.3225T>C	1.0	synonymous_variar	-
BPAL-017	rpoC	c.162G>C	1.0	synonymous_variar	-
BPAL-017	rpoC	p.Ala172Val	1.0	missense_variant	-
BPAL-017	rpoC	c.517C>A	1.0	synonymous_variar	-
BPAL-017	rpoC	c.1440C>T	1.0	synonymous_variar	-
BPAL-017	rpoC	p.Gly594Arg	1.0	missense_variant	-
BPAL-017	mmpL5	p.Ile948Val	1.0	missense_variant	-
BPAL-017	mmpL5	p.Thr794Ile	1.0	missense_variant	-
BPAL-017	rpsL	c.-165T>C	1.0	upstream_gene_var	-
BPAL-017	rplC	c.-452C>A	1.0	upstream_gene_var	-

BPAL-017	Rv1129c	c.-28T>C	1.0	upstream_gene_var	-
BPAL-017	fbiC	c.-110G>A	1.0	upstream_gene_var	-
BPAL-017	Rv1258c	c.1029T>C	1.0	synonymous_variar	-
BPAL-017	embR	p.Phe376Leu	0.317073170731707	missense_variant	-
BPAL-017	embR	p.Cys372Gly	0.342105263157894	missense_variant	-
BPAL-017	embR	p.Cys110Tyr	1.0	missense_variant	-
BPAL-017	embR	c.-207C>G	1.0	upstream_gene_var	-
BPAL-017	embR	c.-446C>T	1.0	upstream_gene_var	-
BPAL-017	atpE	c.-138T>C	1.0	upstream_gene_var	-
BPAL-017	rrs	n.-187C>T	1.0	upstream_gene_var	-
BPAL-017	inhA	c.-822C>G	0.2	upstream_gene_var	-
BPAL-017	inhA	c.-40C>T	1.0	upstream_gene_var	-
BPAL-017	tsnR	c.369C>T	1.0	synonymous_variar	-
BPAL-017	tsnR	p.Leu232Pro	1.0	missense_variant	-
BPAL-017	tlyA	c.33A>G	1.0	synonymous_variar	-
BPAL-017	bacA	p.Ile603Val	1.0	missense_variant	-
BPAL-017	katG	p.Arg463Leu	1.0	missense_variant	-
BPAL-017	PPE35	p.Leu896Ser	1.0	missense_variant	-
BPAL-017	Rv1979c	p.Asp286Gly	1.0	missense_variant	-
BPAL-017	Rv1979c	c.-129A>G	1.0	upstream_gene_var	-
BPAL-017	kasA	c.18C>T	1.0	synonymous_variar	-
BPAL-017	kasA	p.Gly312Ser	1.0	missense_variant	-
BPAL-017	ahpC	c.-142G>A	1.0	upstream_gene_var	-
BPAL-017	ald	c.-32T>C	1.0	upstream_gene_var	-
BPAL-017	fbiD	c.300A>G	1.0	synonymous_variar	-
BPAL-017	Rv3083	p.Asp71His	1.0	missense_variant	-
BPAL-017	whiB7	c.188delG	1.0	frameshift_variant	-
BPAL-017	lpqB	p.Asp142Gly	1.0	missense_variant	-
BPAL-017	mtrB	p.Met517Leu	1.0	missense_variant	-
BPAL-017	fbiA	c.15T>C	1.0	synonymous_variar	-
BPAL-017	clpC1	p.Val63Ala	1.0	missense_variant	-
BPAL-017	glpK	p.Val460Ala	1.0	missense_variant	-
BPAL-017	embC	p.Thr270Ile	1.0	missense_variant	-
BPAL-017	embC	p.Asn394Asp	0.954545454545454	missense_variant	-
BPAL-017	embC	c.2781C>T	1.0	synonymous_variar	-
BPAL-017	embA	c.348G>A	1.0	synonymous_variar	-
BPAL-017	embA	c.1188G>C	1.0	synonymous_variar	-
BPAL-017	embA	p.Pro913Ser	1.0	missense_variant	-
BPAL-017	embB	c.1065G>A	1.0	synonymous_variar	-
BPAL-017	embB	p.Glu378Ala	1.0	missense_variant	-
BPAL-017	ubiA	p.Glu149Asp	1.0	missense_variant	-
BPAL-017	ubiA	c.228T>C	1.0	synonymous_variar	-
BPAL-017	ubiA	c.-36delG	1.0	upstream_gene_var	-
BPAL-017	whiB6	p.Arg54Gln	1.0	missense_variant	-
BPAL-017	whiB6	c.-75delG	1.0	upstream_gene_var	-
BPAL-017	whiB6	c.-82C>T	1.0	upstream_gene_var	-
BPAL-017	whiB6	c.-211C>T	1.0	upstream_gene_var	-
BPAL-017	gid	c.615A>G	1.0	synonymous_variar	-
BPAL-017	gid	p.Thr190Lys	1.0	missense_variant	-
BPAL-017	gid	p.Ser131Thr	1.0	missense_variant	-
BPAL-017	gid	c.330G>T	1.0	synonymous_variar	-
BPAL-019	rpoB	p.Ser450Leu	1.0	missense_variant	rifampicin
BPAL-019	rpoC	p.Leu527Val	1.0	missense_variant	rifampicin
BPAL-019	dnaA	c.1302C>A	1.0	synonymous_variar	-
BPAL-019	gyrB	p.Met291Ile	1.0	missense_variant	-
BPAL-019	gyrA	p.Glu21Gln	1.0	missense_variant	-
BPAL-019	gyrA	p.Ser95Thr	1.0	missense_variant	-
BPAL-019	gyrA	p.Ala384Val	1.0	missense_variant	-
BPAL-019	gyrA	c.1842T>C	1.0	synonymous_variar	-

BPAL-019	gyrA	c.1959G>C	1.0	synonymous_variar	-
BPAL-019	gyrA	p.Gly668Asp	1.0	missense_variant	-
BPAL-019	Rv0010c	c.384T>C	1.0	synonymous_variar	-
BPAL-019	Rv0010c	p.Ile87Met	1.0	missense_variant	-
BPAL-019	Rv0010c	c.99T>C	1.0	synonymous_variar	-
BPAL-019	fgd1	c.960T>C	1.0	synonymous_variar	-
BPAL-019	mshA	c.21T>C	1.0	synonymous_variar	-
BPAL-019	Rv0565c	c.-108T>C	1.0	upstream_gene_var	-
BPAL-019	nusG	c.-138T>C	1.0	upstream_gene_var	-
BPAL-019	rpoB	c.3225T>C	1.0	synonymous_variar	-
BPAL-019	rpoC	c.162G>C	1.0	synonymous_variar	-
BPAL-019	rpoC	p.Ala172Val	1.0	missense_variant	-
BPAL-019	rpoC	c.517C>A	1.0	synonymous_variar	-
BPAL-019	mmpL5	p.Ile948Val	1.0	missense_variant	-
BPAL-019	mmpL5	p.Thr794Ile	1.0	missense_variant	-
BPAL-019	rpsL	c.-165T>C	1.0	upstream_gene_var	-
BPAL-019	rplC	c.-452C>A	1.0	upstream_gene_var	-
BPAL-019	Rv1129c	c.-28T>C	1.0	upstream_gene_var	-
BPAL-019	Rv1258c	c.1029T>C	1.0	synonymous_variar	-
BPAL-019	embR	p.Phe376Leu	0.2	missense_variant	-
BPAL-019	embR	p.Cys372Gly	0.244897959183673	missense_variant	-
BPAL-019	embR	p.Cys110Tyr	1.0	missense_variant	-
BPAL-019	embR	c.-207C>G	1.0	upstream_gene_var	-
BPAL-019	embR	c.-446C>T	1.0	upstream_gene_var	-
BPAL-019	atpE	c.-138T>C	1.0	upstream_gene_var	-
BPAL-019	rrs	n.-187C>T	1.0	upstream_gene_var	-
BPAL-019	rrl	n.982G>A	1.0	non_coding_transcr	-
BPAL-019	inhA	c.-40C>T	0.956521739130434	upstream_gene_var	-
BPAL-019	tsnR	c.369C>T	1.0	synonymous_variar	-
BPAL-019	tsnR	p.Leu232Pro	1.0	missense_variant	-
BPAL-019	tlyA	c.33A>G	1.0	synonymous_variar	-
BPAL-019	bacA	p.Ile603Val	1.0	missense_variant	-
BPAL-019	katG	p.Arg463Leu	1.0	missense_variant	-
BPAL-019	PPE35	p.Leu896Ser	1.0	missense_variant	-
BPAL-019	Rv1979c	p.Asp286Gly	1.0	missense_variant	-
BPAL-019	Rv1979c	c.-129A>G	1.0	upstream_gene_var	-
BPAL-019	kasA	c.18C>T	1.0	synonymous_variar	-
BPAL-019	kasA	p.Gly312Ser	1.0	missense_variant	-
BPAL-019	ahpC	c.-142G>A	1.0	upstream_gene_var	-
BPAL-019	ald	c.-32T>C	1.0	upstream_gene_var	-
BPAL-019	fbiD	c.300A>G	1.0	synonymous_variar	-
BPAL-019	Rv3083	p.Asp71His	1.0	missense_variant	-
BPAL-019	whiB7	c.188delG	1.0	frameshift_variant	-
BPAL-019	lpqB	p.Asp142Gly	1.0	missense_variant	-
BPAL-019	mtrB	p.Met517Leu	0.923076923076923	missense_variant	-
BPAL-019	fbiA	c.15T>C	1.0	synonymous_variar	-
BPAL-019	clpC1	p.Val63Ala	1.0	missense_variant	-
BPAL-019	glpK	p.Val460Ala	1.0	missense_variant	-
BPAL-019	embC	p.Thr270Ile	1.0	missense_variant	-
BPAL-019	embC	p.Asn394Asp	1.0	missense_variant	-
BPAL-019	embC	c.2781C>T	1.0	synonymous_variar	-
BPAL-019	embA	c.348G>A	1.0	synonymous_variar	-
BPAL-019	embA	c.1188G>C	1.0	synonymous_variar	-
BPAL-019	embA	p.Pro913Ser	1.0	missense_variant	-
BPAL-019	embB	c.1065G>A	1.0	synonymous_variar	-
BPAL-019	embB	p.Glu378Ala	1.0	missense_variant	-
BPAL-019	ubiA	p.Glu149Asp	1.0	missense_variant	-
BPAL-019	ubiA	c.228T>C	1.0	synonymous_variar	-
BPAL-019	ubiA	c.-36delG	1.0	upstream_gene_var	-

BPAL-019	whiB6	p.Arg54Gln	1.0	missense_variant	-
BPAL-019	whiB6	c.-75delG	1.0	upstream_gene_var	-
BPAL-019	whiB6	c.-82C>T	1.0	upstream_gene_var	-
BPAL-019	whiB6	c.-211C>T	1.0	upstream_gene_var	-
BPAL-019	gid	c.615A>G	1.0	synonymous_variar	-
BPAL-019	gid	c.330G>T	1.0	synonymous_variar	-
BPAL-020	rpoB	p.Leu452Pro	1.0	missense_variant	rifampicin
BPAL-020	rpsL	p.Lys43Arg	1.0	missense_variant	streptomycin
BPAL-020	katG	p.Ser315Thr	1.0	missense_variant	isoniazid
BPAL-020	embB	p.Met306Ile	1.0	missense_variant	ethambutol
BPAL-020	dnaA	c.1302C>A	1.0	synonymous_variar	-
BPAL-020	gyrB	p.Met291Ile	1.0	missense_variant	-
BPAL-020	gyrA	p.Glu21Gln	1.0	missense_variant	-
BPAL-020	gyrA	p.Ser95Thr	1.0	missense_variant	-
BPAL-020	gyrA	p.Ala384Val	1.0	missense_variant	-
BPAL-020	gyrA	c.1842T>C	1.0	synonymous_variar	-
BPAL-020	gyrA	c.1959G>C	1.0	synonymous_variar	-
BPAL-020	gyrA	p.Gly668Asp	1.0	missense_variant	-
BPAL-020	Rv0010c	c.384T>C	1.0	synonymous_variar	-
BPAL-020	Rv0010c	p.Ile87Met	1.0	missense_variant	-
BPAL-020	Rv0010c	c.99T>C	1.0	synonymous_variar	-
BPAL-020	fgd1	c.960T>C	1.0	synonymous_variar	-
BPAL-020	mshA	c.21T>C	1.0	synonymous_variar	-
BPAL-020	Rv0565c	c.-108T>C	1.0	upstream_gene_var	-
BPAL-020	nusG	c.-138T>C	1.0	upstream_gene_var	-
BPAL-020	rpoB	c.3225T>C	1.0	synonymous_variar	-
BPAL-020	rpoC	c.162G>C	1.0	synonymous_variar	-
BPAL-020	rpoC	p.Ala172Val	1.0	missense_variant	-
BPAL-020	rpoC	c.517C>A	1.0	synonymous_variar	-
BPAL-020	mmpL5	p.Ile948Val	1.0	missense_variant	-
BPAL-020	mmpL5	p.Thr794Ile	1.0	missense_variant	-
BPAL-020	rpsL	c.-165T>C	1.0	upstream_gene_var	-
BPAL-020	rplC	c.-452C>A	1.0	upstream_gene_var	-
BPAL-020	Rv1129c	c.-28T>C	1.0	upstream_gene_var	-
BPAL-020	Rv1258c	c.1029T>C	1.0	synonymous_variar	-
BPAL-020	embR	p.Phe376Leu	0.268292682926829	missense_variant	-
BPAL-020	embR	p.Cys372Gly	0.282051282051282	missense_variant	-
BPAL-020	embR	p.Cys110Tyr	1.0	missense_variant	-
BPAL-020	embR	c.-207C>G	1.0	upstream_gene_var	-
BPAL-020	embR	c.-446C>T	1.0	upstream_gene_var	-
BPAL-020	atpE	c.-138T>C	0.966666666666666	upstream_gene_var	-
BPAL-020	rrs	n.-187C>T	1.0	upstream_gene_var	-
BPAL-020	rrl	n.982G>A	1.0	non_coding_transcı	-
BPAL-020	inhA	c.-40C>T	0.965517241379310	upstream_gene_var	-
BPAL-020	tsnR	c.369C>T	1.0	synonymous_variar	-
BPAL-020	tsnR	p.Leu232Pro	1.0	missense_variant	-
BPAL-020	tlyA	c.33A>G	1.0	synonymous_variar	-
BPAL-020	bacA	p.Ile603Val	1.0	missense_variant	-
BPAL-020	katG	p.Arg463Leu	1.0	missense_variant	-
BPAL-020	PPE35	p.Leu896Ser	1.0	missense_variant	-
BPAL-020	Rv1979c	p.Asp286Gly	1.0	missense_variant	-
BPAL-020	Rv1979c	c.-129A>G	1.0	upstream_gene_var	-
BPAL-020	kasA	c.18C>T	1.0	synonymous_variar	-
BPAL-020	kasA	p.Gly312Ser	1.0	missense_variant	-
BPAL-020	ahpC	c.-142G>A	1.0	upstream_gene_var	-
BPAL-020	ald	c.-32T>C	1.0	upstream_gene_var	-
BPAL-020	fbiD	c.300A>G	1.0	synonymous_variar	-
BPAL-020	Rv3083	p.Asp71His	1.0	missense_variant	-
BPAL-020	whiB7	c.188delG	1.0	frameshift_variant	-

BPAL-020	lpqB	p.Asp142Gly	1.0	missense_variant	-
BPAL-020	fbiA	c.15T>C	1.0	synonymous_variar	-
BPAL-020	clpC1	p.Val63Ala	1.0	missense_variant	-
BPAL-020	clpC1	c.-109A>G	1.0	upstream_gene_var	-
BPAL-020	glpK	p.Val460Ala	1.0	missense_variant	-
BPAL-020	embC	p.Thr270Ile	1.0	missense_variant	-
BPAL-020	embC	p.Asn394Asp	0.9	missense_variant	-
BPAL-020	embC	c.2781C>T	1.0	synonymous_variar	-
BPAL-020	embA	c.348G>A	1.0	synonymous_variar	-
BPAL-020	embA	c.1188G>C	1.0	synonymous_variar	-
BPAL-020	embA	p.Pro913Ser	1.0	missense_variant	-
BPAL-020	embB	c.1065G>A	1.0	synonymous_variar	-
BPAL-020	embB	p.Glu378Ala	1.0	missense_variant	-
BPAL-020	ubiA	p.Glu149Asp	1.0	missense_variant	-
BPAL-020	ubiA	c.228T>C	1.0	synonymous_variar	-
BPAL-020	ubiA	c.-36delG	1.0	upstream_gene_var	-
BPAL-020	whiB6	p.Arg54Gln	1.0	missense_variant	-
BPAL-020	whiB6	c.-75delG	1.0	upstream_gene_var	-
BPAL-020	whiB6	c.-82C>T	1.0	upstream_gene_var	-
BPAL-020	whiB6	c.-211C>T	1.0	upstream_gene_var	-
BPAL-020	gid	c.615A>G	1.0	synonymous_variar	-
BPAL-020	gid	c.330G>T	1.0	synonymous_variar	-
BPAL-021	rpoB	p.Ser450Leu	1.0	missense_variant	rifampicin
BPAL-021	dnaA	c.1302C>A	1.0	synonymous_variar	-
BPAL-021	gyrB	p.Met291Ile	1.0	missense_variant	-
BPAL-021	gyrA	p.Glu21Gln	1.0	missense_variant	-
BPAL-021	gyrA	c.267C>T	1.0	synonymous_variar	-
BPAL-021	gyrA	p.Ser95Thr	1.0	missense_variant	-
BPAL-021	gyrA	p.Ala384Val	1.0	missense_variant	-
BPAL-021	gyrA	c.1842T>C	1.0	synonymous_variar	-
BPAL-021	gyrA	c.1959G>C	1.0	synonymous_variar	-
BPAL-021	gyrA	p.Gly668Asp	1.0	missense_variant	-
BPAL-021	Rv0010c	c.384T>C	1.0	synonymous_variar	-
BPAL-021	Rv0010c	p.Ile87Met	1.0	missense_variant	-
BPAL-021	Rv0010c	c.99T>C	1.0	synonymous_variar	-
BPAL-021	fgd1	c.960T>C	1.0	synonymous_variar	-
BPAL-021	mshA	c.21T>C	1.0	synonymous_variar	-
BPAL-021	Rv0565c	c.-108T>C	1.0	upstream_gene_var	-
BPAL-021	nusG	c.-138T>C	1.0	upstream_gene_var	-
BPAL-021	rpoB	p.Thr399Ala	1.0	missense_variant	-
BPAL-021	rpoB	c.3225T>C	1.0	synonymous_variar	-
BPAL-021	rpoC	c.162G>C	1.0	synonymous_variar	-
BPAL-021	rpoC	p.Ala172Val	1.0	missense_variant	-
BPAL-021	rpoC	c.517C>A	1.0	synonymous_variar	-
BPAL-021	mmpL5	p.Ile948Val	1.0	missense_variant	-
BPAL-021	mmpL5	p.Thr794Ile	1.0	missense_variant	-
BPAL-021	rpsL	c.-165T>C	1.0	upstream_gene_var	-
BPAL-021	rplC	c.-452C>A	1.0	upstream_gene_var	-
BPAL-021	Rv1129c	c.-28T>C	1.0	upstream_gene_var	-
BPAL-021	Rv1258c	c.1029T>C	1.0	synonymous_variar	-
BPAL-021	embR	p.Phe376Leu	0.238805970149253	missense_variant	-
BPAL-021	embR	p.Cys372Gly	0.235294117647058	missense_variant	-
BPAL-021	embR	p.Cys110Tyr	1.0	missense_variant	-
BPAL-021	embR	c.-207C>G	1.0	upstream_gene_var	-
BPAL-021	embR	c.-446C>T	1.0	upstream_gene_var	-
BPAL-021	atpE	c.-138T>C	1.0	upstream_gene_var	-
BPAL-021	rrs	n.-187C>T	1.0	upstream_gene_var	-
BPAL-021	rrl	n.982G>A	1.0	non_coding_transcı	-
BPAL-021	inhA	c.-40C>T	1.0	upstream_gene_var	-

BPAL-021	tsnR	c.369C>T	1.0	synonymous_variar	-
BPAL-021	tsnR	p.Leu232Pro	1.0	missense_variant	-
BPAL-021	tlyA	c.33A>G	1.0	synonymous_variar	-
BPAL-021	bacA	p.Ile603Val	1.0	missense_variant	-
BPAL-021	katG	p.Arg463Leu	1.0	missense_variant	-
BPAL-021	PPE35	p.Leu896Ser	1.0	missense_variant	-
BPAL-021	PPE35	p.Thr414Ala	1.0	missense_variant	-
BPAL-021	Rv1979c	p.Asp286Gly	1.0	missense_variant	-
BPAL-021	Rv1979c	c.-129A>G	1.0	upstream_gene_var	-
BPAL-021	kasA	c.18C>T	1.0	synonymous_variar	-
BPAL-021	kasA	p.Gly312Ser	1.0	missense_variant	-
BPAL-021	ahpC	c.-142G>A	1.0	upstream_gene_var	-
BPAL-021	ald	c.-32T>C	1.0	upstream_gene_var	-
BPAL-021	fbiD	c.300A>G	1.0	synonymous_variar	-
BPAL-021	Rv3083	p.Asp71His	1.0	missense_variant	-
BPAL-021	whiB7	c.188delG	1.0	frameshift_variant	-
BPAL-021	lpqB	p.Asp142Gly	1.0	missense_variant	-
BPAL-021	mtrB	p.Met517Leu	1.0	missense_variant	-
BPAL-021	fbiA	c.15T>C	1.0	synonymous_variar	-
BPAL-021	clpC1	p.Val63Ala	1.0	missense_variant	-
BPAL-021	glpK	p.Val460Ala	1.0	missense_variant	-
BPAL-021	embC	p.Thr270Ile	1.0	missense_variant	-
BPAL-021	embC	p.Asn394Asp	1.0	missense_variant	-
BPAL-021	embC	c.2781C>T	1.0	synonymous_variar	-
BPAL-021	embA	c.348G>A	1.0	synonymous_variar	-
BPAL-021	embA	c.1188G>C	1.0	synonymous_variar	-
BPAL-021	embA	p.Pro913Ser	1.0	missense_variant	-
BPAL-021	embB	c.1065G>A	1.0	synonymous_variar	-
BPAL-021	embB	p.Glu378Ala	1.0	missense_variant	-
BPAL-021	ubiA	p.Glu149Asp	1.0	missense_variant	-
BPAL-021	ubiA	c.228T>C	1.0	synonymous_variar	-
BPAL-021	ubiA	c.-36delG	1.0	upstream_gene_var	-
BPAL-021	whiB6	p.Arg54Gln	1.0	missense_variant	-
BPAL-021	whiB6	c.-75delG	1.0	upstream_gene_var	-
BPAL-021	whiB6	c.-82C>T	1.0	upstream_gene_var	-
BPAL-021	whiB6	c.-211C>T	1.0	upstream_gene_var	-
BPAL-021	gid	c.615A>G	1.0	synonymous_variar	-
BPAL-021	gid	c.330G>T	0.982758620689655	synonymous_variar	-
BPAL-022	rpoB	p.His445Tyr	1.0	missense_variant	rifampicin
BPAL-022	inhA	c.-777C>T	1.0	upstream_gene_var	ethionamide;isoniazid
BPAL-022	katG	p.Ser315Thr	1.0	missense_variant	isoniazid
BPAL-022	pncA	p.Asp12Glu	1.0	missense_variant	pyrazinamide
BPAL-022	dnaA	c.1302C>A	1.0	synonymous_variar	-
BPAL-022	gyrB	p.Met291Ile	1.0	missense_variant	-
BPAL-022	gyrA	p.Glu21Gln	1.0	missense_variant	-
BPAL-022	gyrA	p.Ser95Thr	1.0	missense_variant	-
BPAL-022	gyrA	p.Ala384Val	1.0	missense_variant	-
BPAL-022	gyrA	c.1842T>C	1.0	synonymous_variar	-
BPAL-022	gyrA	c.1959G>C	1.0	synonymous_variar	-
BPAL-022	gyrA	p.Gly668Asp	1.0	missense_variant	-
BPAL-022	Rv0010c	c.384T>C	1.0	synonymous_variar	-
BPAL-022	Rv0010c	p.Ile87Met	1.0	missense_variant	-
BPAL-022	Rv0010c	c.99T>C	1.0	synonymous_variar	-
BPAL-022	fgd1	c.960T>C	1.0	synonymous_variar	-
BPAL-022	mshA	c.21T>C	1.0	synonymous_variar	-
BPAL-022	Rv0565c	c.-108T>C	1.0	upstream_gene_var	-
BPAL-022	nusG	c.-138T>C	1.0	upstream_gene_var	-
BPAL-022	rpoB	c.3225T>C	1.0	synonymous_variar	-
BPAL-022	rpoC	c.162G>C	1.0	synonymous_variar	-

BPAL-022	rpoC	p.Ala172Val	1.0	missense_variant	-
BPAL-022	rpoC	c.517C>A	1.0	synonymous_variar	-
BPAL-022	mmpL5	p.Ile948Val	1.0	missense_variant	-
BPAL-022	mmpL5	p.Thr794Ile	1.0	missense_variant	-
BPAL-022	rpsL	c.-165T>C	1.0	upstream_gene_var	-
BPAL-022	rplC	c.-452C>A	1.0	upstream_gene_var	-
BPAL-022	Rv1129c	c.-28T>C	1.0	upstream_gene_var	-
BPAL-022	Rv1258c	c.1029T>C	1.0	synonymous_variar	-
BPAL-022	embR	p.Cys110Tyr	0.977777777777777	missense_variant	-
BPAL-022	embR	c.-207C>G	1.0	upstream_gene_var	-
BPAL-022	embR	c.-446C>T	1.0	upstream_gene_var	-
BPAL-022	atpE	c.-138T>C	1.0	upstream_gene_var	-
BPAL-022	rrs	n.-187C>T	1.0	upstream_gene_var	-
BPAL-022	rrl	n.982G>A	1.0	non_coding_transcı	-
BPAL-022	inhA	c.-40C>T	1.0	upstream_gene_var	-
BPAL-022	tsnR	c.369C>T	1.0	synonymous_variar	-
BPAL-022	tsnR	p.Leu232Pro	1.0	missense_variant	-
BPAL-022	tlyA	c.33A>G	1.0	synonymous_variar	-
BPAL-022	bacA	p.Ile603Val	1.0	missense_variant	-
BPAL-022	katG	p.Arg463Leu	0.972222222222222	missense_variant	-
BPAL-022	PPE35	p.Leu896Ser	1.0	missense_variant	-
BPAL-022	Rv1979c	p.Asp286Gly	1.0	missense_variant	-
BPAL-022	Rv1979c	c.-129A>G	1.0	upstream_gene_var	-
BPAL-022	kasA	c.18C>T	1.0	synonymous_variar	-
BPAL-022	kasA	p.Gly312Ser	1.0	missense_variant	-
BPAL-022	ahpC	c.-142G>A	1.0	upstream_gene_var	-
BPAL-022	ald	c.-32T>C	1.0	upstream_gene_var	-
BPAL-022	fbiD	c.300A>G	1.0	synonymous_variar	-
BPAL-022	Rv3083	p.Asp71His	1.0	missense_variant	-
BPAL-022	whiB7	c.188delG	1.0	frameshift_variant	-
BPAL-022	lpqB	p.Asp142Gly	1.0	missense_variant	-
BPAL-022	mtrB	p.Met517Leu	1.0	missense_variant	-
BPAL-022	fbiA	c.15T>C	1.0	synonymous_variar	-
BPAL-022	clpC1	p.Val63Ala	1.0	missense_variant	-
BPAL-022	glpK	p.Val460Ala	1.0	missense_variant	-
BPAL-022	embC	p.Thr270Ile	1.0	missense_variant	-
BPAL-022	embC	p.Asn394Asp	1.0	missense_variant	-
BPAL-022	embC	c.2781C>T	1.0	synonymous_variar	-
BPAL-022	embA	c.348G>A	1.0	synonymous_variar	-
BPAL-022	embA	c.1188G>C	1.0	synonymous_variar	-
BPAL-022	embA	p.Pro913Ser	1.0	missense_variant	-
BPAL-022	embB	c.1065G>A	1.0	synonymous_variar	-
BPAL-022	embB	p.Glu378Ala	1.0	missense_variant	-
BPAL-022	ubiA	p.Glu149Asp	1.0	missense_variant	-
BPAL-022	ubiA	c.228T>C	1.0	synonymous_variar	-
BPAL-022	ubiA	c.-36delG	1.0	upstream_gene_var	-
BPAL-022	whiB6	p.Arg54Gln	1.0	missense_variant	-
BPAL-022	whiB6	c.-75delG	1.0	upstream_gene_var	-
BPAL-022	whiB6	c.-82C>T	1.0	upstream_gene_var	-
BPAL-022	whiB6	c.-211C>T	1.0	upstream_gene_var	-
BPAL-022	gid	c.615A>G	1.0	synonymous_variar	-
BPAL-022	gid	c.330G>T	0.986111111111111	synonymous_variar	-
BPAL-023	rpoB	p.His445Asn	1.0	missense_variant	rifampicin
BPAL-023	inhA	c.-777C>T	1.0	upstream_gene_var	ethionamide;isoniazid
BPAL-023	inhA	p.Ile194Thr	1.0	missense_variant	ethionamide;isoniazid
BPAL-023	gid	c.98delG	1.0	frameshift_variant	streptomycin
BPAL-023	dnaA	c.1302C>A	1.0	synonymous_variar	-
BPAL-023	gyrB	p.Met291Ile	1.0	missense_variant	-
BPAL-023	gyrA	p.Glu21Gln	1.0	missense_variant	-

BPAL-023	gyrA	p.Ser95Thr	1.0	missense_variant	-
BPAL-023	gyrA	p.Ala384Val	1.0	missense_variant	-
BPAL-023	gyrA	c.1842T>C	1.0	synonymous_variar	-
BPAL-023	gyrA	c.1959G>C	1.0	synonymous_variar	-
BPAL-023	gyrA	p.Gly668Asp	1.0	missense_variant	-
BPAL-023	Rv0010c	c.384T>C	1.0	synonymous_variar	-
BPAL-023	Rv0010c	p.Ile87Met	1.0	missense_variant	-
BPAL-023	Rv0010c	c.99T>C	1.0	synonymous_variar	-
BPAL-023	fgd1	c.960T>C	1.0	synonymous_variar	-
BPAL-023	mshA	c.21T>C	1.0	synonymous_variar	-
BPAL-023	Rv0565c	c.-108T>C	1.0	upstream_gene_var	-
BPAL-023	nusG	c.-138T>C	1.0	upstream_gene_var	-
BPAL-023	rpoB	c.3225T>C	1.0	synonymous_variar	-
BPAL-023	rpoC	c.162G>C	1.0	synonymous_variar	-
BPAL-023	rpoC	p.Ala172Val	0.964285714285714	missense_variant	-
BPAL-023	rpoC	c.517C>A	1.0	synonymous_variar	-
BPAL-023	mmpL5	p.Ile948Val	1.0	missense_variant	-
BPAL-023	mmpL5	p.Thr794Ile	1.0	missense_variant	-
BPAL-023	rpsL	c.-165T>C	1.0	upstream_gene_var	-
BPAL-023	rplC	c.-452C>A	1.0	upstream_gene_var	-
BPAL-023	Rv1129c	c.-28T>C	1.0	upstream_gene_var	-
BPAL-023	Rv1258c	c.1029T>C	1.0	synonymous_variar	-
BPAL-023	embR	p.Phe376Leu	0.2	missense_variant	-
BPAL-023	embR	p.Cys372Gly	0.230769230769230	missense_variant	-
BPAL-023	embR	p.Cys110Tyr	1.0	missense_variant	-
BPAL-023	embR	c.-207C>G	1.0	upstream_gene_var	-
BPAL-023	embR	c.-446C>T	1.0	upstream_gene_var	-
BPAL-023	atpE	c.-138T>C	1.0	upstream_gene_var	-
BPAL-023	rrs	n.-187C>T	0.964285714285714	upstream_gene_var	-
BPAL-023	rrl	n.982G>A	1.0	non_coding_transcı	-
BPAL-023	inhA	c.-40C>T	1.0	upstream_gene_var	-
BPAL-023	inhA	c.714C>T	1.0	synonymous_variar	-
BPAL-023	tsnR	c.369C>T	1.0	synonymous_variar	-
BPAL-023	tsnR	p.Leu232Pro	1.0	missense_variant	-
BPAL-023	tlyA	c.33A>G	1.0	synonymous_variar	-
BPAL-023	bacA	p.Ile603Val	1.0	missense_variant	-
BPAL-023	katG	p.Arg463Leu	1.0	missense_variant	-
BPAL-023	PPE35	p.Leu896Ser	1.0	missense_variant	-
BPAL-023	Rv1979c	p.Asp286Gly	1.0	missense_variant	-
BPAL-023	Rv1979c	c.-129A>G	1.0	upstream_gene_var	-
BPAL-023	kasA	c.18C>T	1.0	synonymous_variar	-
BPAL-023	kasA	p.Gly312Ser	1.0	missense_variant	-
BPAL-023	ahpC	c.-142G>A	1.0	upstream_gene_var	-
BPAL-023	ald	c.-32T>C	1.0	upstream_gene_var	-
BPAL-023	fbiD	c.300A>G	1.0	synonymous_variar	-
BPAL-023	Rv3083	p.Asp71His	0.961538461538461	missense_variant	-
BPAL-023	whiB7	c.188delG	1.0	frameshift_variant	-
BPAL-023	lpqB	p.Asp142Gly	1.0	missense_variant	-
BPAL-023	mtrB	p.Met517Leu	1.0	missense_variant	-
BPAL-023	fbiA	c.15T>C	1.0	synonymous_variar	-
BPAL-023	clpC1	p.Val63Ala	1.0	missense_variant	-
BPAL-023	glpK	p.Val460Ala	1.0	missense_variant	-
BPAL-023	embC	p.Thr270Ile	1.0	missense_variant	-
BPAL-023	embC	p.Asn394Asp	1.0	missense_variant	-
BPAL-023	embC	c.2781C>T	1.0	synonymous_variar	-
BPAL-023	embA	c.348G>A	1.0	synonymous_variar	-
BPAL-023	embA	c.1188G>C	1.0	synonymous_variar	-
BPAL-023	embA	p.Pro913Ser	1.0	missense_variant	-
BPAL-023	embB	c.1065G>A	1.0	synonymous_variar	-

BPAL-023	embB	p.Glu378Ala	1.0	missense_variant	-
BPAL-023	embB	p.Tyr733Asp	1.0	missense_variant	-
BPAL-023	ubiA	p.Glu149Asp	1.0	missense_variant	-
BPAL-023	ubiA	c.228T>C	1.0	synonymous_variar	-
BPAL-023	ubiA	c.-36delG	1.0	upstream_gene_var	-
BPAL-023	whiB6	p.Arg54Gln	1.0	missense_variant	-
BPAL-023	whiB6	c.-75delG	1.0	upstream_gene_var	-
BPAL-023	whiB6	c.-82C>T	1.0	upstream_gene_var	-
BPAL-023	whiB6	c.-211C>T	1.0	upstream_gene_var	-
BPAL-023	gid	c.615A>G	1.0	synonymous_variar	-
BPAL-023	gid	c.330G>T	1.0	synonymous_variar	-
BPAL-026	rpoB	p.Ser450Leu	1.0	missense_variant	rifampicin
BPAL-026	katG	p.Ser315Thr	1.0	missense_variant	isoniazid
BPAL-026	dnaA	c.1302C>A	1.0	synonymous_variar	-
BPAL-026	gyrB	p.Met291Ile	1.0	missense_variant	-
BPAL-026	gyrA	p.Glu21Gln	1.0	missense_variant	-
BPAL-026	gyrA	p.Ser95Thr	1.0	missense_variant	-
BPAL-026	gyrA	p.Ala384Val	1.0	missense_variant	-
BPAL-026	gyrA	c.1842T>C	1.0	synonymous_variar	-
BPAL-026	gyrA	c.1959G>C	1.0	synonymous_variar	-
BPAL-026	gyrA	p.Gly668Asp	1.0	missense_variant	-
BPAL-026	Rv0010c	c.384T>C	1.0	synonymous_variar	-
BPAL-026	Rv0010c	p.Ile87Met	1.0	missense_variant	-
BPAL-026	Rv0010c	c.99T>C	1.0	synonymous_variar	-
BPAL-026	fgd1	c.960T>C	1.0	synonymous_variar	-
BPAL-026	mshA	c.21T>C	1.0	synonymous_variar	-
BPAL-026	Rv0565c	c.-108T>C	1.0	upstream_gene_var	-
BPAL-026	nusG	c.-138T>C	1.0	upstream_gene_var	-
BPAL-026	rpoB	c.3225T>C	1.0	synonymous_variar	-
BPAL-026	rpoC	c.162G>C	1.0	synonymous_variar	-
BPAL-026	rpoC	p.Ala172Val	1.0	missense_variant	-
BPAL-026	rpoC	c.517C>A	1.0	synonymous_variar	-
BPAL-026	mmpL5	p.Ile948Val	1.0	missense_variant	-
BPAL-026	mmpL5	p.Thr794Ile	1.0	missense_variant	-
BPAL-026	rpsL	c.-165T>C	1.0	upstream_gene_var	-
BPAL-026	rplC	c.-452C>A	1.0	upstream_gene_var	-
BPAL-026	Rv1129c	c.-28T>C	1.0	upstream_gene_var	-
BPAL-026	Rv1258c	c.1029T>C	1.0	synonymous_variar	-
BPAL-026	embR	p.Cys110Tyr	1.0	missense_variant	-
BPAL-026	embR	c.-207C>G	1.0	upstream_gene_var	-
BPAL-026	embR	c.-446C>T	1.0	upstream_gene_var	-
BPAL-026	atpE	c.-138T>C	1.0	upstream_gene_var	-
BPAL-026	rrs	n.-187C>T	1.0	upstream_gene_var	-
BPAL-026	rrl	n.982G>A	1.0	non_coding_transcı	-
BPAL-026	inhA	c.-40C>T	1.0	upstream_gene_var	-
BPAL-026	tsnR	c.369C>T	1.0	synonymous_variar	-
BPAL-026	tsnR	p.Leu232Pro	1.0	missense_variant	-
BPAL-026	tlyA	c.33A>G	1.0	synonymous_variar	-
BPAL-026	bacA	p.Ile603Val	1.0	missense_variant	-
BPAL-026	katG	p.Arg463Leu	1.0	missense_variant	-
BPAL-026	PPE35	p.Leu896Ser	1.0	missense_variant	-
BPAL-026	Rv1979c	p.Asp286Gly	1.0	missense_variant	-
BPAL-026	Rv1979c	c.-129A>G	1.0	upstream_gene_var	-
BPAL-026	kasA	c.18C>T	1.0	synonymous_variar	-
BPAL-026	kasA	p.Gly312Ser	1.0	missense_variant	-
BPAL-026	ahpC	c.-142G>A	1.0	upstream_gene_var	-
BPAL-026	Rv2752c	c.-483G>A	1.0	upstream_gene_var	-
BPAL-026	ald	c.-32T>C	0.962962962962962	upstream_gene_var	-
BPAL-026	fbiD	c.300A>G	0.947368421052631	synonymous_variar	-
BPAL-026	Rv3083	p.Asp71His	1.0	missense_variant	-
BPAL-026	whiB7	c.188delG	1.0	frameshift_variant	-
BPAL-026	lpqB	p.Asp142Gly	1.0	missense_variant	-

BPAL-026	mtrB	p.Met517Leu	1.0	missense_variant	-
BPAL-026	fbiA	c.15T>C	1.0	synonymous_variar	-
BPAL-026	clpC1	p.Val63Ala	1.0	missense_variant	-
BPAL-026	glpK	p.Val460Ala	1.0	missense_variant	-
BPAL-026	glpK	c.565_566ins	1.0	frameshift_variant	-
BPAL-026	embC	p.Thr270Ile	1.0	missense_variant	-
BPAL-026	embC	p.Asn394Asp	1.0	missense_variant	-
BPAL-026	embC	c.2781C>T	1.0	synonymous_variar	-
BPAL-026	embA	c.348G>A	1.0	synonymous_variar	-
BPAL-026	embA	c.1188G>C	1.0	synonymous_variar	-
BPAL-026	embA	c.1995C>T	1.0	synonymous_variar	-
BPAL-026	embA	p.Pro913Ser	1.0	missense_variant	-
BPAL-026	embB	c.1065G>A	1.0	synonymous_variar	-
BPAL-026	embB	p.Glu378Ala	1.0	missense_variant	-
BPAL-026	ubiA	p.Glu149Asp	1.0	missense_variant	-
BPAL-026	ubiA	c.228T>C	1.0	synonymous_variar	-
BPAL-026	ubiA	c.-36delG	1.0	upstream_gene_var	-
BPAL-026	whiB6	p.Arg54Gln	1.0	missense_variant	-
BPAL-026	whiB6	c.-75delG	1.0	upstream_gene_var	-
BPAL-026	whiB6	c.-82C>T	1.0	upstream_gene_var	-
BPAL-026	whiB6	c.-211C>T	1.0	upstream_gene_var	-
BPAL-026	gid	c.615A>G	1.0	synonymous_variar	-
BPAL-026	gid	c.330G>T	1.0	synonymous_variar	-
BPAL-028	rpoB	p.His445Asn	1.0	missense_variant	rifampicin
BPAL-028	inhA	c.-777C>T	1.0	upstream_gene_var	ethionamide;isoniazid
BPAL-028	inhA	p.Ile194Thr	1.0	missense_variant	ethionamide;isoniazid
BPAL-028	gid	c.98delG	1.0	frameshift_variant	streptomycin
BPAL-028	dnaA	c.1302C>A	1.0	synonymous_variar	-
BPAL-028	gyrB	p.Met291Ile	1.0	missense_variant	-
BPAL-028	gyrA	p.Glu21Gln	1.0	missense_variant	-
BPAL-028	gyrA	p.Ser95Thr	1.0	missense_variant	-
BPAL-028	gyrA	p.Ala384Val	1.0	missense_variant	-
BPAL-028	gyrA	c.1842T>C	1.0	synonymous_variar	-
BPAL-028	gyrA	c.1959G>C	1.0	synonymous_variar	-
BPAL-028	gyrA	p.Gly668Asp	1.0	missense_variant	-
BPAL-028	Rv0010c	c.384T>C	1.0	synonymous_variar	-
BPAL-028	Rv0010c	p.Ile87Met	1.0	missense_variant	-
BPAL-028	Rv0010c	c.99T>C	0.956521739130434	synonymous_variar	-
BPAL-028	fgd1	c.960T>C	1.0	synonymous_variar	-
BPAL-028	mshA	c.21T>C	1.0	synonymous_variar	-
BPAL-028	Rv0565c	c.-108T>C	1.0	upstream_gene_var	-
BPAL-028	nusG	c.-138T>C	1.0	upstream_gene_var	-
BPAL-028	rpoB	c.3225T>C	0.964285714285714	synonymous_variar	-
BPAL-028	rpoC	c.162G>C	1.0	synonymous_variar	-
BPAL-028	rpoC	p.Ala172Val	1.0	missense_variant	-
BPAL-028	rpoC	c.517C>A	1.0	synonymous_variar	-
BPAL-028	mmpL5	p.Ile948Val	1.0	missense_variant	-
BPAL-028	mmpL5	p.Thr794Ile	1.0	missense_variant	-
BPAL-028	rpsL	c.-165T>C	1.0	upstream_gene_var	-
BPAL-028	rplC	c.-452C>A	1.0	upstream_gene_var	-
BPAL-028	Rv1129c	c.-28T>C	1.0	upstream_gene_var	-
BPAL-028	Rv1258c	c.1029T>C	1.0	synonymous_variar	-
BPAL-028	embR	p.Phe376Leu	0.363636363636363	missense_variant	-
BPAL-028	embR	p.Cys372Gly	0.382352941176470	missense_variant	-
BPAL-028	embR	p.Cys110Tyr	1.0	missense_variant	-
BPAL-028	embR	c.-207C>G	1.0	upstream_gene_var	-
BPAL-028	embR	c.-446C>T	1.0	upstream_gene_var	-
BPAL-028	atpE	c.-138T>C	1.0	upstream_gene_var	-
BPAL-028	rrs	n.-187C>T	1.0	upstream_gene_var	-
BPAL-028	rrl	n.982G>A	1.0	non_coding_transcr	-
BPAL-028	rrl	n.1864T>G	0.222222222222222	non_coding_transcr	-
BPAL-028	inhA	c.-40C>T	0.976744186046511	upstream_gene_var	-

BPAL-028	inhA	c.714C>T	1.0	synonymous_variar	-
BPAL-028	tsnR	c.369C>T	1.0	synonymous_variar	-
BPAL-028	tsnR	p.Leu232Pro	1.0	missense_variant	-
BPAL-028	tlyA	c.33A>G	1.0	synonymous_variar	-
BPAL-028	bacA	p.Ile603Val	1.0	missense_variant	-
BPAL-028	katG	p.Arg463Leu	1.0	missense_variant	-
BPAL-028	PPE35	p.Leu896Ser	1.0	missense_variant	-
BPAL-028	Rv1979c	p.Asp286Gly	1.0	missense_variant	-
BPAL-028	Rv1979c	c.-129A>G	1.0	upstream_gene_var	-
BPAL-028	kasA	c.18C>T	1.0	synonymous_variar	-
BPAL-028	kasA	p.Gly312Ser	1.0	missense_variant	-
BPAL-028	ahpC	c.-142G>A	1.0	upstream_gene_var	-
BPAL-028	ald	c.-32T>C	1.0	upstream_gene_var	-
BPAL-028	fbiD	c.300A>G	1.0	synonymous_variar	-
BPAL-028	Rv3083	p.Asp71His	1.0	missense_variant	-
BPAL-028	whiB7	c.188delG	1.0	frameshift_variant	-
BPAL-028	lpqB	p.Asp142Gly	1.0	missense_variant	-
BPAL-028	mtrB	p.Met517Leu	1.0	missense_variant	-
BPAL-028	fbiA	c.15T>C	1.0	synonymous_variar	-
BPAL-028	clpC1	p.Val63Ala	1.0	missense_variant	-
BPAL-028	glpK	p.Val460Ala	1.0	missense_variant	-
BPAL-028	embC	p.Thr270Ile	1.0	missense_variant	-
BPAL-028	embC	p.Asn394Asp	1.0	missense_variant	-
BPAL-028	embC	c.2781C>T	1.0	synonymous_variar	-
BPAL-028	embA	c.348G>A	1.0	synonymous_variar	-
BPAL-028	embA	c.1188G>C	1.0	synonymous_variar	-
BPAL-028	embA	p.Pro913Ser	1.0	missense_variant	-
BPAL-028	embB	c.1065G>A	1.0	synonymous_variar	-
BPAL-028	embB	p.Glu378Ala	1.0	missense_variant	-
BPAL-028	embB	p.Tyr733Asp	1.0	missense_variant	-
BPAL-028	ubiA	p.Glu149Asp	1.0	missense_variant	-
BPAL-028	ubiA	c.228T>C	1.0	synonymous_variar	-
BPAL-028	ubiA	c.-36delG	1.0	upstream_gene_var	-
BPAL-028	whiB6	p.Arg54Gln	1.0	missense_variant	-
BPAL-028	whiB6	c.-75delG	1.0	upstream_gene_var	-
BPAL-028	whiB6	c.-82C>T	1.0	upstream_gene_var	-
BPAL-028	whiB6	c.-211C>T	1.0	upstream_gene_var	-
BPAL-028	gid	c.615A>G	1.0	synonymous_variar	-
BPAL-028	gid	c.330G>T	1.0	synonymous_variar	-
BPAL-029	rpoB	p.Ser450Leu	1.0	missense_variant	rifampicin
BPAL-029	katG	p.Ser315Thr	1.0	missense_variant	isoniazid
BPAL-029	pncA	c.-11A>C	1.0	upstream_gene_var	pyrazinamide
BPAL-029	embB	p.Met306Ile	1.0	missense_variant	ethambutol
BPAL-029	dnaA	c.1302C>A	1.0	synonymous_variar	-
BPAL-029	gyrB	p.Met291Ile	1.0	missense_variant	-
BPAL-029	gyrA	p.Glu21Gln	1.0	missense_variant	-
BPAL-029	gyrA	p.Ser95Thr	1.0	missense_variant	-
BPAL-029	gyrA	p.Ala384Val	1.0	missense_variant	-
BPAL-029	gyrA	c.1842T>C	1.0	synonymous_variar	-
BPAL-029	gyrA	c.1959G>C	1.0	synonymous_variar	-
BPAL-029	gyrA	p.Gly668Asp	1.0	missense_variant	-
BPAL-029	Rv0010c	c.384T>C	1.0	synonymous_variar	-
BPAL-029	Rv0010c	p.Ile87Met	1.0	missense_variant	-
BPAL-029	Rv0010c	c.99T>C	1.0	synonymous_variar	-
BPAL-029	fgd1	c.960T>C	1.0	synonymous_variar	-
BPAL-029	mshA	c.21T>C	1.0	synonymous_variar	-
BPAL-029	Rv0565c	c.-108T>C	1.0	upstream_gene_var	-
BPAL-029	nusG	c.-138T>C	1.0	upstream_gene_var	-
BPAL-029	rpoB	p.Phe971Leu	1.0	missense_variant	-
BPAL-029	rpoB	c.3225T>C	1.0	synonymous_variar	-
BPAL-029	rpoC	c.162G>C	1.0	synonymous_variar	-
BPAL-029	rpoC	p.Ala172Val	1.0	missense_variant	-

BPAL-029	rpoC	c.517C>A	1.0	synonymous_variar	-
BPAL-029	mmpL5	p.Ile948Val	1.0	missense_variant	-
BPAL-029	mmpL5	p.Thr794Ile	1.0	missense_variant	-
BPAL-029	rpsL	c.-165T>C	1.0	upstream_gene_var	-
BPAL-029	rplC	c.-452C>A	1.0	upstream_gene_var	-
BPAL-029	Rv1129c	c.-28T>C	1.0	upstream_gene_var	-
BPAL-029	Rv1258c	c.1029T>C	1.0	synonymous_variar	-
BPAL-029	embR	p.Phe376Leu	0.212765957446808	missense_variant	-
BPAL-029	embR	p.Cys372Gly	0.212765957446808	missense_variant	-
BPAL-029	embR	p.Cys110Tyr	1.0	missense_variant	-
BPAL-029	embR	c.-207C>G	1.0	upstream_gene_var	-
BPAL-029	embR	c.-446C>T	1.0	upstream_gene_var	-
BPAL-029	atpE	c.-138T>C	1.0	upstream_gene_var	-
BPAL-029	rrs	n.-187C>T	1.0	upstream_gene_var	-
BPAL-029	inhA	c.-40C>T	1.0	upstream_gene_var	-
BPAL-029	tsnR	c.369C>T	1.0	synonymous_variar	-
BPAL-029	tsnR	p.Leu232Pro	1.0	missense_variant	-
BPAL-029	tlyA	c.33A>G	1.0	synonymous_variar	-
BPAL-029	bacA	p.Ile603Val	1.0	missense_variant	-
BPAL-029	katG	p.Arg463Leu	1.0	missense_variant	-
BPAL-029	PPE35	p.Leu896Ser	1.0	missense_variant	-
BPAL-029	Rv1979c	p.Asp286Gly	1.0	missense_variant	-
BPAL-029	Rv1979c	c.-129A>G	1.0	upstream_gene_var	-
BPAL-029	pncA	c.-283C>T	1.0	upstream_gene_var	-
BPAL-029	kasA	c.18C>T	1.0	synonymous_variar	-
BPAL-029	kasA	p.Gly312Ser	1.0	missense_variant	-
BPAL-029	ahpC	c.-142G>A	1.0	upstream_gene_var	-
BPAL-029	ald	c.-32T>C	1.0	upstream_gene_var	-
BPAL-029	fbiD	c.300A>G	1.0	synonymous_variar	-
BPAL-029	Rv3083	p.Asp71His	1.0	missense_variant	-
BPAL-029	whiB7	c.188delG	1.0	frameshift_variant	-
BPAL-029	lpqB	p.Asp142Gly	1.0	missense_variant	-
BPAL-029	mtrB	p.Met517Leu	1.0	missense_variant	-
BPAL-029	fbiA	c.15T>C	1.0	synonymous_variar	-
BPAL-029	fbiB	c.1041C>A	1.0	synonymous_variar	-
BPAL-029	clpC1	p.Val63Ala	1.0	missense_variant	-
BPAL-029	glpK	p.Val460Ala	1.0	missense_variant	-
BPAL-029	glpK	p.Asn92Thr	0.733333333333333	missense_variant	-
BPAL-029	embC	p.Thr270Ile	1.0	missense_variant	-
BPAL-029	embC	p.Asn394Asp	1.0	missense_variant	-
BPAL-029	embC	c.2781C>T	1.0	synonymous_variar	-
BPAL-029	embA	c.348G>A	1.0	synonymous_variar	-
BPAL-029	embA	c.1188G>C	1.0	synonymous_variar	-
BPAL-029	embA	p.Pro913Ser	1.0	missense_variant	-
BPAL-029	embB	c.1065G>A	1.0	synonymous_variar	-
BPAL-029	embB	p.Glu378Ala	1.0	missense_variant	-
BPAL-029	ubiA	p.Glu149Asp	1.0	missense_variant	-
BPAL-029	ubiA	c.228T>C	1.0	synonymous_variar	-
BPAL-029	ubiA	c.-36delG	1.0	upstream_gene_var	-
BPAL-029	whiB6	p.Arg54Gln	1.0	missense_variant	-
BPAL-029	whiB6	c.-75delG	1.0	upstream_gene_var	-
BPAL-029	whiB6	c.-82C>T	1.0	upstream_gene_var	-
BPAL-029	whiB6	c.-211C>T	1.0	upstream_gene_var	-
BPAL-029	gid	c.615A>G	1.0	synonymous_variar	-
BPAL-029	gid	c.330G>T	1.0	synonymous_variar	-
BPAL-030	rpoB	p.Ser450Leu	1.0	missense_variant	rifampicin
BPAL-030	gid	c.136_137ins	1.0	frameshift_variant	streptomycin
BPAL-030	gyrA	p.Glu21Gln	1.0	missense_variant	-
BPAL-030	gyrA	p.Ser95Thr	1.0	missense_variant	-
BPAL-030	gyrA	c.313C>T	1.0	synonymous_variar	-
BPAL-030	gyrA	p.Gly668Asp	1.0	missense_variant	-
BPAL-030	rpoB	c.2700C>T	1.0	synonymous_variar	-

BPAL-030	rpoC	c.1626C>G	1.0	synonymous_variar	-
BPAL-030	mmpL5	p.Ile948Val	1.0	missense_variant	-
BPAL-030	rpsL	c.-165T>C	1.0	upstream_gene_var	-
BPAL-030	sigE	p.Arg8Trp	1.0	missense_variant	-
BPAL-030	embR	p.Phe376Leu	0.291666666666666	missense_variant	-
BPAL-030	embR	p.Cys372Gly	0.285714285714285	missense_variant	-
BPAL-030	rrs	n.-187C>T	1.0	upstream_gene_var	-
BPAL-030	tsnR	p.Leu232Pro	1.0	missense_variant	-
BPAL-030	tlyA	c.33A>G	1.0	synonymous_variar	-
BPAL-030	Rv1979c	c.-129A>G	1.0	upstream_gene_var	-
BPAL-030	ahpC	p.Pro44Arg	0.923076923076923	missense_variant	-
BPAL-030	thyA	p.Thr202Ala	1.0	missense_variant	-
BPAL-030	ald	c.-32T>C	1.0	upstream_gene_var	-
BPAL-030	Rv3236c	p.Ala370Thr	1.0	missense_variant	-
BPAL-030	mtrB	p.Met517Leu	1.0	missense_variant	-
BPAL-030	clpC1	c.2418C>T	1.0	synonymous_variar	-
BPAL-030	embC	c.2781C>T	1.0	synonymous_variar	-
BPAL-030	embB	c.54G>T	1.0	synonymous_variar	-
BPAL-030	whiB6	c.-75delG	1.0	upstream_gene_var	-
BPAL-030	whiB6	c.-211C>T	1.0	upstream_gene_var	-
BPAL-030	gid	p.Leu16Arg	1.0	missense_variant	-
BPAL-032	rpoB	p.Gln429His	1.0	missense_variant	rifampicin
BPAL-032	rpoB	p.His445Tyr	1.0	missense_variant	rifampicin
BPAL-032	katG	p.Ser315Thr	1.0	missense_variant	isoniazid
BPAL-032	embB	p.Met306Ile	0.785714285714285	missense_variant	ethambutol
BPAL-032	gid	c.98delG	1.0	frameshift_variant	streptomycin
BPAL-032	gyrB	c.-26C>T	0.470588235294117	upstream_gene_var	-
BPAL-032	gyrB	p.Met291Ile	1.0	missense_variant	-
BPAL-032	gyrA	p.Glu21Gln	1.0	missense_variant	-
BPAL-032	gyrA	p.Ser95Thr	1.0	missense_variant	-
BPAL-032	gyrA	p.Ala384Val	1.0	missense_variant	-
BPAL-032	gyrA	c.1842T>C	1.0	synonymous_variar	-
BPAL-032	gyrA	c.1959G>C	1.0	synonymous_variar	-
BPAL-032	gyrA	p.Gly668Asp	1.0	missense_variant	-
BPAL-032	Rv0010c	c.384T>C	1.0	synonymous_variar	-
BPAL-032	Rv0010c	p.Ile87Met	1.0	missense_variant	-
BPAL-032	Rv0010c	c.99T>C	1.0	synonymous_variar	-
BPAL-032	fgd1	c.960T>C	1.0	synonymous_variar	-
BPAL-032	mshA	c.21T>C	1.0	synonymous_variar	-
BPAL-032	Rv0565c	c.-108T>C	1.0	upstream_gene_var	-
BPAL-032	nusG	c.-138T>C	1.0	upstream_gene_var	-
BPAL-032	rpoB	c.3225T>C	1.0	synonymous_variar	-
BPAL-032	rpoC	c.162G>C	1.0	synonymous_variar	-
BPAL-032	rpoC	p.Ala172Val	1.0	missense_variant	-
BPAL-032	rpoC	c.517C>A	0.933333333333333	synonymous_variar	-
BPAL-032	mmpL5	p.Ile948Val	1.0	missense_variant	-
BPAL-032	mmpL5	p.Thr794Ile	1.0	missense_variant	-
BPAL-032	mmpS5	c.-593G>A	1.0	upstream_gene_var	-
BPAL-032	rpsL	c.-165T>C	1.0	upstream_gene_var	-
BPAL-032	rplC	c.-452C>A	1.0	upstream_gene_var	-
BPAL-032	Rv1129c	c.-28T>C	1.0	upstream_gene_var	-
BPAL-032	embR	p.Phe376Leu	0.285714285714285	missense_variant	-
BPAL-032	embR	p.Cys372Gly	0.277777777777777	missense_variant	-
BPAL-032	embR	p.Cys110Tyr	1.0	missense_variant	-
BPAL-032	embR	c.-207C>G	1.0	upstream_gene_var	-
BPAL-032	embR	c.-446C>T	1.0	upstream_gene_var	-
BPAL-032	atpE	c.-138T>C	1.0	upstream_gene_var	-
BPAL-032	rrs	n.-187C>T	1.0	upstream_gene_var	-
BPAL-032	rrl	n.1075A>G	1.0	non_coding_transcı	-
BPAL-032	tsnR	c.369C>T	1.0	synonymous_variar	-
BPAL-032	tlyA	c.33A>G	1.0	synonymous_variar	-
BPAL-032	bacA	p.Ile603Val	1.0	missense_variant	-

BPAL-032	katG	p.Arg463Leu	1.0	missense_variant	-
BPAL-032	PPE35	p.Leu896Ser	1.0	missense_variant	-
BPAL-032	PPE35	p.Leu189Val	0.208333333333333	missense_variant	-
BPAL-032	PPE35	p.Thr187Ser	0.208333333333333	missense_variant	-
BPAL-032	Rv1979c	p.Asp286Gly	1.0	missense_variant	-
BPAL-032	Rv1979c	c.-129A>G	1.0	upstream_gene_var	-
BPAL-032	kasA	c.18C>T	1.0	synonymous_variar	-
BPAL-032	kasA	p.Gly312Ser	1.0	missense_variant	-
BPAL-032	ahpC	c.-142G>A	1.0	upstream_gene_var	-
BPAL-032	ald	c.-32T>C	1.0	upstream_gene_var	-
BPAL-032	fbiD	c.300A>G	1.0	synonymous_variar	-
BPAL-032	Rv3083	p.Asp71His	1.0	missense_variant	-
BPAL-032	whiB7	c.188delG	1.0	frameshift_variant	-
BPAL-032	whiB7	p.Pro62Leu	1.0	missense_variant	-
BPAL-032	lpqB	p.Asp142Gly	1.0	missense_variant	-
BPAL-032	mtrB	p.Met517Leu	1.0	missense_variant	-
BPAL-032	fbiA	c.15T>C	1.0	synonymous_variar	-
BPAL-032	clpC1	p.Val63Ala	1.0	missense_variant	-
BPAL-032	glpK	p.Val460Ala	1.0	missense_variant	-
BPAL-032	embC	p.Thr270Ile	1.0	missense_variant	-
BPAL-032	embC	p.Asn394Asp	1.0	missense_variant	-
BPAL-032	embC	c.2781C>T	1.0	synonymous_variar	-
BPAL-032	embA	c.348G>A	1.0	synonymous_variar	-
BPAL-032	embA	c.1188G>C	1.0	synonymous_variar	-
BPAL-032	embA	p.Pro913Ser	1.0	missense_variant	-
BPAL-032	embB	c.1065G>A	1.0	synonymous_variar	-
BPAL-032	embB	p.Glu378Ala	1.0	missense_variant	-
BPAL-032	ubiA	p.Glu149Asp	1.0	missense_variant	-
BPAL-032	ubiA	c.228T>C	1.0	synonymous_variar	-
BPAL-032	whiB6	p.Arg54Gln	1.0	missense_variant	-
BPAL-032	whiB6	c.-75delG	1.0	upstream_gene_var	-
BPAL-032	whiB6	c.-82C>T	1.0	upstream_gene_var	-
BPAL-032	whiB6	c.-211C>T	1.0	upstream_gene_var	-
BPAL-032	gid	c.330G>T	1.0	synonymous_variar	-
BPAL-032	gid	p.Leu74Phe	1.0	missense_variant	-
BPAL-033	gyrA	p.Asp94Gly	1.0	missense_variant	levofloxacin;moxifloxacin
BPAL-033	rpoB	p.His445Asp	1.0	missense_variant	rifampicin
BPAL-033	katG	p.Ser315Thr	1.0	missense_variant	isoniazid
BPAL-033	dnaA	p.Glu323Gly	1.0	missense_variant	-
BPAL-033	gyrA	p.Glu21Gln	1.0	missense_variant	-
BPAL-033	gyrA	p.Ser95Thr	1.0	missense_variant	-
BPAL-033	gyrA	p.Gly668Asp	1.0	missense_variant	-
BPAL-033	rpoC	c.1626C>G	1.0	synonymous_variar	-
BPAL-033	mmpL5	p.Ile948Val	1.0	missense_variant	-
BPAL-033	rpsL	c.-165T>C	1.0	upstream_gene_var	-
BPAL-033	sigE	p.Arg8Trp	1.0	missense_variant	-
BPAL-033	embR	p.Phe376Leu	0.2	missense_variant	-
BPAL-033	embR	p.Cys372Gly	0.205882352941176	missense_variant	-
BPAL-033	rrs	n.-187C>T	1.0	upstream_gene_var	-
BPAL-033	tsnR	p.Leu232Pro	1.0	missense_variant	-
BPAL-033	tlyA	c.33A>G	1.0	synonymous_variar	-
BPAL-033	Rv1979c	c.-129A>G	1.0	upstream_gene_var	-
BPAL-033	ahpC	p.Pro44Arg	1.0	missense_variant	-
BPAL-033	thyA	p.Thr202Ala	1.0	missense_variant	-
BPAL-033	ald	c.-32T>C	1.0	upstream_gene_var	-
BPAL-033	Rv3236c	p.Ala370Thr	1.0	missense_variant	-
BPAL-033	lpqB	c.1458C>T	1.0	synonymous_variar	-
BPAL-033	mtrB	p.Met517Leu	1.0	missense_variant	-
BPAL-033	clpC1	c.2418C>T	1.0	synonymous_variar	-
BPAL-033	embC	c.2781C>T	1.0	synonymous_variar	-
BPAL-033	whiB6	c.-75delG	1.0	upstream_gene_var	-
BPAL-033	whiB6	c.-211C>T	1.0	upstream_gene_var	-

BPAL-033	gid	p.Leu90Phe	1.0	missense_variant	-
BPAL-033	gid	p.Leu16Arg	1.0	missense_variant	-
BPAL-036	rpoB	p.Ser450Leu	1.0	missense_variant	rifampicin
BPAL-036	katG	p.Ser315Thr	1.0	missense_variant	isoniazid
BPAL-036	dnaA	c.1302C>A	1.0	synonymous_variar	-
BPAL-036	gyrB	p.Met291Ile	1.0	missense_variant	-
BPAL-036	gyrA	p.Glu21Gln	1.0	missense_variant	-
BPAL-036	gyrA	p.Ser95Thr	1.0	missense_variant	-
BPAL-036	gyrA	p.Ala384Val	1.0	missense_variant	-
BPAL-036	gyrA	c.1842T>C	1.0	synonymous_variar	-
BPAL-036	gyrA	c.1959G>C	1.0	synonymous_variar	-
BPAL-036	gyrA	p.Gly668Asp	1.0	missense_variant	-
BPAL-036	Rv0010c	c.384T>C	1.0	synonymous_variar	-
BPAL-036	Rv0010c	p.Ile87Met	1.0	missense_variant	-
BPAL-036	Rv0010c	c.99T>C	1.0	synonymous_variar	-
BPAL-036	fgd1	c.960T>C	1.0	synonymous_variar	-
BPAL-036	mshA	c.21T>C	1.0	synonymous_variar	-
BPAL-036	Rv0565c	c.-108T>C	0.964285714285714	upstream_gene_var	-
BPAL-036	nusG	c.-138T>C	1.0	upstream_gene_var	-
BPAL-036	nusG	p.Val70Met	1.0	missense_variant	-
BPAL-036	rpoB	c.3225T>C	1.0	synonymous_variar	-
BPAL-036	rpoC	c.162G>C	1.0	synonymous_variar	-
BPAL-036	rpoC	p.Ala172Val	1.0	missense_variant	-
BPAL-036	rpoC	c.517C>A	1.0	synonymous_variar	-
BPAL-036	rpoC	p.Pro546Leu	1.0	missense_variant	-
BPAL-036	mmpL5	p.Ile948Val	1.0	missense_variant	-
BPAL-036	mmpL5	p.Thr794Ile	1.0	missense_variant	-
BPAL-036	rpsL	c.-165T>C	1.0	upstream_gene_var	-
BPAL-036	rplC	c.-452C>A	1.0	upstream_gene_var	-
BPAL-036	Rv1129c	c.-28T>C	1.0	upstream_gene_var	-
BPAL-036	Rv1258c	c.1029T>C	1.0	synonymous_variar	-
BPAL-036	embR	p.Cys110Tyr	1.0	missense_variant	-
BPAL-036	embR	c.-207C>G	1.0	upstream_gene_var	-
BPAL-036	embR	c.-446C>T	1.0	upstream_gene_var	-
BPAL-036	atpE	c.-138T>C	1.0	upstream_gene_var	-
BPAL-036	rrs	n.-187C>T	1.0	upstream_gene_var	-
BPAL-036	rrl	n.982G>A	1.0	non_coding_transcı	-
BPAL-036	inhA	c.-40C>T	1.0	upstream_gene_var	-
BPAL-036	tsnR	c.369C>T	1.0	synonymous_variar	-
BPAL-036	tsnR	p.Leu232Pro	1.0	missense_variant	-
BPAL-036	tlyA	c.33A>G	1.0	synonymous_variar	-
BPAL-036	bacA	p.Ile603Val	1.0	missense_variant	-
BPAL-036	katG	p.Leu634Ile	0.135135135135135	missense_variant	-
BPAL-036	katG	p.Asp509His	1.0	missense_variant	-
BPAL-036	katG	p.Arg463Leu	1.0	missense_variant	-
BPAL-036	PPE35	p.Leu896Ser	1.0	missense_variant	-
BPAL-036	Rv1979c	p.Asp286Gly	1.0	missense_variant	-
BPAL-036	Rv1979c	c.-129A>G	1.0	upstream_gene_var	-
BPAL-036	kasA	c.18C>T	1.0	synonymous_variar	-
BPAL-036	kasA	p.Gly312Ser	1.0	missense_variant	-
BPAL-036	ahpC	c.-142G>A	1.0	upstream_gene_var	-
BPAL-036	ald	c.-32T>C	1.0	upstream_gene_var	-
BPAL-036	fbiD	c.300A>G	1.0	synonymous_variar	-
BPAL-036	Rv3083	p.Asp71His	1.0	missense_variant	-
BPAL-036	whiB7	c.188delG	1.0	frameshift_variant	-
BPAL-036	lpqB	p.Asp142Gly	1.0	missense_variant	-
BPAL-036	mtrB	p.Met517Leu	1.0	missense_variant	-
BPAL-036	fbiA	c.15T>C	1.0	synonymous_variar	-
BPAL-036	clpC1	p.Val63Ala	1.0	missense_variant	-
BPAL-036	glpK	p.Val460Ala	1.0	missense_variant	-
BPAL-036	embC	p.Thr270Ile	1.0	missense_variant	-
BPAL-036	embC	p.Asn394Asp	1.0	missense_variant	-

BPAL-036	embC	c.2781C>T	1.0	synonymous_variar	-
BPAL-036	embA	c.348G>A	1.0	synonymous_variar	-
BPAL-036	embA	c.1188G>C	1.0	synonymous_variar	-
BPAL-036	embA	p.Pro913Ser	1.0	missense_variant	-
BPAL-036	embB	c.1065G>A	1.0	synonymous_variar	-
BPAL-036	embB	p.Glu378Ala	1.0	missense_variant	-
BPAL-036	embB	p.Ala409Pro	1.0	missense_variant	-
BPAL-036	ubiA	p.Glu149Asp	1.0	missense_variant	-
BPAL-036	ubiA	c.228T>C	1.0	synonymous_variar	-
BPAL-036	ubiA	c.-36delG	1.0	upstream_gene_var	-
BPAL-036	whiB6	p.Arg54Gln	1.0	missense_variant	-
BPAL-036	whiB6	c.-75delG	1.0	upstream_gene_var	-
BPAL-036	whiB6	c.-82C>T	1.0	upstream_gene_var	-
BPAL-036	whiB6	c.-211C>T	1.0	upstream_gene_var	-
BPAL-036	gid	c.615A>G	1.0	synonymous_variar	-
BPAL-036	gid	c.330G>T	1.0	synonymous_variar	-
BPAL-038	rpoB	p.Ser450Leu	1.0	missense_variant	rifampicin
BPAL-038	mmpR5	c.144dupC	0.152542372881355	frameshift_variant	bedaquiline;clofazimine
BPAL-038	mmpR5	c.321dupC	0.135135135135135	frameshift_variant	bedaquiline;clofazimine
BPAL-038	mmpR5	c.382dupG	0.230769230769230	frameshift_variant	bedaquiline;clofazimine
BPAL-038	inhA	c.-777C>T	1.0	upstream_gene_var	ethionamide;isoniazid
BPAL-038	gyrA	p.Glu21Gln	1.0	missense_variant	-
BPAL-038	gyrA	p.Ser95Thr	1.0	missense_variant	-
BPAL-038	gyrA	p.Gly668Asp	1.0	missense_variant	-
BPAL-038	rpoB	c.2700C>T	1.0	synonymous_variar	-
BPAL-038	rpoC	p.Ile491Val	1.0	missense_variant	-
BPAL-038	rpoC	c.1626C>G	1.0	synonymous_variar	-
BPAL-038	mmpL5	p.Ile948Val	1.0	missense_variant	-
BPAL-038	mmpR5	p.Gln115Pro	0.121212121212121	missense_variant	-
BPAL-038	rpsL	c.-165T>C	1.0	upstream_gene_var	-
BPAL-038	sigE	p.Arg8Trp	1.0	missense_variant	-
BPAL-038	embR	p.Phe376Leu	0.257575757575757	missense_variant	-
BPAL-038	embR	p.Cys372Gly	0.257575757575757	missense_variant	-
BPAL-038	embR	c.27G>A	1.0	synonymous_variar	-
BPAL-038	rrs	n.-187C>T	1.0	upstream_gene_var	-
BPAL-038	tsnR	p.Leu232Pro	1.0	missense_variant	-
BPAL-038	tlyA	c.33A>G	1.0	synonymous_variar	-
BPAL-038	Rv1979c	c.-129A>G	1.0	upstream_gene_var	-
BPAL-038	ahpC	p.Pro44Arg	1.0	missense_variant	-
BPAL-038	Rv2477c	p.Asp503Asn	1.0	missense_variant	-
BPAL-038	thyA	p.Thr202Ala	1.0	missense_variant	-
BPAL-038	ald	c.-32T>C	1.0	upstream_gene_var	-
BPAL-038	Rv3236c	p.Ala370Thr	1.0	missense_variant	-
BPAL-038	mtrB	p.Met517Leu	1.0	missense_variant	-
BPAL-038	alr	c.1074C>T	1.0	synonymous_variar	-
BPAL-038	clpC1	c.2418C>T	1.0	synonymous_variar	-
BPAL-038	embC	c.2781C>T	1.0	synonymous_variar	-
BPAL-038	whiB6	c.-75delG	1.0	upstream_gene_var	-
BPAL-038	whiB6	c.-211C>T	1.0	upstream_gene_var	-
BPAL-038	gid	p.Leu142Phe	1.0	missense_variant	-
BPAL-038	gid	p.Leu16Arg	1.0	missense_variant	-
BPAL-039	rpoB	p.Ser450Leu	1.0	missense_variant	rifampicin
BPAL-039	katG	p.Ser315Thr	1.0	missense_variant	isoniazid
BPAL-039	pncA	c.244_245ins	0.647058823529411	frameshift_variant	pyrazinamide
BPAL-039	embB	p.Met306Val	1.0	missense_variant	ethambutol
BPAL-039	gyrA	p.Glu21Gln	1.0	missense_variant	-
BPAL-039	gyrA	p.Ser95Thr	1.0	missense_variant	-
BPAL-039	gyrA	p.Gly668Asp	1.0	missense_variant	-
BPAL-039	rpoC	p.Val483Ala	1.0	missense_variant	-
BPAL-039	rpoC	c.1626C>G	1.0	synonymous_variar	-
BPAL-039	mmpL5	p.Ile948Val	1.0	missense_variant	-
BPAL-039	rpsL	c.-165T>C	1.0	upstream_gene_var	-

BPAL-039	embR	p.Phe376Leu	0.270833333333333	missense_variant	-
BPAL-039	embR	p.Cys372Gly	0.265306122448979	missense_variant	-
BPAL-039	rrs	n.-187C>T	1.0	upstream_gene_var	-
BPAL-039	inhA	c.-822C>G	0.195652173913043	upstream_gene_var	-
BPAL-039	tsnR	p.Leu232Pro	1.0	missense_variant	-
BPAL-039	tlyA	c.33A>G	1.0	synonymous_variar	-
BPAL-039	Rv1979c	c.-129A>G	1.0	upstream_gene_var	-
BPAL-039	Rv2752c	p.Val300Ala	1.0	missense_variant	-
BPAL-039	thyA	p.Thr202Ala	1.0	missense_variant	-
BPAL-039	ald	c.-32T>C	1.0	upstream_gene_var	-
BPAL-039	Rv3236c	p.Ala370Thr	1.0	missense_variant	-
BPAL-039	mtrB	p.Met517Leu	0.956521739130434	missense_variant	-
BPAL-039	clpC1	c.2418C>T	1.0	synonymous_variar	-
BPAL-039	embC	c.-100C>T	1.0	upstream_gene_var	-
BPAL-039	embC	c.2781C>T	1.0	synonymous_variar	-
BPAL-039	whiB6	c.-75delG	1.0	upstream_gene_var	-
BPAL-039	whiB6	c.-211C>T	1.0	upstream_gene_var	-
BPAL-039	gid	p.Leu16Arg	1.0	missense_variant	-
BPAL-041	rpoB	p.Ser450Leu	1.0	missense_variant	rifampicin
BPAL-041	katG	p.Ser315Thr	1.0	missense_variant	isoniazid
BPAL-041	pncA	c.152_153del	0.977272727272727	frameshift_variant	pyrazinamide
BPAL-041	embB	p.Met306Leu	1.0	missense_variant	ethambutol
BPAL-041	dnaA	c.1302C>A	1.0	synonymous_variar	-
BPAL-041	gyrB	p.Met291Ile	1.0	missense_variant	-
BPAL-041	gyrA	p.Glu21Gln	1.0	missense_variant	-
BPAL-041	gyrA	p.Ser95Thr	1.0	missense_variant	-
BPAL-041	gyrA	p.Ala384Val	1.0	missense_variant	-
BPAL-041	gyrA	c.1842T>C	1.0	synonymous_variar	-
BPAL-041	gyrA	c.1959G>C	1.0	synonymous_variar	-
BPAL-041	gyrA	p.Gly668Asp	1.0	missense_variant	-
BPAL-041	Rv0010c	c.384T>C	1.0	synonymous_variar	-
BPAL-041	Rv0010c	p.Ile87Met	1.0	missense_variant	-
BPAL-041	Rv0010c	c.99T>C	1.0	synonymous_variar	-
BPAL-041	fgd1	c.516C>A	1.0	synonymous_variar	-
BPAL-041	fgd1	c.960T>C	1.0	synonymous_variar	-
BPAL-041	mshA	c.21T>C	1.0	synonymous_variar	-
BPAL-041	Rv0565c	c.-108T>C	1.0	upstream_gene_var	-
BPAL-041	nusG	c.-138T>C	1.0	upstream_gene_var	-
BPAL-041	nusG	p.Leu73Pro	1.0	missense_variant	-
BPAL-041	rpoB	c.3225T>C	1.0	synonymous_variar	-
BPAL-041	rpoC	c.162G>C	1.0	synonymous_variar	-
BPAL-041	rpoC	p.Ala172Val	1.0	missense_variant	-
BPAL-041	rpoC	c.517C>A	1.0	synonymous_variar	-
BPAL-041	mmpL5	p.Ile948Val	1.0	missense_variant	-
BPAL-041	mmpL5	p.Thr794Ile	1.0	missense_variant	-
BPAL-041	rpsL	c.-165T>C	1.0	upstream_gene_var	-
BPAL-041	rplC	c.-452C>A	1.0	upstream_gene_var	-
BPAL-041	Rv1129c	c.-28T>C	1.0	upstream_gene_var	-
BPAL-041	Rv1258c	c.1029T>C	1.0	synonymous_variar	-
BPAL-041	Rv1258c	c.354G>A	1.0	synonymous_variar	-
BPAL-041	embR	p.Cys110Tyr	1.0	missense_variant	-
BPAL-041	embR	c.-207C>G	1.0	upstream_gene_var	-
BPAL-041	embR	c.-446C>T	1.0	upstream_gene_var	-
BPAL-041	atpE	c.-138T>C	0.983333333333333	upstream_gene_var	-
BPAL-041	rrs	n.-187C>T	1.0	upstream_gene_var	-
BPAL-041	rrl	n.982G>A	1.0	non_coding_transcı	-
BPAL-041	inhA	c.-40C>T	0.977777777777777	upstream_gene_var	-
BPAL-041	tsnR	c.369C>T	1.0	synonymous_variar	-
BPAL-041	tsnR	p.Leu232Pro	1.0	missense_variant	-
BPAL-041	tlyA	c.33A>G	1.0	synonymous_variar	-
BPAL-041	bacA	p.Ile603Val	1.0	missense_variant	-
BPAL-041	katG	p.Leu634Ile	0.105263157894736	missense_variant	-

BPAL-041	katG	p.Arg463Leu	1.0	missense_variant	-
BPAL-041	PPE35	p.Leu896Ser	1.0	missense_variant	-
BPAL-041	PPE35	p.Leu189Val	0.194444444444444	missense_variant	-
BPAL-041	PPE35	p.Thr187Ser	0.2	missense_variant	-
BPAL-041	Rv1979c	p.Asp286Gly	1.0	missense_variant	-
BPAL-041	Rv1979c	c.-129A>G	1.0	upstream_gene_var	-
BPAL-041	kasA	c.18C>T	1.0	synonymous_variar	-
BPAL-041	kasA	p.Gly312Ser	1.0	missense_variant	-
BPAL-041	ahpC	c.-142G>A	1.0	upstream_gene_var	-
BPAL-041	ald	c.-32T>C	1.0	upstream_gene_var	-
BPAL-041	fbiD	c.300A>G	1.0	synonymous_variar	-
BPAL-041	Rv3083	p.Asp71His	1.0	missense_variant	-
BPAL-041	whiB7	c.188delG	1.0	frameshift_variant	-
BPAL-041	lpqB	p.Asp142Gly	1.0	missense_variant	-
BPAL-041	mtrB	p.Met517Leu	0.971428571428571	missense_variant	-
BPAL-041	fbiA	c.-329C>T	1.0	upstream_gene_var	-
BPAL-041	fbiA	c.15T>C	1.0	synonymous_variar	-
BPAL-041	rpoA	c.-37C>T	1.0	upstream_gene_var	-
BPAL-041	clpC1	p.Val63Ala	0.977272727272727	missense_variant	-
BPAL-041	glpK	p.Val460Ala	1.0	missense_variant	-
BPAL-041	embC	p.Thr270Ile	1.0	missense_variant	-
BPAL-041	embC	p.Asn394Asp	1.0	missense_variant	-
BPAL-041	embC	c.2781C>T	1.0	synonymous_variar	-
BPAL-041	embA	c.348G>A	1.0	synonymous_variar	-
BPAL-041	embA	c.1188G>C	1.0	synonymous_variar	-
BPAL-041	embA	p.Pro913Ser	1.0	missense_variant	-
BPAL-041	embB	c.1065G>A	1.0	synonymous_variar	-
BPAL-041	embB	p.Glu378Ala	1.0	missense_variant	-
BPAL-041	ubiA	p.Glu149Asp	1.0	missense_variant	-
BPAL-041	ubiA	c.228T>C	1.0	synonymous_variar	-
BPAL-041	ubiA	c.-36delG	1.0	upstream_gene_var	-
BPAL-041	whiB6	p.Arg54Gln	1.0	missense_variant	-
BPAL-041	whiB6	c.-75delG	1.0	upstream_gene_var	-
BPAL-041	whiB6	c.-82C>T	1.0	upstream_gene_var	-
BPAL-041	whiB6	c.-211C>T	1.0	upstream_gene_var	-
BPAL-041	gid	c.615A>G	1.0	synonymous_variar	-
BPAL-041	gid	c.330G>T	1.0	synonymous_variar	-
BPAL-041	gid	c.-163C>T	1.0	upstream_gene_var	-
BPAL-045	gyrA	p.Ala90Val	0.269230769230769	missense_variant	levofloxacin;moxifloxacin
BPAL-045	gyrA	p.Asp94Gly	0.769230769230769	missense_variant	levofloxacin;moxifloxacin
BPAL-045	rpoB	p.Ser450Leu	1.0	missense_variant	rifampicin
BPAL-045	rrs	n.514A>C	1.0	non_coding_transcı	streptomycin
BPAL-045	katG	p.Ser315Thr	1.0	missense_variant	isoniazid
BPAL-045	pncA	p.Trp68Arg	1.0	missense_variant	pyrazinamide
BPAL-045	embB	p.Met306Val	1.0	missense_variant	ethambutol
BPAL-045	gyrA	p.Glu21Gln	1.0	missense_variant	-
BPAL-045	gyrA	p.Ser95Thr	1.0	missense_variant	-
BPAL-045	gyrA	p.Gly668Asp	1.0	missense_variant	-
BPAL-045	mshA	p.Ala343Val	1.0	missense_variant	-
BPAL-045	rpoB	c.2700C>T	1.0	synonymous_variar	-
BPAL-045	rpoC	c.1626C>G	1.0	synonymous_variar	-
BPAL-045	rpoC	p.Pro1040Arg	1.0	missense_variant	-
BPAL-045	mmpL5	p.Ile948Val	1.0	missense_variant	-
BPAL-045	rpsL	c.-165T>C	1.0	upstream_gene_var	-
BPAL-045	sigE	p.Arg8Trp	1.0	missense_variant	-
BPAL-045	embR	p.Phe376Leu	0.157894736842105	missense_variant	-
BPAL-045	embR	p.Cys372Gly	0.162162162162162	missense_variant	-
BPAL-045	rrs	n.-187C>T	1.0	upstream_gene_var	-
BPAL-045	tsnR	p.Leu232Pro	1.0	missense_variant	-
BPAL-045	tlyA	c.33A>G	1.0	synonymous_variar	-
BPAL-045	Rv1979c	c.-129A>G	1.0	upstream_gene_var	-
BPAL-045	ahpC	p.Pro44Arg	1.0	missense_variant	-

BPAL-045	thyA	p.Thr202Ala	1.0	missense_variant	-
BPAL-045	ald	c.-32T>C	1.0	upstream_gene_var	-
BPAL-045	fbiD	p.Glu127Gln	1.0	missense_variant	-
BPAL-045	Rv3236c	p.Ala370Thr	1.0	missense_variant	-
BPAL-045	mtrB	p.Met517Leu	1.0	missense_variant	-
BPAL-045	fbiA	c.-300T>C	1.0	upstream_gene_var	-
BPAL-045	clpC1	c.2418C>T	1.0	synonymous_variar	-
BPAL-045	embC	c.2781C>T	1.0	synonymous_variar	-
BPAL-045	embB	p.Leu466Ser	1.0	missense_variant	-
BPAL-045	whiB6	c.-75delG	1.0	upstream_gene_var	-
BPAL-045	whiB6	c.-211C>T	1.0	upstream_gene_var	-
BPAL-045	gid	p.Leu16Arg	1.0	missense_variant	-
BPAL-047	rpoB	p.Ser450Leu	1.0	missense_variant	rifampicin
BPAL-047	katG	p.Ser315Thr	1.0	missense_variant	isoniazid
BPAL-047	pncA	c.-11A>G	1.0	upstream_gene_var	pyrazinamide
BPAL-047	embB	p.Gly406Ser	1.0	missense_variant	ethambutol
BPAL-047	dnaA	c.1302C>A	1.0	synonymous_variar	-
BPAL-047	gyrB	p.Met291Ile	1.0	missense_variant	-
BPAL-047	gyrA	p.Glu21Gln	1.0	missense_variant	-
BPAL-047	gyrA	p.Ser95Thr	1.0	missense_variant	-
BPAL-047	gyrA	p.Ala384Val	1.0	missense_variant	-
BPAL-047	gyrA	c.1842T>C	1.0	synonymous_variar	-
BPAL-047	gyrA	c.1959G>C	1.0	synonymous_variar	-
BPAL-047	gyrA	p.Gly668Asp	1.0	missense_variant	-
BPAL-047	Rv0010c	c.384T>C	1.0	synonymous_variar	-
BPAL-047	Rv0010c	p.Ile87Met	1.0	missense_variant	-
BPAL-047	Rv0010c	c.99T>C	1.0	synonymous_variar	-
BPAL-047	fgd1	c.960T>C	1.0	synonymous_variar	-
BPAL-047	mshA	c.21T>C	1.0	synonymous_variar	-
BPAL-047	Rv0565c	c.-108T>C	1.0	upstream_gene_var	-
BPAL-047	nusG	c.-138T>C	1.0	upstream_gene_var	-
BPAL-047	rpoB	c.3225T>C	1.0	synonymous_variar	-
BPAL-047	rpoC	c.162G>C	1.0	synonymous_variar	-
BPAL-047	rpoC	p.Ala172Val	1.0	missense_variant	-
BPAL-047	rpoC	c.517C>A	1.0	synonymous_variar	-
BPAL-047	rpoC	p.Met983Ile	1.0	missense_variant	-
BPAL-047	mmpL5	p.Ile948Val	1.0	missense_variant	-
BPAL-047	mmpL5	p.Thr794Ile	1.0	missense_variant	-
BPAL-047	rpsL	c.-165T>C	1.0	upstream_gene_var	-
BPAL-047	rplC	c.-452C>A	1.0	upstream_gene_var	-
BPAL-047	Rv1129c	c.-28T>C	1.0	upstream_gene_var	-
BPAL-047	Rv1258c	c.1029T>C	1.0	synonymous_variar	-
BPAL-047	embR	p.Cys110Tyr	1.0	missense_variant	-
BPAL-047	embR	c.-207C>G	1.0	upstream_gene_var	-
BPAL-047	embR	c.-446C>T	1.0	upstream_gene_var	-
BPAL-047	atpE	c.-138T>C	1.0	upstream_gene_var	-
BPAL-047	rrs	n.-187C>T	0.956521739130434	upstream_gene_var	-
BPAL-047	rrl	n.982G>A	1.0	non_coding_transcr	-
BPAL-047	inhA	c.-40C>T	1.0	upstream_gene_var	-
BPAL-047	tsnR	c.369C>T	1.0	synonymous_variar	-
BPAL-047	tsnR	p.Leu232Pro	1.0	missense_variant	-
BPAL-047	tlyA	c.33A>G	1.0	synonymous_variar	-
BPAL-047	bacA	p.Ile603Val	1.0	missense_variant	-
BPAL-047	katG	p.Arg463Leu	1.0	missense_variant	-
BPAL-047	PPE35	p.Leu896Ser	1.0	missense_variant	-
BPAL-047	Rv1979c	p.Asp286Gly	1.0	missense_variant	-
BPAL-047	Rv1979c	c.-129A>G	1.0	upstream_gene_var	-
BPAL-047	kasA	c.18C>T	1.0	synonymous_variar	-
BPAL-047	kasA	p.Gly312Ser	1.0	missense_variant	-
BPAL-047	ahpC	c.-142G>A	1.0	upstream_gene_var	-
BPAL-047	ald	c.-32T>C	1.0	upstream_gene_var	-
BPAL-047	fbiD	c.300A>G	0.941176470588235	synonymous_variar	-

BPAL-047	Rv3083	p.Asp71His	1.0	missense_variant	-
BPAL-047	whiB7	c.188delG	1.0	frameshift_variant	-
BPAL-047	lpqB	p.Asp142Gly	1.0	missense_variant	-
BPAL-047	mtrB	p.Met517Leu	1.0	missense_variant	-
BPAL-047	fbiA	c.15T>C	1.0	synonymous_variar	-
BPAL-047	clpC1	p.Val63Ala	1.0	missense_variant	-
BPAL-047	glpK	p.Val460Ala	1.0	missense_variant	-
BPAL-047	embC	p.Thr270Ile	1.0	missense_variant	-
BPAL-047	embC	p.Asn394Asp	1.0	missense_variant	-
BPAL-047	embC	c.2781C>T	1.0	synonymous_variar	-
BPAL-047	embA	c.348G>A	1.0	synonymous_variar	-
BPAL-047	embA	c.1188G>C	1.0	synonymous_variar	-
BPAL-047	embA	p.Pro913Ser	1.0	missense_variant	-
BPAL-047	embB	c.1065G>A	1.0	synonymous_variar	-
BPAL-047	embB	p.Glu378Ala	1.0	missense_variant	-
BPAL-047	ubiA	p.Glu149Asp	1.0	missense_variant	-
BPAL-047	ubiA	c.228T>C	1.0	synonymous_variar	-
BPAL-047	ubiA	c.-36delG	1.0	upstream_gene_var	-
BPAL-047	whiB6	p.Arg54Gln	1.0	missense_variant	-
BPAL-047	whiB6	c.-75delG	1.0	upstream_gene_var	-
BPAL-047	whiB6	c.-82C>T	1.0	upstream_gene_var	-
BPAL-047	whiB6	c.-211C>T	1.0	upstream_gene_var	-
BPAL-047	gid	c.615A>G	1.0	synonymous_variar	-
BPAL-047	gid	c.330G>T	1.0	synonymous_variar	-
BPAL-048	rpoB	p.Ser450Leu	1.0	missense_variant	rifampicin
BPAL-048	katG	p.Ser315Thr	1.0	missense_variant	isoniazid
BPAL-048	pncA	c.304_305ins	1.0	frameshift_variant	pyrazinamide
BPAL-048	embB	p.Met306Ile	1.0	missense_variant	ethambutol
BPAL-048	dnaA	c.1302C>A	1.0	synonymous_variar	-
BPAL-048	gyrB	p.Met291Ile	1.0	missense_variant	-
BPAL-048	gyrB	p.Phe475Leu	0.3	missense_variant	-
BPAL-048	gyrA	p.Glu21Gln	1.0	missense_variant	-
BPAL-048	gyrA	p.Ser95Thr	1.0	missense_variant	-
BPAL-048	gyrA	p.Ala384Val	1.0	missense_variant	-
BPAL-048	gyrA	c.1842T>C	1.0	synonymous_variar	-
BPAL-048	gyrA	c.1959G>C	1.0	synonymous_variar	-
BPAL-048	gyrA	p.Gly668Asp	1.0	missense_variant	-
BPAL-048	Rv0010c	c.384T>C	1.0	synonymous_variar	-
BPAL-048	Rv0010c	p.Ile87Met	1.0	missense_variant	-
BPAL-048	Rv0010c	c.99T>C	1.0	synonymous_variar	-
BPAL-048	fgd1	c.960T>C	1.0	synonymous_variar	-
BPAL-048	mshA	c.21T>C	1.0	synonymous_variar	-
BPAL-048	Rv0565c	c.-108T>C	1.0	upstream_gene_var	-
BPAL-048	nusG	c.-138T>C	1.0	upstream_gene_var	-
BPAL-048	rpoB	p.Arg552His	1.0	missense_variant	-
BPAL-048	rpoB	c.3225T>C	1.0	synonymous_variar	-
BPAL-048	rpoC	c.162G>C	1.0	synonymous_variar	-
BPAL-048	rpoC	p.Ala172Val	1.0	missense_variant	-
BPAL-048	rpoC	c.517C>A	1.0	synonymous_variar	-
BPAL-048	mmpL5	p.Ile948Val	1.0	missense_variant	-
BPAL-048	mmpL5	p.Thr794Ile	1.0	missense_variant	-
BPAL-048	rpsL	c.-165T>C	1.0	upstream_gene_var	-
BPAL-048	rplC	c.-452C>A	1.0	upstream_gene_var	-
BPAL-048	Rv1129c	c.-28T>C	1.0	upstream_gene_var	-
BPAL-048	Rv1258c	c.1029T>C	1.0	synonymous_variar	-
BPAL-048	embR	p.Cys110Tyr	1.0	missense_variant	-
BPAL-048	embR	c.-207C>G	1.0	upstream_gene_var	-
BPAL-048	atpE	c.-138T>C	1.0	upstream_gene_var	-
BPAL-048	rrs	n.-187C>T	1.0	upstream_gene_var	-
BPAL-048	inhA	c.-40C>T	1.0	upstream_gene_var	-
BPAL-048	tsnR	c.369C>T	1.0	synonymous_variar	-
BPAL-048	tsnR	p.Leu232Pro	1.0	missense_variant	-

BPAL-048	tlyA	c.33A>G	1.0	synonymous_variar	-
BPAL-048	bacA	p.Ile603Val	1.0	missense_variant	-
BPAL-048	PPE35	p.Leu896Ser	1.0	missense_variant	-
BPAL-048	Rv1979c	p.Asp286Gly	1.0	missense_variant	-
BPAL-048	Rv1979c	c.-129A>G	1.0	upstream_gene_var	-
BPAL-048	pncA	c.-283C>T	1.0	upstream_gene_var	-
BPAL-048	kasA	c.18C>T	1.0	synonymous_variar	-
BPAL-048	kasA	p.Gly312Ser	1.0	missense_variant	-
BPAL-048	ahpC	c.-142G>A	1.0	upstream_gene_var	-
BPAL-048	ald	c.-32T>C	1.0	upstream_gene_var	-
BPAL-048	ald	c.219_228del	1.0	frameshift_variant	-
BPAL-048	fbiD	c.300A>G	1.0	synonymous_variar	-
BPAL-048	Rv3083	p.Asp71His	1.0	missense_variant	-
BPAL-048	whiB7	c.188delG	1.0	frameshift_variant	-
BPAL-048	lpqB	p.Asp142Gly	1.0	missense_variant	-
BPAL-048	fbiA	c.15T>C	1.0	synonymous_variar	-
BPAL-048	fbiB	c.1041C>A	1.0	synonymous_variar	-
BPAL-048	clpC1	p.Val63Ala	1.0	missense_variant	-
BPAL-048	glpK	p.Val460Ala	1.0	missense_variant	-
BPAL-048	embC	p.Thr270Ile	1.0	missense_variant	-
BPAL-048	embC	p.Asn394Asp	1.0	missense_variant	-
BPAL-048	embC	c.2781C>T	1.0	synonymous_variar	-
BPAL-048	embA	c.348G>A	1.0	synonymous_variar	-
BPAL-048	embA	c.1188G>C	1.0	synonymous_variar	-
BPAL-048	embA	p.Pro913Ser	1.0	missense_variant	-
BPAL-048	embB	c.1065G>A	1.0	synonymous_variar	-
BPAL-048	embB	p.Glu378Ala	1.0	missense_variant	-
BPAL-048	ubiA	p.Glu149Asp	1.0	missense_variant	-
BPAL-048	ubiA	c.228T>C	1.0	synonymous_variar	-
BPAL-048	ubiA	c.-36delG	1.0	upstream_gene_var	-
BPAL-048	whiB6	p.Arg54Gln	1.0	missense_variant	-
BPAL-048	whiB6	c.-75delG	1.0	upstream_gene_var	-
BPAL-048	whiB6	c.-82C>T	1.0	upstream_gene_var	-
BPAL-048	whiB6	c.-211C>T	1.0	upstream_gene_var	-
BPAL-048	gid	c.615A>G	1.0	synonymous_variar	-
BPAL-048	gid	c.330G>T	1.0	synonymous_variar	-
BPAL-049	rpoB	p.Leu430Pro	1.0	missense_variant	rifampicin
BPAL-049	katG	p.Ser315Thr	1.0	missense_variant	isoniazid
BPAL-049	gyrA	p.Glu21Gln	1.0	missense_variant	-
BPAL-049	gyrA	p.Ser95Thr	1.0	missense_variant	-
BPAL-049	gyrA	p.Gly668Asp	1.0	missense_variant	-
BPAL-049	rpoC	c.1626C>G	1.0	synonymous_variar	-
BPAL-049	mmpL5	p.Ile948Val	1.0	missense_variant	-
BPAL-049	rpsL	c.-165T>C	1.0	upstream_gene_var	-
BPAL-049	sigE	p.Arg8Trp	0.979166666666666	missense_variant	-
BPAL-049	rrs	n.-187C>T	1.0	upstream_gene_var	-
BPAL-049	rrs	n.92C>A	1.0	non_coding_transcr	-
BPAL-049	tsnR	p.Leu232Pro	1.0	missense_variant	-
BPAL-049	tlyA	c.33A>G	1.0	synonymous_variar	-
BPAL-049	Rv1979c	c.-129A>G	1.0	upstream_gene_var	-
BPAL-049	folC	p.Val256Gly	0.102564102564102	missense_variant	-
BPAL-049	thyA	p.Thr202Ala	0.982142857142857	missense_variant	-
BPAL-049	ald	c.-32T>C	1.0	upstream_gene_var	-
BPAL-049	fbiD	c.15G>A	1.0	synonymous_variar	-
BPAL-049	Rv3236c	p.Ala370Thr	1.0	missense_variant	-
BPAL-049	mtrB	p.Met517Leu	1.0	missense_variant	-
BPAL-049	alr	p.Val210Gly	1.0	missense_variant	-
BPAL-049	clpC1	c.2418C>T	1.0	synonymous_variar	-
BPAL-049	embC	c.2781C>T	1.0	synonymous_variar	-
BPAL-049	ubiA	c.489C>T	1.0	synonymous_variar	-
BPAL-049	whiB6	c.-75delG	1.0	upstream_gene_var	-
BPAL-049	whiB6	c.-211C>T	1.0	upstream_gene_var	-

BPAL-049	gid	p.Ser181Pro	1.0	missense_variant	-
BPAL-049	gid	p.Leu16Arg	1.0	missense_variant	-
BPAL-053	rpoB	p.His445Cys	1.0	missense_variant	rifampicin
BPAL-053	katG	p.Ser315Thr	1.0	missense_variant	isoniazid
BPAL-053	dnaA	p.Asp142Asn	1.0	missense_variant	-
BPAL-053	dnaA	c.939C>A	0.995169082125603	synonymous_variar	-
BPAL-053	dnaA	c.1302C>A	1.0	synonymous_variar	-
BPAL-053	gyrB	p.Met291Ile	1.0	missense_variant	-
BPAL-053	gyrA	p.Glu21Gln	1.0	missense_variant	-
BPAL-053	gyrA	p.Ser95Thr	1.0	missense_variant	-
BPAL-053	gyrA	p.Ala384Val	1.0	missense_variant	-
BPAL-053	gyrA	c.1842T>C	1.0	synonymous_variar	-
BPAL-053	gyrA	c.1959G>C	1.0	synonymous_variar	-
BPAL-053	gyrA	p.Gly668Asp	1.0	missense_variant	-
BPAL-053	Rv0010c	c.384T>C	1.0	synonymous_variar	-
BPAL-053	Rv0010c	p.Ile87Met	1.0	missense_variant	-
BPAL-053	Rv0010c	c.99T>C	1.0	synonymous_variar	-
BPAL-053	fgd1	c.960T>C	1.0	synonymous_variar	-
BPAL-053	mshA	c.21T>C	1.0	synonymous_variar	-
BPAL-053	Rv0565c	c.-108T>C	1.0	upstream_gene_var	-
BPAL-053	nusG	c.-138T>C	1.0	upstream_gene_var	-
BPAL-053	rpoB	c.3225T>C	1.0	synonymous_variar	-
BPAL-053	rpoC	c.162G>C	1.0	synonymous_variar	-
BPAL-053	rpoC	p.Ala172Val	1.0	missense_variant	-
BPAL-053	rpoC	c.517C>A	1.0	synonymous_variar	-
BPAL-053	rpoC	c.1440C>T	1.0	synonymous_variar	-
BPAL-053	mmpL5	p.Ile948Val	1.0	missense_variant	-
BPAL-053	mmpL5	p.Thr794Ile	1.0	missense_variant	-
BPAL-053	rpsL	c.-165T>C	1.0	upstream_gene_var	-
BPAL-053	rplC	c.-452C>A	1.0	upstream_gene_var	-
BPAL-053	Rv1129c	c.-28T>C	1.0	upstream_gene_var	-
BPAL-053	fbiC	c.-110G>A	1.0	upstream_gene_var	-
BPAL-053	Rv1258c	c.1029T>C	1.0	synonymous_variar	-
BPAL-053	embR	p.Phe376Leu	0.266666666666666	missense_variant	-
BPAL-053	embR	p.Cys372Gly	0.272727272727272	missense_variant	-
BPAL-053	embR	p.Cys110Tyr	1.0	missense_variant	-
BPAL-053	embR	c.-207C>G	1.0	upstream_gene_var	-
BPAL-053	embR	c.-446C>T	1.0	upstream_gene_var	-
BPAL-053	atpE	c.-138T>C	1.0	upstream_gene_var	-
BPAL-053	rrs	n.-187C>T	1.0	upstream_gene_var	-
BPAL-053	inhA	c.-40C>T	1.0	upstream_gene_var	-
BPAL-053	tsnR	c.369C>T	1.0	synonymous_variar	-
BPAL-053	tsnR	p.Leu232Pro	1.0	missense_variant	-
BPAL-053	tlyA	c.33A>G	1.0	synonymous_variar	-
BPAL-053	bacA	p.Ile603Val	1.0	missense_variant	-
BPAL-053	katG	p.Arg463Leu	1.0	missense_variant	-
BPAL-053	PPE35	p.Leu896Ser	1.0	missense_variant	-
BPAL-053	Rv1979c	p.Asp286Gly	1.0	missense_variant	-
BPAL-053	Rv1979c	c.-129A>G	1.0	upstream_gene_var	-
BPAL-053	kasA	c.18C>T	1.0	synonymous_variar	-
BPAL-053	kasA	p.Gly312Ser	1.0	missense_variant	-
BPAL-053	ahpC	c.-142G>A	1.0	upstream_gene_var	-
BPAL-053	ribD	p.His105Gln	1.0	missense_variant	-
BPAL-053	ald	c.-32T>C	1.0	upstream_gene_var	-
BPAL-053	fbiD	c.300A>G	1.0	synonymous_variar	-
BPAL-053	Rv3083	p.Asp71His	1.0	missense_variant	-
BPAL-053	whiB7	c.188delG	1.0	frameshift_variant	-
BPAL-053	lpqB	p.Asp142Gly	1.0	missense_variant	-
BPAL-053	mtrB	p.Met517Leu	1.0	missense_variant	-
BPAL-053	fbiA	c.15T>C	1.0	synonymous_variar	-
BPAL-053	clpC1	c.2004G>A	1.0	synonymous_variar	-
BPAL-053	clpC1	p.Val63Ala	1.0	missense_variant	-

BPAL-053	clpC1	c.-192C>A	1.0	upstream_gene_var	-
BPAL-053	glpK	p.Val460Ala	1.0	missense_variant	-
BPAL-053	embC	p.Thr270Ile	1.0	missense_variant	-
BPAL-053	embC	p.Asn394Asp	1.0	missense_variant	-
BPAL-053	embC	c.2781C>T	1.0	synonymous_variar	-
BPAL-053	embA	c.348G>A	1.0	synonymous_variar	-
BPAL-053	embA	c.1188G>C	1.0	synonymous_variar	-
BPAL-053	embA	p.Pro913Ser	1.0	missense_variant	-
BPAL-053	embB	c.1065G>A	1.0	synonymous_variar	-
BPAL-053	embB	p.Glu378Ala	1.0	missense_variant	-
BPAL-053	ubiA	p.Glu149Asp	1.0	missense_variant	-
BPAL-053	ubiA	c.228T>C	1.0	synonymous_variar	-
BPAL-053	ubiA	c.-36delG	1.0	upstream_gene_var	-
BPAL-053	whiB6	p.Arg54Gln	1.0	missense_variant	-
BPAL-053	whiB6	c.-75delG	1.0	upstream_gene_var	-
BPAL-053	whiB6	c.-82C>T	1.0	upstream_gene_var	-
BPAL-053	whiB6	c.-211C>T	1.0	upstream_gene_var	-
BPAL-053	gid	c.615A>G	1.0	synonymous_variar	-
BPAL-053	gid	c.330G>T	1.0	synonymous_variar	-
BPAL-054	rpoB	p.His445Asn	1.0	missense_variant	rifampicin
BPAL-054	inhA	c.-770T>G	1.0	upstream_gene_var	ethionamide;isoniazid
BPAL-054	katG	p.Ser315Thr	1.0	missense_variant	isoniazid
BPAL-054	gid	p.Glu92*	0.982758620689655	stop_gained	streptomycin
BPAL-054	gyrB	p.Met291Ile	1.0	missense_variant	-
BPAL-054	gyrA	p.Glu21Gln	1.0	missense_variant	-
BPAL-054	gyrA	p.Ser95Thr	1.0	missense_variant	-
BPAL-054	gyrA	p.Ala384Val	1.0	missense_variant	-
BPAL-054	gyrA	c.1665C>G	1.0	synonymous_variar	-
BPAL-054	gyrA	c.1842T>C	1.0	synonymous_variar	-
BPAL-054	gyrA	c.1959G>C	1.0	synonymous_variar	-
BPAL-054	gyrA	p.Gly668Asp	1.0	missense_variant	-
BPAL-054	Rv0010c	c.384T>C	1.0	synonymous_variar	-
BPAL-054	Rv0010c	p.Ile87Met	1.0	missense_variant	-
BPAL-054	Rv0010c	c.99T>C	1.0	synonymous_variar	-
BPAL-054	fgd1	c.960T>C	1.0	synonymous_variar	-
BPAL-054	mshA	c.21T>C	1.0	synonymous_variar	-
BPAL-054	Rv0565c	c.-108T>C	1.0	upstream_gene_var	-
BPAL-054	nusG	c.-138T>C	1.0	upstream_gene_var	-
BPAL-054	rpoB	c.3225T>C	1.0	synonymous_variar	-
BPAL-054	rpoC	c.162G>C	1.0	synonymous_variar	-
BPAL-054	rpoC	p.Ala172Val	1.0	missense_variant	-
BPAL-054	rpoC	c.517C>A	1.0	synonymous_variar	-
BPAL-054	mmpL5	p.Ile948Val	1.0	missense_variant	-
BPAL-054	mmpL5	p.Thr794Ile	1.0	missense_variant	-
BPAL-054	rpsL	c.-165T>C	1.0	upstream_gene_var	-
BPAL-054	rplC	c.-452C>A	1.0	upstream_gene_var	-
BPAL-054	Rv1129c	c.-28T>C	1.0	upstream_gene_var	-
BPAL-054	embR	p.Cys110Tyr	1.0	missense_variant	-
BPAL-054	embR	c.-207C>G	1.0	upstream_gene_var	-
BPAL-054	embR	c.-446C>T	1.0	upstream_gene_var	-
BPAL-054	atpE	c.-138T>C	1.0	upstream_gene_var	-
BPAL-054	rrs	n.-187C>T	1.0	upstream_gene_var	-
BPAL-054	rrl	n.1075A>G	1.0	non_coding_transcı	-
BPAL-054	tsnR	c.369C>T	1.0	synonymous_variar	-
BPAL-054	tsnR	p.Leu232Pro	1.0	missense_variant	-
BPAL-054	tlyA	c.33A>G	1.0	synonymous_variar	-
BPAL-054	bacA	p.Ile603Val	1.0	missense_variant	-
BPAL-054	katG	p.Arg463Leu	1.0	missense_variant	-
BPAL-054	PPE35	p.Leu896Ser	1.0	missense_variant	-
BPAL-054	Rv1979c	p.Asp286Gly	1.0	missense_variant	-
BPAL-054	Rv1979c	c.-129A>G	1.0	upstream_gene_var	-
BPAL-054	kasA	c.18C>T	1.0	synonymous_variar	-

BPAL-054	kasA	c.420G>A	1.0	synonymous_variar	-
BPAL-054	kasA	p.Gly312Ser	1.0	missense_variant	-
BPAL-054	ahpC	c.-142G>A	1.0	upstream_gene_var	-
BPAL-054	ald	c.-32T>C	1.0	upstream_gene_var	-
BPAL-054	fbiD	c.300A>G	1.0	synonymous_variar	-
BPAL-054	Rv3083	p.Asp71His	1.0	missense_variant	-
BPAL-054	whiB7	c.188delG	1.0	frameshift_variant	-
BPAL-054	lpqB	p.Asp142Gly	1.0	missense_variant	-
BPAL-054	mtrB	p.Met517Leu	1.0	missense_variant	-
BPAL-054	fbiA	c.15T>C	1.0	synonymous_variar	-
BPAL-054	clpC1	p.Val63Ala	1.0	missense_variant	-
BPAL-054	glpK	p.Val460Ala	1.0	missense_variant	-
BPAL-054	glpK	c.565_566ins	0.3	frameshift_variant	-
BPAL-054	embC	p.Thr270Ile	0.978723404255319	missense_variant	-
BPAL-054	embC	p.Asn394Asp	1.0	missense_variant	-
BPAL-054	embC	c.2781C>T	1.0	synonymous_variar	-
BPAL-054	embA	c.348G>A	1.0	synonymous_variar	-
BPAL-054	embA	c.1188G>C	1.0	synonymous_variar	-
BPAL-054	embA	p.Pro913Ser	1.0	missense_variant	-
BPAL-054	embB	c.1065G>A	1.0	synonymous_variar	-
BPAL-054	embB	p.Glu378Ala	1.0	missense_variant	-
BPAL-054	ubiA	p.Glu149Asp	1.0	missense_variant	-
BPAL-054	ubiA	c.228T>C	1.0	synonymous_variar	-
BPAL-054	whiB6	p.Arg54Gln	1.0	missense_variant	-
BPAL-054	whiB6	c.-75delG	1.0	upstream_gene_var	-
BPAL-054	whiB6	c.-82C>T	1.0	upstream_gene_var	-
BPAL-054	whiB6	c.-211C>T	1.0	upstream_gene_var	-
BPAL-054	gid	c.615A>G	1.0	synonymous_variar	-
BPAL-054	gid	c.330G>T	1.0	synonymous_variar	-
BPAL-055	rpoB	p.Ser450Leu	1.0	missense_variant	rifampicin
BPAL-055	inhA	c.-777C>T	1.0	upstream_gene_var	ethionamide;isoniazid
BPAL-055	embB	p.Gly406Ser	1.0	missense_variant	ethambutol
BPAL-055	dnaA	c.1302C>A	1.0	synonymous_variar	-
BPAL-055	gyrB	p.Met291Ile	1.0	missense_variant	-
BPAL-055	gyrA	p.Glu21Gln	1.0	missense_variant	-
BPAL-055	gyrA	p.Ser95Thr	1.0	missense_variant	-
BPAL-055	gyrA	p.Ala384Val	1.0	missense_variant	-
BPAL-055	gyrA	c.1842T>C	1.0	synonymous_variar	-
BPAL-055	gyrA	c.1959G>C	1.0	synonymous_variar	-
BPAL-055	gyrA	p.Gly668Asp	1.0	missense_variant	-
BPAL-055	Rv0010c	c.384T>C	1.0	synonymous_variar	-
BPAL-055	Rv0010c	p.Ile87Met	1.0	missense_variant	-
BPAL-055	Rv0010c	c.99T>C	1.0	synonymous_variar	-
BPAL-055	fgd1	c.960T>C	1.0	synonymous_variar	-
BPAL-055	mshA	c.21T>C	1.0	synonymous_variar	-
BPAL-055	Rv0565c	c.-108T>C	1.0	upstream_gene_var	-
BPAL-055	nusG	c.-138T>C	1.0	upstream_gene_var	-
BPAL-055	rpoB	c.3225T>C	1.0	synonymous_variar	-
BPAL-055	rpoC	c.162G>C	1.0	synonymous_variar	-
BPAL-055	rpoC	p.Ala172Val	1.0	missense_variant	-
BPAL-055	rpoC	c.517C>A	0.978723404255319	synonymous_variar	-
BPAL-055	mmpL5	p.Ile948Val	1.0	missense_variant	-
BPAL-055	mmpL5	p.Thr794Ile	1.0	missense_variant	-
BPAL-055	rpsL	c.-165T>C	1.0	upstream_gene_var	-
BPAL-055	rplC	c.-452C>A	1.0	upstream_gene_var	-
BPAL-055	Rv1129c	c.-28T>C	1.0	upstream_gene_var	-
BPAL-055	fbiC	p.Arg330Pro	1.0	missense_variant	-
BPAL-055	Rv1258c	c.1029T>C	1.0	synonymous_variar	-
BPAL-055	embR	p.Phe376Leu	0.246575342465753	missense_variant	-
BPAL-055	embR	p.Cys372Gly	0.233766233766233	missense_variant	-
BPAL-055	embR	p.Cys110Tyr	1.0	missense_variant	-
BPAL-055	embR	c.-207C>G	1.0	upstream_gene_var	-

BPAL-055	embR	c.-446C>T	1.0	upstream_gene_var	-
BPAL-055	atpE	c.-138T>C	1.0	upstream_gene_var	-
BPAL-055	rrs	n.-187C>T	1.0	upstream_gene_var	-
BPAL-055	rrl	n.982G>A	1.0	non_coding_transcr	-
BPAL-055	inhA	c.-40C>T	1.0	upstream_gene_var	-
BPAL-055	tsnR	c.369C>T	1.0	synonymous_variar	-
BPAL-055	tsnR	p.Leu232Pro	1.0	missense_variant	-
BPAL-055	tlyA	c.33A>G	1.0	synonymous_variar	-
BPAL-055	bacA	p.Ile603Val	1.0	missense_variant	-
BPAL-055	bacA	p.Trp233*	1.0	stop_gained	-
BPAL-055	katG	p.Arg463Leu	1.0	missense_variant	-
BPAL-055	PPE35	p.Leu896Ser	1.0	missense_variant	-
BPAL-055	Rv1979c	p.Asp286Gly	1.0	missense_variant	-
BPAL-055	Rv1979c	c.-129A>G	1.0	upstream_gene_var	-
BPAL-055	pncA	c.456C>G	1.0	synonymous_variar	-
BPAL-055	kasA	c.18C>T	1.0	synonymous_variar	-
BPAL-055	kasA	p.Gly312Ser	1.0	missense_variant	-
BPAL-055	ahpC	c.-142G>A	1.0	upstream_gene_var	-
BPAL-055	ald	c.-32T>C	1.0	upstream_gene_var	-
BPAL-055	fbiD	c.300A>G	1.0	synonymous_variar	-
BPAL-055	Rv3083	p.Asp71His	1.0	missense_variant	-
BPAL-055	whiB7	c.188delG	1.0	frameshift_variant	-
BPAL-055	lpqB	p.Asp142Gly	1.0	missense_variant	-
BPAL-055	mtrB	p.Met517Leu	1.0	missense_variant	-
BPAL-055	fbiA	c.15T>C	1.0	synonymous_variar	-
BPAL-055	clpC1	p.Val63Ala	1.0	missense_variant	-
BPAL-055	glpK	p.Val460Ala	1.0	missense_variant	-
BPAL-055	embC	p.Thr270Ile	1.0	missense_variant	-
BPAL-055	embC	p.Asn394Asp	1.0	missense_variant	-
BPAL-055	embC	c.2781C>T	1.0	synonymous_variar	-
BPAL-055	embA	c.348G>A	1.0	synonymous_variar	-
BPAL-055	embA	c.1188G>C	1.0	synonymous_variar	-
BPAL-055	embA	p.Pro913Ser	1.0	missense_variant	-
BPAL-055	embB	c.1065G>A	1.0	synonymous_variar	-
BPAL-055	embB	p.Glu378Ala	1.0	missense_variant	-
BPAL-055	ubiA	p.Glu149Asp	0.984126984126984	missense_variant	-
BPAL-055	ubiA	c.228T>C	1.0	synonymous_variar	-
BPAL-055	ubiA	c.-36delG	1.0	upstream_gene_var	-
BPAL-055	whiB6	p.Arg54Gln	1.0	missense_variant	-
BPAL-055	whiB6	c.-75delG	1.0	upstream_gene_var	-
BPAL-055	whiB6	c.-82C>T	1.0	upstream_gene_var	-
BPAL-055	whiB6	c.-211C>T	1.0	upstream_gene_var	-
BPAL-055	gid	c.615A>G	1.0	synonymous_variar	-
BPAL-055	gid	c.330G>T	1.0	synonymous_variar	-
BPAL-055	gid	p.Val36Gly	1.0	missense_variant	-
BPAL-056	dnaA	c.1302C>A	1.0	synonymous_variar	-
BPAL-056	gyrB	p.Met291Ile	1.0	missense_variant	-
BPAL-056	gyrA	p.Glu21Gln	1.0	missense_variant	-
BPAL-056	gyrA	p.Ser95Thr	1.0	missense_variant	-
BPAL-056	gyrA	p.Ala384Val	1.0	missense_variant	-
BPAL-056	gyrA	c.1842T>C	1.0	synonymous_variar	-
BPAL-056	gyrA	c.1959G>C	1.0	synonymous_variar	-
BPAL-056	gyrA	p.Gly668Asp	1.0	missense_variant	-
BPAL-056	Rv0010c	c.384T>C	1.0	synonymous_variar	-
BPAL-056	Rv0010c	p.Ile87Met	1.0	missense_variant	-
BPAL-056	Rv0010c	c.99T>C	1.0	synonymous_variar	-
BPAL-056	fgd1	c.960T>C	1.0	synonymous_variar	-
BPAL-056	mshA	c.21T>C	1.0	synonymous_variar	-
BPAL-056	Rv0565c	c.-108T>C	1.0	upstream_gene_var	-
BPAL-056	nusG	c.-138T>C	1.0	upstream_gene_var	-
BPAL-056	rpoB	c.3225T>C	1.0	synonymous_variar	-
BPAL-056	rpoC	c.162G>C	1.0	synonymous_variar	-

BPAL-056	rpoC	p.Ala172Val	1.0	missense_variant	-
BPAL-056	rpoC	c.517C>A	1.0	synonymous_variar	-
BPAL-056	mmpL5	p.Ile948Val	1.0	missense_variant	-
BPAL-056	mmpL5	p.Thr794Ile	1.0	missense_variant	-
BPAL-056	rpsL	c.-165T>C	1.0	upstream_gene_var	-
BPAL-056	rplC	c.-452C>A	1.0	upstream_gene_var	-
BPAL-056	Rv1129c	c.-28T>C	1.0	upstream_gene_var	-
BPAL-056	Rv1258c	c.1029T>C	1.0	synonymous_variar	-
BPAL-056	embR	p.Cys110Tyr	1.0	missense_variant	-
BPAL-056	embR	c.-207C>G	1.0	upstream_gene_var	-
BPAL-056	embR	c.-446C>T	1.0	upstream_gene_var	-
BPAL-056	atpE	c.-138T>C	1.0	upstream_gene_var	-
BPAL-056	rrs	n.-187C>T	1.0	upstream_gene_var	-
BPAL-056	rrl	n.982G>A	1.0	non_coding_transcr	-
BPAL-056	inhA	c.-40C>T	1.0	upstream_gene_var	-
BPAL-056	tsnR	c.369C>T	1.0	synonymous_variar	-
BPAL-056	tsnR	p.Leu232Pro	1.0	missense_variant	-
BPAL-056	tlyA	c.33A>G	1.0	synonymous_variar	-
BPAL-056	bacA	p.Ile603Val	1.0	missense_variant	-
BPAL-056	katG	p.Arg463Leu	1.0	missense_variant	-
BPAL-056	PPE35	p.Leu896Ser	1.0	missense_variant	-
BPAL-056	Rv1979c	p.Asp286Gly	1.0	missense_variant	-
BPAL-056	Rv1979c	c.-129A>G	1.0	upstream_gene_var	-
BPAL-056	kasA	c.18C>T	1.0	synonymous_variar	-
BPAL-056	kasA	p.Gly312Ser	1.0	missense_variant	-
BPAL-056	ahpC	c.-142G>A	1.0	upstream_gene_var	-
BPAL-056	Rv2477c	c.1119G>T	1.0	synonymous_variar	-
BPAL-056	ald	c.-32T>C	1.0	upstream_gene_var	-
BPAL-056	fbiD	c.300A>G	1.0	synonymous_variar	-
BPAL-056	Rv3083	p.Asp71His	1.0	missense_variant	-
BPAL-056	whiB7	c.188delG	1.0	frameshift_variant	-
BPAL-056	lpqB	p.Asp142Gly	1.0	missense_variant	-
BPAL-056	mtrB	p.Met517Leu	1.0	missense_variant	-
BPAL-056	fbiA	c.15T>C	1.0	synonymous_variar	-
BPAL-056	clpC1	p.Val63Ala	0.956521739130434	missense_variant	-
BPAL-056	glpK	p.Val460Ala	1.0	missense_variant	-
BPAL-056	embC	p.Thr270Ile	1.0	missense_variant	-
BPAL-056	embC	p.Asn394Asp	1.0	missense_variant	-
BPAL-056	embC	c.2781C>T	1.0	synonymous_variar	-
BPAL-056	embA	c.348G>A	1.0	synonymous_variar	-
BPAL-056	embA	c.1188G>C	1.0	synonymous_variar	-
BPAL-056	embA	p.Pro913Ser	1.0	missense_variant	-
BPAL-056	embB	c.1065G>A	1.0	synonymous_variar	-
BPAL-056	embB	p.Glu378Ala	1.0	missense_variant	-
BPAL-056	ubiA	p.Glu149Asp	0.971428571428571	missense_variant	-
BPAL-056	ubiA	c.228T>C	1.0	synonymous_variar	-
BPAL-056	ubiA	c.-36delG	1.0	upstream_gene_var	-
BPAL-056	ethR	c.-567C>A	1.0	upstream_gene_var	-
BPAL-056	whiB6	p.Arg54Gln	1.0	missense_variant	-
BPAL-056	whiB6	c.-75delG	1.0	upstream_gene_var	-
BPAL-056	whiB6	c.-82C>T	1.0	upstream_gene_var	-
BPAL-056	whiB6	c.-211C>T	1.0	upstream_gene_var	-
BPAL-056	gid	c.615A>G	1.0	synonymous_variar	-
BPAL-056	gid	c.330G>T	1.0	synonymous_variar	-
BPAL-059	rpoB	p.His445Tyr	1.0	missense_variant	rifampicin
BPAL-059	rrs	n.514A>C	1.0	non_coding_transcr	streptomycin
BPAL-059	inhA	c.-770T>C	1.0	upstream_gene_var	ethionamide;isoniazid
BPAL-059	katG	p.Ser315Thr	1.0	missense_variant	isoniazid
BPAL-059	embB	p.Gln497Arg	0.909090909090909	missense_variant	ethambutol
BPAL-059	dnaA	c.1302C>A	1.0	synonymous_variar	-
BPAL-059	gyrB	p.Met291Ile	1.0	missense_variant	-
BPAL-059	gyrA	c.-815C>T	1.0	upstream_gene_var	-

BPAL-059	gyrA	p.Glu21Gln	1.0	missense_variant	-
BPAL-059	gyrA	p.Ser95Thr	1.0	missense_variant	-
BPAL-059	gyrA	c.1959G>C	1.0	synonymous_variar	-
BPAL-059	gyrA	p.Gly668Asp	1.0	missense_variant	-
BPAL-059	Rv0010c	p.Ile87Met	1.0	missense_variant	-
BPAL-059	Rv0010c	c.99T>C	1.0	synonymous_variar	-
BPAL-059	fgd1	c.960T>C	1.0	synonymous_variar	-
BPAL-059	mshA	c.21T>C	1.0	synonymous_variar	-
BPAL-059	Rv0565c	c.-108T>C	1.0	upstream_gene_var	-
BPAL-059	nusG	c.-138T>C	1.0	upstream_gene_var	-
BPAL-059	rpoC	c.162G>C	1.0	synonymous_variar	-
BPAL-059	rpoC	p.Ala172Val	1.0	missense_variant	-
BPAL-059	rpoC	c.517C>A	1.0	synonymous_variar	-
BPAL-059	mmpL5	p.Ile948Val	0.9	missense_variant	-
BPAL-059	mmpL5	p.Thr794Ile	1.0	missense_variant	-
BPAL-059	rpsL	c.-165T>C	1.0	upstream_gene_var	-
BPAL-059	rplC	c.-452C>A	1.0	upstream_gene_var	-
BPAL-059	Rv1129c	c.-28T>C	1.0	upstream_gene_var	-
BPAL-059	embR	p.Phe376Leu	0.173913043478260	missense_variant	-
BPAL-059	embR	p.Cys372Gly	0.166666666666666	missense_variant	-
BPAL-059	embR	p.Cys110Tyr	1.0	missense_variant	-
BPAL-059	embR	c.-207C>G	1.0	upstream_gene_var	-
BPAL-059	embR	c.-446C>T	1.0	upstream_gene_var	-
BPAL-059	atpE	c.-138T>C	1.0	upstream_gene_var	-
BPAL-059	rrs	n.-187C>T	1.0	upstream_gene_var	-
BPAL-059	inhA	c.-973C>T	1.0	upstream_gene_var	-
BPAL-059	inhA	c.-40C>T	1.0	upstream_gene_var	-
BPAL-059	tsnR	c.369C>T	1.0	synonymous_variar	-
BPAL-059	bacA	p.Ile603Val	1.0	missense_variant	-
BPAL-059	katG	p.Arg463Leu	1.0	missense_variant	-
BPAL-059	PPE35	p.Leu896Ser	1.0	missense_variant	-
BPAL-059	Rv1979c	p.Asp286Gly	1.0	missense_variant	-
BPAL-059	Rv1979c	c.-129A>G	1.0	upstream_gene_var	-
BPAL-059	kasA	c.18C>T	1.0	synonymous_variar	-
BPAL-059	kasA	p.Gly312Ser	1.0	missense_variant	-
BPAL-059	ahpC	c.-142G>A	1.0	upstream_gene_var	-
BPAL-059	Rv2752c	p.Lys172Glu	1.0	missense_variant	-
BPAL-059	ald	c.-32T>C	1.0	upstream_gene_var	-
BPAL-059	fbiD	c.300A>G	1.0	synonymous_variar	-
BPAL-059	Rv3083	p.Asp71His	1.0	missense_variant	-
BPAL-059	whiB7	c.188delG	1.0	frameshift_variant	-
BPAL-059	lpqB	p.Asp142Gly	1.0	missense_variant	-
BPAL-059	mtrB	p.Met517Leu	1.0	missense_variant	-
BPAL-059	fbiA	c.15T>C	1.0	synonymous_variar	-
BPAL-059	clpC1	p.Val63Ala	1.0	missense_variant	-
BPAL-059	embA	c.348G>A	1.0	synonymous_variar	-
BPAL-059	ubiA	c.228T>C	1.0	synonymous_variar	-
BPAL-059	ubiA	c.-36delG	1.0	upstream_gene_var	-
BPAL-059	whiB6	p.Arg54Gln	1.0	missense_variant	-
BPAL-059	whiB6	c.-211C>T	1.0	upstream_gene_var	-
BPAL-059	gid	c.615A>G	1.0	synonymous_variar	-
